# BINOL as a Chiral Solvating Agent for Sulfiniminoboronic Acids

**DOI:** 10.1021/acs.analchem.3c01613

**Published:** 2023-11-06

**Authors:** Robin R. Groleau, Robert S. L. Chapman, John P. Lowe, Catherine L. Lyall, Gabriele Kociok-Köhn, Tony D. James, Steven D. Bull

**Affiliations:** † Department of Chemistry, University of Bath, Claverton Down, Bath BA2 7AY, U.K.; ‡ School of Chemistry and Chemical Engineering, Henan Normal University, Xianxiang 453007, China; § School of Chemistry, University of Leicester, Leicester LE1 7RH, U.K.

## Abstract

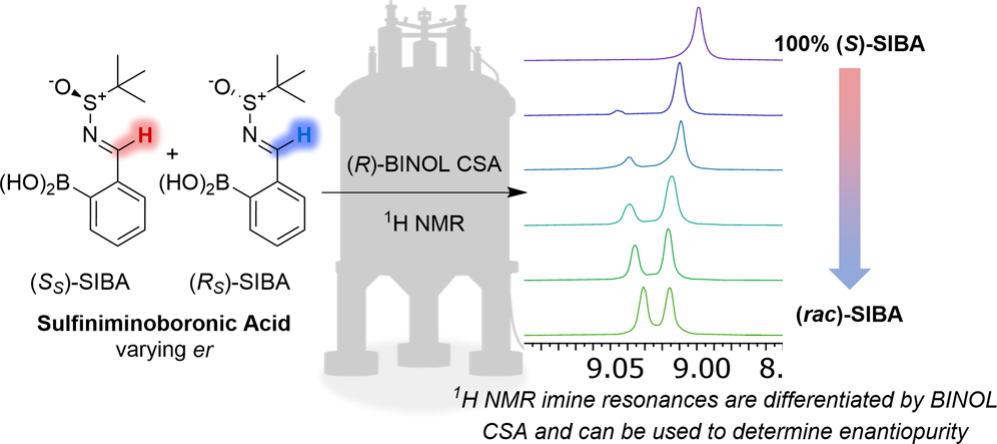

^1^H NMR spectroscopic studies using BINOL as a chiral solvating agent (CSA) for a scalemic sulfiniminoboronic acid (SIBA) have revealed concentration- and enantiopurity-dependent variations in the chemical shifts of diagnostic imine protons used to determine enantiopurity levels. ^11^B/^15^N NMR spectroscopic studies and X-ray structural investigations revealed that unlike other iminoboronate species, BINOL–SIBA assemblies do not contain N–B coordination bonds, with ^1^H NMR NOESY experiments indicating that intermolecular H-bonding networks between BINOL and the SIBA analyte are responsible for these variations. These effects can lead to diastereomeric signal overlap at certain er values that could potentially lead to enantiopurity/configuration misassignments. Consequently, it is recommended that hydrogen-bonding-CSA-based ^1^H NMR protocols should be repeated using both CSA enantiomers to ensure that any concentration- and/or er-dependent variations in diagnostic chemical shifts are accounted for when determining the enantiopurity of a scalemic analyte.

We have previously reported versatile three-component chiral derivatization protocols for determining enantiomeric excess (ee) of chiral amines, amino esters, diols, amino alcohols, diamines, hydroxyacids, diacids, and hydroxylamines by ^1^H/^19^F NMR spectroscopic analysis.^1−12^ In a representative protocol, treatment of a scalemic amine (e.g., α-methylbenzylamine **1**) with achiral 2-formylphenyl boronic acid (2-FPBA) and an enantiopure diol (e.g., BINOL) produces a mixture of diastereomeric iminoboronate esters (IBEs, e.g., **2a** and **2b**), whose diastereomeric ratio (dr) can then be determined by integration of baseline-resolved ^1^H NMR imine resonances. Since no kinetic resolution occurs in this derivatization process, these dr values are an accurate reflection of the enantiomeric ratio (er) of the parent scalemic amine analyte (Figure 1a).^1,13,14^ This widely used three-component derivatization method is often referred to as the Bull-James assembly,^1^ with this approach also having been used to determine er using circular dichroism-, fluorescence-, and electrochemical-based approaches.^15−17^ The excellent yields of IBEs produced in these self-assembly reactions have also been exploited to conduct efficient bioconjugation reactions and functionalize advanced polymeric materials.^1,15−18^

**Figure 1:**
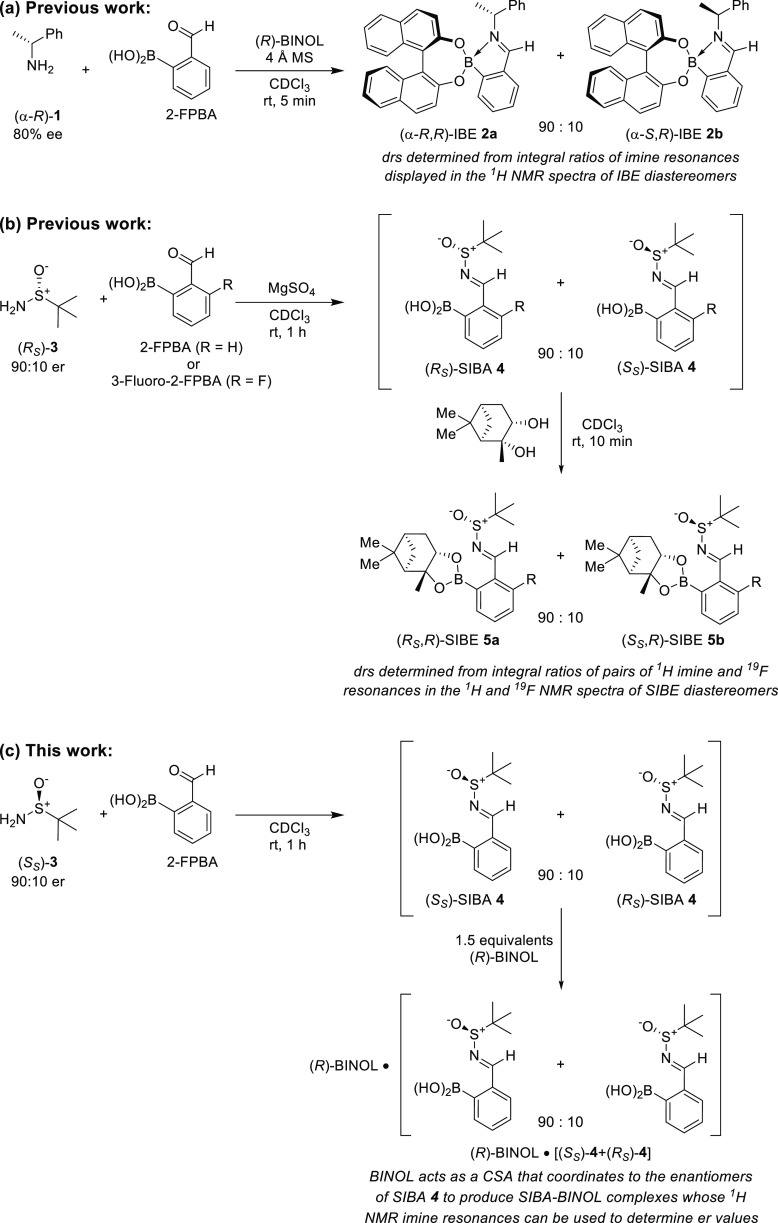
(a) Three-component complexation of scalemic amine (α-*R*)-**1** (90:10 er), 2-FPBA and enantiopure (*R*)-BINOL produces diastereomeric IBEs (α-*R*,*R*)-**2a** and (α-*S*,*R*)-**2b**, whose drs are determined by ^1^H NMR spectroscopic analysis.^13^ (b) Stepwise three-component complexation of scalemic sulfinamide (*R*_S_)-**3** (90:10 er), 2-FPBA, and enantiopure pinanediol produces diastereomeric SIBEs (*R*_S_,*R*)-**5a** and (*S*_S_,*R*)-**5b**, whose drs can be measured by ^1^H/^19^F NMR spectroscopic analysis.^19^ (c) Two-component reaction of scalemic Ellman’s sulfinamide (*S*_S_)-**3** (90:10 er) with 2-FPBA produces scalemic SIBA **4** whose enantiomers coordinate to (*R*)-BINOL to produce noncovalent (*R*)-BINOL**·**(*S*_S_)-SIBA **4** and (*R*)-BINOL**·**(*R*_S_)-SIBA **4** complexes.

We recently reported a modified version of this three-component CDA method for determining the enantiopurities of *S*-chiral sulfinamides using ^1^H/^19^F NMR spectroscopy.^19^ In this case, high-yielding formation of SulfinIminoBoronate Esters (SIBEs) required development of a modified two-step “one-pot” protocol to overcome the lower nucleophilicity of the sulfinamide amino group. This new method involved prior reaction of a scalemic sulfinamide (e.g., Ellman’s sulfinamide **3**) with 2-FPBA in CDCl_3_ to generate a scalemic SulfinIminoBoronic Acid (SIBA, e.g., **4**) that was then reacted with enantiopure pinanediol to afford a mixture of SIBE diastereomers (e.g., **5a** and **5b**) (Figure 1b). These SIBE diastereomers display well-resolved ^1^H NMR imine resonances, the integral ratios of which could be used to accurately determine dr values. Substituting 3-fluoro-2-FPBA for 2-FPBA in this complexation reaction allows the use of both ^1^H and ^19^F NMR spectroscopic analysis to accurately determine SIBE dr values using two independent measurements (Figure 1b).^19^

Optimization studies aimed at identifying the best chiral reporter diol to derivatize (*R*_S_)-SIBA **4** produced pairs of SIBE diastereomers that generally displayed well-resolved imine peaks in their ^1^H NMR spectra (Δδ_H_ = 0.010–0.085 ppm, Table S1). Pinanediol gave the best result, producing *tert*-butyl SIBE diastereomers **5a** and **5b** that displayed sharp ^1^H NMR imine peaks with a large Δδ_H_ value of 0.085 ppm. This led to pinanediol being chosen as the optimal chiral diol to derivatize other chiral sulfinamide analytes, whose SIBE diastereomers all displayed well-resolved ^1^H NMR imine peaks. (Table S2).^19^ However, derivatization of SIBA (*R*_S_)-**4** with (*R*)-BINOL gave inferior results, producing ^1^H NMR spectra exhibiting broader partially overlapped imine resonances. This result was surprising since previous three-component complexation reactions of (*rac*)-amines, 2-FPBA, and BINOL had produced diastereomeric IBEs displaying well-resolved ^1^H NMR imine resonances (e.g., Δδ_H_ = 0.17 ppm for **2a**/**2b**).^13^

These anomalous results prompted us to investigate the SIBA-BINOL complexation reaction further, with this study now reporting that reaction of SIBA **4** with BINOL does not in fact produce SIBE complexes like other diols. Instead, conformationally demanding BINOL (restricted rotation around biaryl bond) acts as a chiral solvating agent (CSA) to generate hydrogen-bonded BINOL–SIBA assemblies that display less well-resolved ^1^H NMR imine resonances (Figure 1c). Furthermore, investigations into the use of BINOL as a CSA for determining the enantiopurities of scalemic SIBA **4** samples revealed that intermolecular hydrogen-bonding effects produce concentration- and er-dependent variations in the diagnostic ^1^H NMR imine chemical shift values used to determine er values. These chemical shift variations can result in partially (or fully) overlapped ^1^H NMR imine resonances at certain analyte concentration and er levels that prevent er from being determined. This signal overlap issue can be resolved by repeating the process the opposite enantiomer of BINOL for derivatization to produce a new ^1^H NMR spectrum that displays fully resolved imine resonances.

The concentration- and er-dependent variations in diagnostic ^1^H NMR chemical shifts described in this study could potentially occur in other hydrogen-bonding-CSA-based ^1^H NMR protocols used to determine er values of other types of analyte. Therefore, it is recommended that duplicate ^1^H NMR spectra of scalemic analytes complexed to both CSA enantiomers should be acquired to determine whether er-dependent variations in diagnostic ^1^H NMR chemical shift values need to be accounted for when determining er values.

## Experimental Section

### General Experimental Details

Reagents and solvents were obtained from commercial suppliers and used without further purification. Reactions were performed without air exclusion or drying, at room temperature, and with magnetic stirring, unless otherwise stated. Anhydrous MgSO_4_ or Na_2_SO_4_ was used as a drying agent for organic solvents. Thin-layer chromatography (TLC) was carried out on Macherey–Nagel aluminum-backed plates that were precoated with silica. Compounds were visualized by either quenching of ultraviolet (UV) fluorescence at 254 nm or by dip-staining (KMnO_4_, PMA, curcumin,^20^ I_2_) followed by gentle heating. Purification by flash column chromatography was performed using high-purity-grade silica gel (60 Å pore size, 40–75 μm particle size). PE refers to petroleum ether 40–60 °C. Capillary melting points were determined using a Stuart digital SMP10 melting point apparatus and are reported uncorrected to the nearest °C. Optical rotations were measured using an Optical Activity Ltd. AA-10 Series Automatic Polarimeter, with a path length of 1 dm and with concentration (*c*) quoted in g/100 mL. Nuclear Magnetic Resonance (NMR) spectroscopy experiments were performed in deuterated solvents at 298 K (unless otherwise stated) on either a Bruker 500 MHz spectrometer or an Agilent ProPulse 500 MHz spectrometer. ^1^H, ^13^C, ^11^B, and ^19^F NMR chemical shifts (δ) are quoted in parts per million (ppm) and are referenced to either the residual solvent peak or tetramethylsilane (TMS) when possible.^21^^11^B NMR spectra were referenced directly, using the lock signal, to external BF_3_.Et_2_O (0 ppm). Coupling constants (*J*) are given in Hz. In those cases where ^13^C signals could not be observed by one-dimensional (1D) NMR spectroscopy due to low solubility, adjacent quadrupolar nuclei, or lack of adjacent ^1^H nuclei, then chemical shifts were measured indirectly from two-dimensional (2D) ^1^H–^13^C HMBC experiments.^19^^15^N NMR chemical shifts were measured indirectly from 2D ^1^H–^15^N HMBC spectra using a Bruker Avance 500 MHz spectrometer.^22^^15^N NMR experiments were carried out at 50 mM sample concentrations. ^15^N NMR spectroscopy was carried out in CDCl_3_ containing 50 mM nitromethane internal standard. The MeNO_2_^15^N resonance was used as the reference for ^15^N chemical shifts, setting δ_N_(MeNO_2_) = 0.00 ppm.^23^ Infrared (IR) spectra were recorded using a PerkinElmer Spectrum 100 Fourier-transform infrared (FTIR) spectrometer fitted with a Universal ATR FTIR accessory, with samples run neat and selected absorbances quoted as ν in cm^–1^. High-resolution mass spectrometry (HRMS) results were acquired on an externally calibrated Bruker Daltonics maXis HD ultrahigh-resolution-time of flight (UHR-TOF) mass spectrometer coupled to an electrospray source (ESI-TOF) or an Agilent QTOF 6545 with Jetstream ESI. In most cases, molecular ions were detected either in positive mode as their protonated, sodiated, or ammonium adduct forms, or in negative mode as deprotonated or acetate adduct species.

Additional details of materials, reagents, and instrumentation are provided in the Supporting Information. Additionally, full synthetic procedures for the preparation of all compounds reported herein, along with full characterization data including NMR spectra, can also be found in the Supporting Information.

### Three-Component Derivatization of α-Methylbenzylamine 1 with 2-FPBA and (*R*)-BINOL

Enantiopure (*R*)- or (*S*)-α-methylbenzylamine **1** in CDCl_3_ (1.0 mL, 0.10 M containing ∼6 mM TMS internal standard) was added to 2-FPBA (15 mg, 0.10 mmol, 1.0 equiv) and (*R*)-BINOL (31.5 mg, 0.11 mmol, 1.1 equiv). The reaction mixture was stirred for 10 min at rt before an aliquot (650 μL) was removed, and NMR spectra of the resultant iminoboronate esters **2a**/**2b** were acquired.

### “One-Pot” Stepwise Three-Component Assembly of Sulfinamide 3, 2-FPBA, and Pinanediol

2-FPBA (0.12 mmol, 1.2 equiv) and anhydrous MgSO_4_ (200 mg) were added to a stirred solution of Ellman’s sulfinamide **3** (0.1 mmol, 1.0 equiv) in CDCl_3_ (1.0 mL, ∼6 mM TMS internal standard). The reaction mixture was stirred for 1 h at rt, and pinanediol (22 mg, 0.13 mmol, 1.3 equiv) was then added. The reaction was stirred for a further 10 min before being filtered, and the NMR spectra of a 650 μL aliquot were then acquired.

### Three-Component Stepwise Derivatization of *tert*-Butylsulfinamide 3 with 2-FPBA and BINOL

Ellman’s sulfinamide **3** (1.0 mL, 0.1 M in CDCl_3_ with ∼6 mM TMS) of known enantiopurity was added to a mixture of 2-FPBA (15 mg, 0.10 mmol, 1.0 equiv) and enantiopure (*R*)- or (*S*)-BINOL (variable amount per sample). The resulting solution was stirred for 1 h at rt, with a 650 μL aliquot then removed and its NMR spectra acquired.

Scalemic and racemic samples of Ellman’s sulfinamide **3** were prepared by combining different amounts of enantiopure solutions of (*R*)- and (*S*)-sulfinamide **3** in CDCl_3_. Samples required for concentration screening experiments were prepared directly from 100 mM solutions by dilution, as required.

^1^H–^15^N HMBC spectra were acquired by using 50 mM solutions of the desired compound in CDCl_3_ containing 50 mM MeNO_2_ as an internal standard.

## Results and Discussion

**Figure 2:**
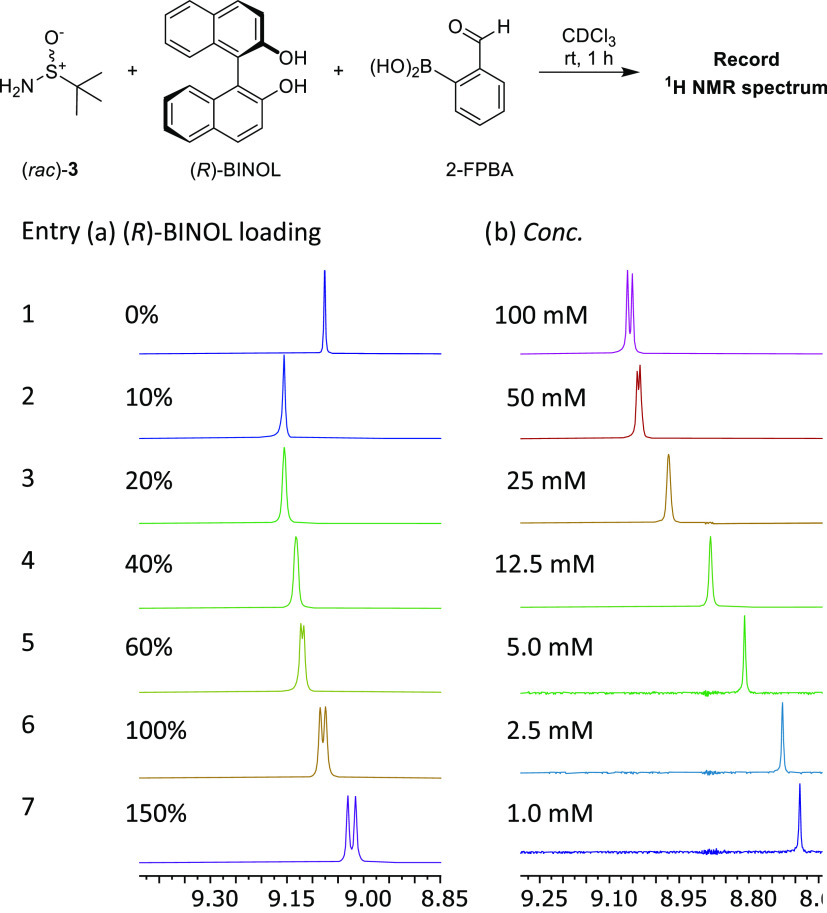
Expanded imine region of the ^1^H NMR spectra (500 MHz, CDCl_3_) of: (a) (*R*)-SIBA **4** (100 mM), recorded in the presence of increasing amounts of (*R*)-BINOL from 0% (top) to 150% loading (bottom); (b) (*rac*)-SIBA **4** and 100 mol % (*R*)-BINOL in CDCl_3_, acquired at decreasing concentrations from 100 mM (top) to 1.0 mM (bottom). Chemical shifts referenced to TMS as an internal standard (∼6 mM).

Screening experiments revealed that complexation of (*rac*)-SIBA **4** with enantiopure BINOL in CDCl_3_ at a 100 mM concentration produced ^1^H NMR spectra displaying broad and partially overlapped imine resonances.^19^ This result contrasted with the complexation reactions of (*rac*)-SIBA **4** with other diols (previously reported by us),^19^ which gave ^1^H NMR spectra of SIBE complexes displaying sharp and well-resolved imine resonances in most cases (see Table S1).^1,19^ Further investigations revealed that treatment of (*rac*)-SIBA **4** with (*R*)-BINOL gave ^1^H NMR spectra whose imine chemical shifts and Δδ_H_ values varied according to the stoichiometry of (*R*)-BINOL used (Figure 2a and Table S3). For example, ^1^H NMR spectra of (*rac*)-SIBA **4** treated with ≤40 mol % (*R*)-BINOL displayed a single unresolved ^1^H NMR imine peak (Figure 2a, entries 1–4), while 60–150 mol % (*R*)-BINOL loadings produced increasingly differentiated ^1^H NMR imine peaks, whose averaged chemical shift value drifted incrementally upfield from δ_H_ 9.120 to δ_H_ 9.023 ppm (Figure 2a, entries 5–7).

These (*R*)-BINOL concentration-dependent increases in imine Δδ_H_ values were initially unexpected since the ^1^H NMR spectra of other SIBA-diol complexes did not display concentration-dependent imine chemical shift variations.^1,19^ This led us to conclude that treatment of SIBA **4** with BINOL does not produce IBEs like with other diols. Instead, a change in reaction manifold occurs whereby BINOL serves as a hydrogen-bonding CSA to differentiate the SIBA enantiomers (Figure 1c). Further evidence for BINOL acting as a hydrogen-bonding CSA for **4** is also evident from the gradual downfield shift of the BINOL–OH resonance as the concentration of BINOL increases, and the broader imine resonances produced in the ^1^H NMR spectra of these BINOL systems compared to typical SIBE/IBE CDA assemblies, also typical of a hydrogen-bonding system.

A review of the literature has revealed significant precedent for the use of BINOL as a hydrogen-bonding CSA to determine the er values of chiral amines, sulfinimines, alcohols, sulfoxides, acids, amino alcohols, and alkaloids.^24−32^ In order to confirm that BINOL was functioning as a CSA in our case, we next carried out dilution experiments on solutions of (*rac*)-SIBA **4** and 1 equiv of (*R*)-BINOL in CDCl_3_, which revealed significant variation in the chemical shifts of the diagnostic imine as the sample was diluted (Figure 2b and Table S4).^33,34^^1^H NMR spectroscopic analysis at concentrations decreasing from 100 to 50 mM gave partially resolved imine peaks centered at δ_H_ 9.056 and 9.037 ppm, respectively (Figure 2b, Entries 1 and 2), while samples recorded at concentrations of ≤25 mM displayed only a single unresolved imine singlet at δ_H_ ≤ 8.973 ppm (Figure 2b, Entries 3–7). Corresponding upfield chemical shift drift of the imine peaks of enantiopure (*S*_S_)/(*R*_*S*_)-SIBA **4** complexed to (*R*)-BINOL was also observed on sample dilution (see Figure S1 and Table S5).

The concentration-dependent changes in chemical shift values and imine peak resolution in these complexation reactions are all consistent with greater noncovalent interactions occurring between the SIBA enantiomers and BINOL at greater BINOL loadings and higher sample concentrations. Further evidence that BINOL was acting as a CSA in CDCl_3_ was obtained by treating SIBA **4** (varying er)^35^ with 100 mol % BINOL in CD_3_CN. These reactions produced concentration-independent ^1^H NMR spectra displaying a single unresolved imine peak at δ_H_ 8.73 ppm, which is consistent with the more polar coordinating CD_3_CN solvent preventing H-bonding interactions from forming between the SIBA enantiomers and BINOL, thus not inducing enantiodiscrimination (see Figure S4).

**Figure 3:**
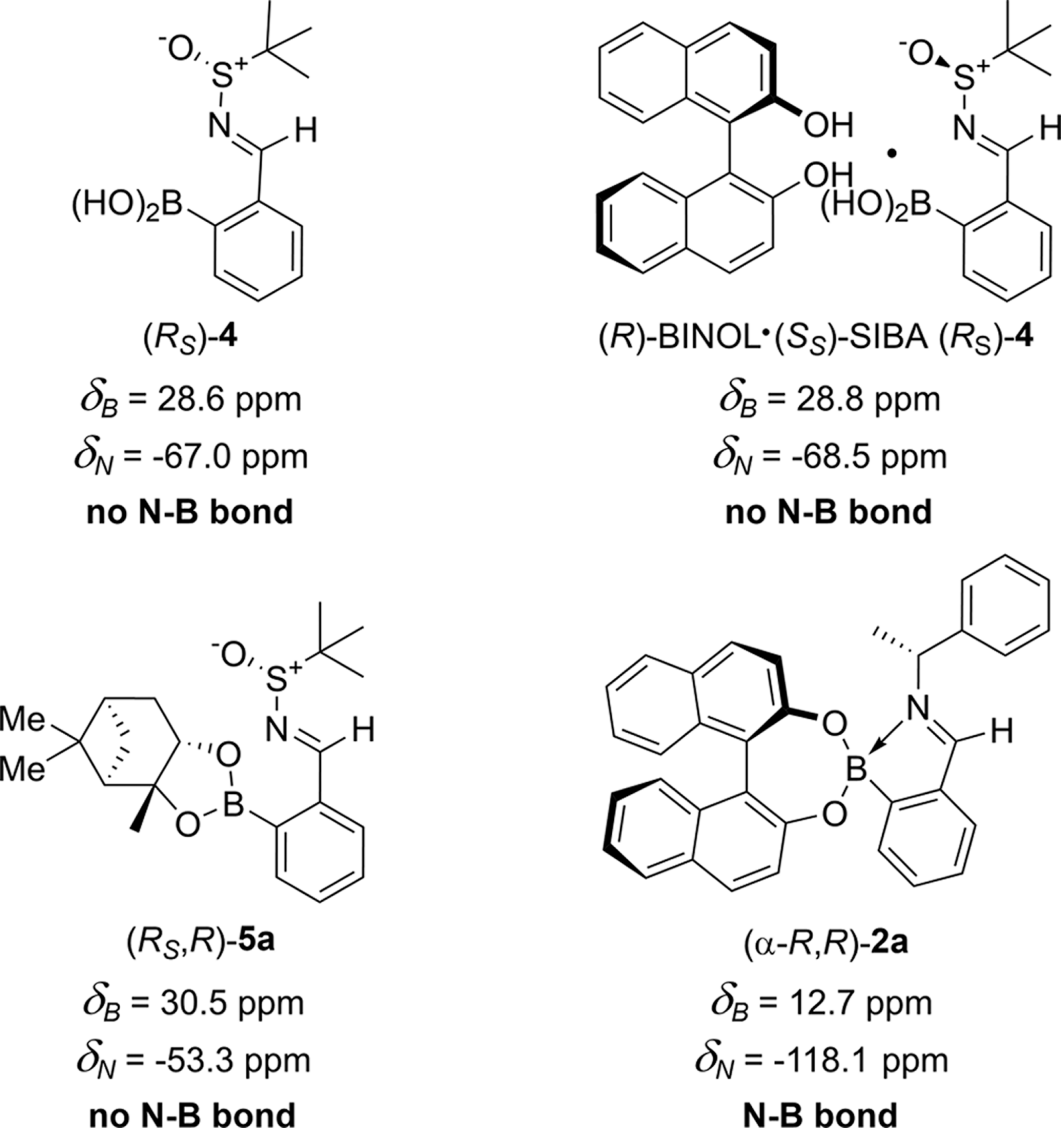
^11^B and ^15^N NMR chemical shifts of selected SIBA, SIBE, and IBE complexes. See Tables S10 and S11 for the full data set and benchmarking studies. δ_N_ is referenced to MeNO_2_.

**Figure 4:**
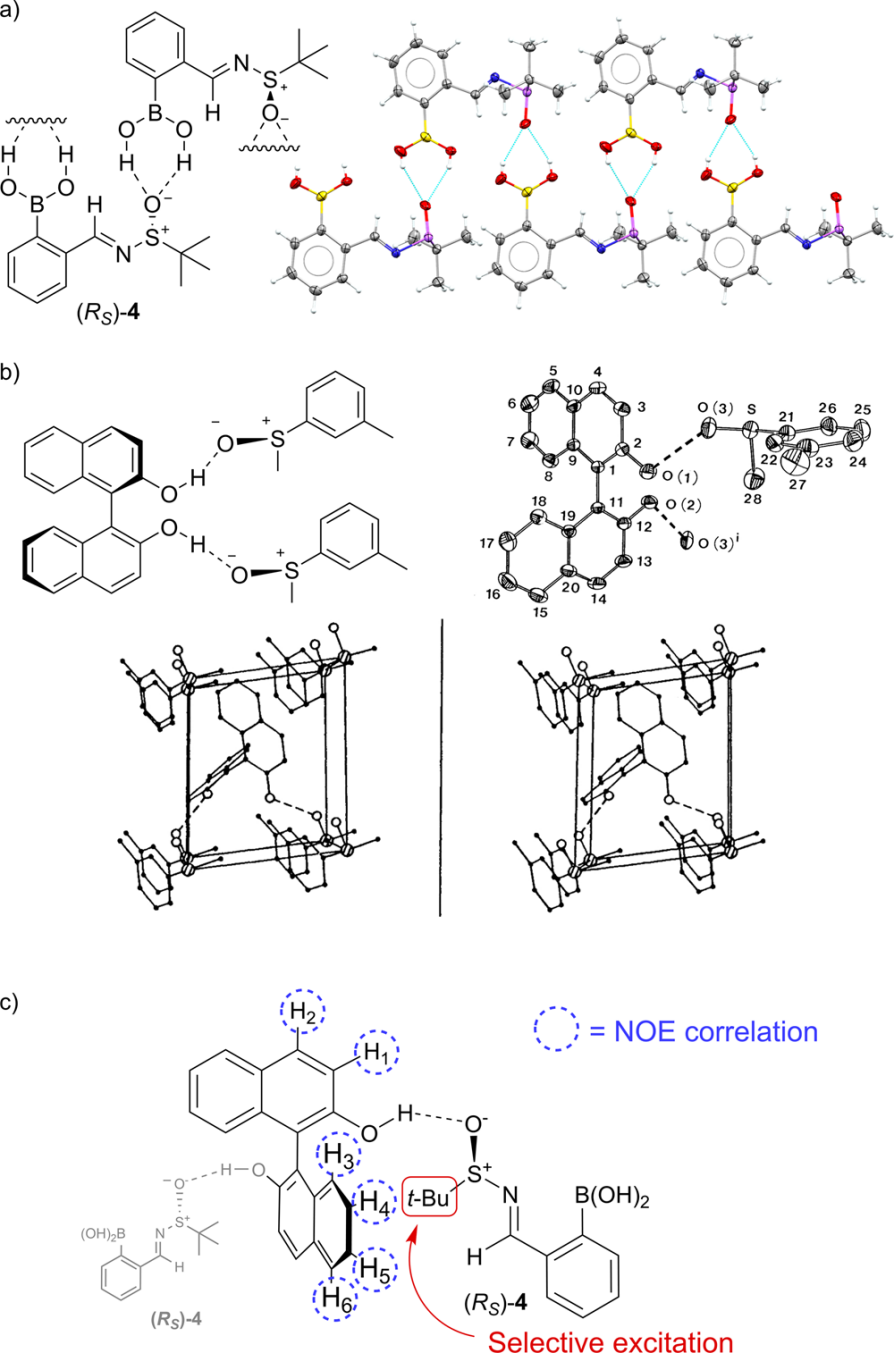
(a) X-ray crystal structure of SIBA (*R*_S_)-**4** showing intermolecular hydrogen-bonded ladders formed by the boronic acid moiety of one SIBA unit with the sulfoxide oxygen of another SIBA unit. (b) Hydrogen-bonding networks present in the X-ray crystal structures of (*R*_S_)-*m*-tolyl methyl sulfoxide complexed to BINOL. (Reproduced from ref (43) with permission from The Japanese Chemical Society). (c) Evidence of hydrogen bonding between BINOL and (*R*_S_)-**4** in solution from ^1^H NMR NOE interactions between the protons of the *tert*-butyl group of SIBA **4** and the aromatic protons of BINOL (see Figure S57 for spectra). H-bonds are represented by broken lines.

To investigate the structural reasons for this change in complexation manifold, ^11^B and ^15^N NMR spectroscopic analyses on samples of SIBA (*R*_S_)-**4**, SIBE **5a**, [SIBA (*R*_S_)-**4**·(*R*)-BINOL], and BINOL-derived IBE **2a** in CDCl_3_ were then carried out to determine which systems contained N→B coordination bonds (Figure 3, see Table S10 for full data set, including other diastereomers). δ_N_ chemical shift values were determined indirectly through analysis of ^1^H–^15^N HMBC spectra (Tables S10 and S11).^22,36^ SIBA (*R*_S_)-**4** in CDCl_3_ exhibited ^11^B and ^15^N NMR resonances at δ_B_ = 28.6 ppm and δ_N_ = −67.0 ppm that are consistent with (*R*_S_)-**4** containing a planar sp^2^-hybridized boron atom and a noncoordinated imine nitrogen atom. The absence of an N→B coordination bond in (*R*_S_)-**4** in the solid state was confirmed by X-ray crystallographic analysis (see Figures 4a and S61). A mixture of SIBA (*R*_S_)-**4** and 150 mol % (*R*)-BINOL in CDCl_3_ gave comparable chemical shift values of δ_B_ = 28.8 ppm and δ_N_ = −68.5 ppm, consistent with any [SIBA (*R*_S_)-**4**·(*R*)-BINOL] complexes formed containing noncoordinated sp^2^-hybridized boron and nitrogen atoms. Pinanediol-derived SIBE (*R*_S_,*R*)-**5a** (strong [M + H]^+^ HRMS peak at *m*/*z* 388.2118) gave chemical shift values of δ_B_ = 30.5 ppm and δ_N_ = −53.3 ppm, once again consistent with the presence of noncoordinated sp^2^-hybridized boron and nitrogen atoms. Conversely, BINOL-derived (α-*R*,*R*)-IBE **2a** gave significantly upfield chemical shift values of δ_B_ = 12.7 ppm and δ_N_ = −118.1 ppm, consistent with the presence of coordinated sp^3^-hybridized boron and nitrogen atoms. The presence of intramolecular N→B coordination bonds in BINOL-derived (α-*S*,*S*)-IBE **2a** and (α-*S*,*R*)-IBE **2b** in the solid state was also confirmed by X-ray crystallographic analysis (see Figures S58 and S59).^1,37,38^

The absence of a stabilizing intramolecular N→B coordination bond in pinanediol-derived SIBE **5a** is likely due to steric congestion and/or the electron-withdrawing N-sulfinyl group preventing electron density from being donated from its imine lone pair into the empty p orbital of the boron atom. This contrasts with BINOL-derived (α-*R*,*R*)-IBE **2a** whose structure contains an intramolecular N→B coordination bond that generates sp^3^-hybridized boron and nitrogen atoms.

This absence of an intramolecular N→B coordination bond in pinanediol-derived SIBE **5a** also provides an explanation for the inability of BINOL to form SIBE complexes. BINOL-SIBE formation would require incorporation of the conformationally restricted diol unit of BINOL (hindered rotation around the biaryl bond) into a strained seven-membered cyclic boronate ester ring. Furthermore, the absence of a stabilizing N→B bond means that this ring system would also need to accommodate a planar sp^2^-hybridized boron atom, thus introducing further strain into an already strained ring system. Therefore, it follows that the extra strain energy required to incorporate the diol unit of BINOL and a planar sp^2^ boron atom into a SIBE complex means that BINOL prefers to act as a H-bonding CSA to produce BINOL-coordinated SIBA complexes.^24−31^

Further evidence that BINOL does not readily react with aryl boronic acids to produce cyclic boronate esters was acquired by carrying out complexation reactions of *m*-tolylboronic acid and 2-FPBA with pinanediol and BINOL (1.50 equiv) in CDCl_3_. ^1^H NMR spectroscopic analysis of these simple complexation reactions revealed that only pinanediol was capable of producing cyclic boronate esters under these conditions, with BINOL showing no reaction with either boronic acid substrate (Scheme S1).^39−41^

Crystals of BINOL–SIBA complexes could not be obtained, however, X-ray crystal analysis of (*R*_S_)-SIBA **4** revealed the presence of strong intermolecular hydrogen bonds between the sulfinyl oxygen atom of one SIBA unit and the boronic acid protons of another to produce well-defined 4-membered Ar–B(OH)_2_···^–^O-^+^S-R hydrogen-bonding assemblies (Figure 4a). Previous X-ray crystallographic studies by Toda et al. have also shown that intermolecular hydrogen-bonding interactions are formed between the phenolic units of BINOL and the sulfinyl oxygen of analogous (*R*_S_)-*m*-tolyl methyl sulfoxide, which combine to produce hydrogen bond networks containing “infinite zigzag chains” (Figure 4b).^42−44^

Further evidence for strong intermolecular H-bonding interactions between BINOL and SIBA (*R*)-**4** in CDCl_3_ was obtained from selective 1D ^1^H NOE spectroscopic studies on equimolar BINOL–SIBA mixtures in CDCl_3_. Selective excitation of the *tert*-butyl protons of SIBA (*R*)-**4** revealed significant NOE interactions with all six ^1^H NMR aromatic environments of (*R*)-BINOL. These NOE interactions are consistent with a hydrogen bond between the sulfinyl oxygen of a SIBA unit (acceptor) and a phenolic group of a BINOL unit (donor) serving to hold the SIBA *tert*-butyl group and the BINOL aromatic protons in close proximity (Figure 4c).

**Figure 5:**
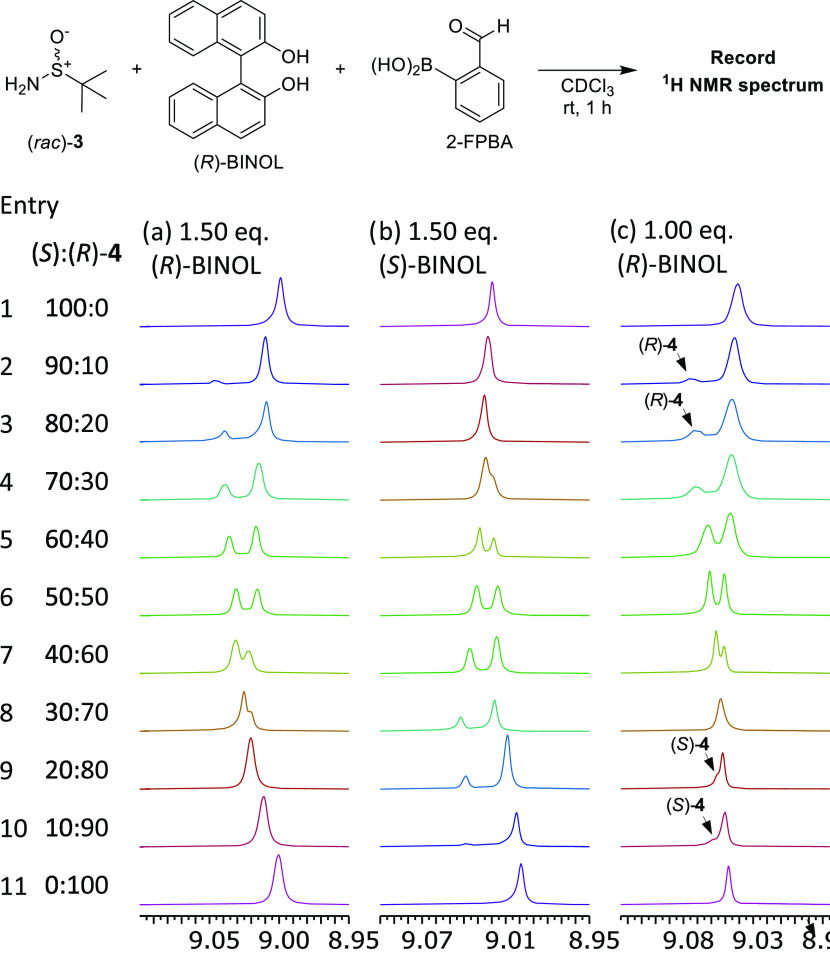
Expanded imine regions of the ^1^H NMR spectra (500 MHz, CDCl_3_, 100 mM) of SIBA **4** of varying er (100% (*S*)-**4** to 100% (*R*)-**4** in 10% er increments from top to bottom) in the presence of: (a) 1.50 eq. loading of (*R*)-BINOL; (b) 1.50 equiv loading of (*S*)-BINOL; and (c) 1.00 equiv loading of (*R*)-BINOL. Chemical shifts referenced to TMS as an internal standard (∼6 mM). Partially resolved minor imine peaks are highlighted with arrows.

Having established that BINOL was acting as a hydrogen-bonding CSA, we next explored its potential for determining the ers of scalemic samples of SIBA **4**. Scalemic samples of SIBA **4** (10% er increments from (*S*_S_)-**4** to (*R*_S_)-**4**) were treated with 1.50 equiv (maximum solubility level)^45^ of (*R*)-BINOL at a 100 mM concentration in CDCl_3_. Examination of these ^1^H NMR spectra revealed er-dependent variations in the chemical shifts of the diagnostic imine resonances of these scalemic samples (Figure 5a and Table S6). Treatment of (*S*_S_)-**4** and (*R*_S_)-**4** with (*R*)-BINOL gave ^1^H NMR spectra displaying imine signals with essentially identical chemical shift values at δ_H_ = 8.999 and δ_H_ = 9.000 ppm, respectively (Figure 5a, entries 1 and 11). This is in contrast to the chemical shift values observed for (*rac*)-SIBA **4** and (*R*)-BINOL, which displayed resolved imine peaks at higher δ_H_ = 9.015 and δ_H_ = 9.030 ppm values, respectively (Figure 5a, entry 6). Increasing the er of the SIBA analyte from 60_(*S*)_:40_(*R*)_ to 90_(*S*)_:10_(*R*)_ er resulted in the chemical shift of the minor (*R*)-imine resonance shifting incrementally downfield from δ_H_ 9.035 to 9.045 ppm, and the major (*S*)-imine resonance moving incrementally upfield from δ_H_ 9.017 to 9.009 ppm (Figure 5a, entries 5–2). These er-dependent changes in chemical shift produce a corresponding increase in Δδ_H_ value from −0.018 ppm for a racemic sample to −0.036 ppm for a 90_(*S*)_:10_(*R*)_ er sample (Figure 5a, cf. entries 6 and 2). However, regardless of their overall Δδ_H_ values, integration of fully resolved major and minor imine peaks of each of these scalemic samples enabled their er values to be accurately determined.

**Figure 6:**
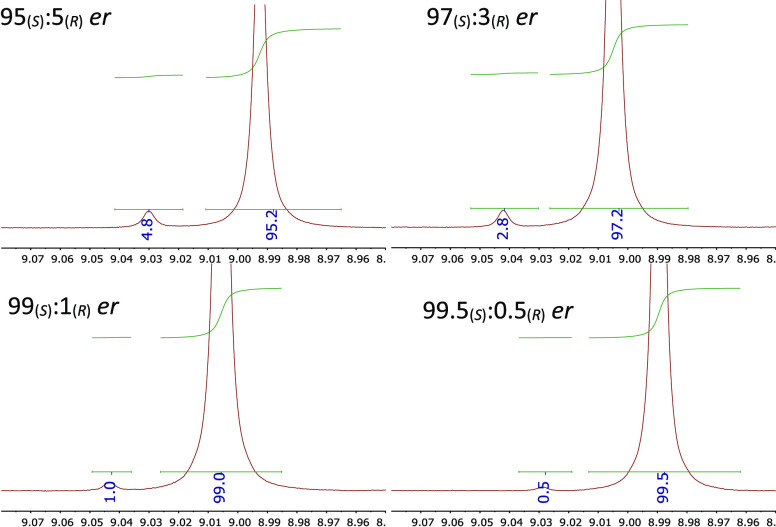
Expanded imine regions of the ^1^H NMR spectra (500 MHz, CDCl_3_, 100 mM) of scalemic SIBA **4** with high 95_(*S*)_:5_(*R*)_-99.5_(*S*)_:0.5_(*R*)_ er levels in the presence of 1.50 equiv (*R*)-BINOL. Chemical shifts referenced to TMS as an internal standard (∼6 mM). Measured integral values are within the accepted margins of error. See Figure S6 for enhanced spectra containing insets of minor imine signals.

Conversely, acquisition of ^1^H NMR spectra of (*R*)-BINOL complexes of scalemic SIBA samples of 40_(*S*)_:60_(*R*)_ and 30_(*S*)_:70_(*R*)_ er revealed increasing overlap of the diastereomeric imine peaks, which coalesced into a single peak at ≤20_(*S*)_:80_(*R*)_ er (Figure 5a, Entries 7–10), meaning that their er levels could not be determined. However, repeating the ^1^H NMR study using (*S*)-BINOL (opposite CSA enantiomer) allowed this er-dependent imine overlap issue to be resolved (Figure 5b and Table S7). Acquisition of a new set of ^1^H NMR spectra using (*S*)-BINOL as the CSA produced a new series of ^1^H NMR spectra that displayed er-dependent variations in the chemical shifts of the major and minor imine peaks that mirrored those produced previously using (*R*)-BINOL (cf. Figure 5a,5b). This means that (*R*)-BINOL could be used as a CSA to accurately determine the ers of SIBA samples ranging from 50_(*S*)_:50_(*R*)_ to >99_(*S*)_:1_(*R*)_ er, while (*S*)-BINOL could be used to accurately determine the ers of SIBA samples ranging from 50_(*S*)_:50_(*R*)_ to <1_(*S*)_:99_(*R*)_ er. Detection limits of this CSA system were determined by treating scalemic SIBA **4** samples ranging from 95_(*S*)_:5_(*R*)_ to >99_(*S*)_:1_(*R*)_ er with 1.50 equiv of (*R*)-BINOL, whose corresponding ^1^H NMR spectra all gave accurate er values (up to 99.5_(*S*)_:0.5_(*R*)_ er, i.e., a 99% ee detection limit, Figures 6 and S6). Examination of the ^1^H NMR spectra produced using (*S*)- and (*R*)-BINOL revealed that they were not exactly mirrored, with very small variations likely due to minor changes in impurity profiles or moisture content subtly affecting the hydrogen-bonding networks that are responsible for enantiodiscrimination in this system.

It was subsequently found that reducing the amount of (*R*)-BINOL used in the er-dependent variation studies of SIBA **4** to one equivalent resulted in a remarkable crossover event occurring in the chemical shift values of the major and minor imine peaks as the er of the SIBA analyte was varied (Figure 5c and Table S8). As seen previously, variation in the chemical shifts of the ^1^H NMR imine peaks was observed for er values between 90_(*S*)_:10_(*R*)_ and 50_(*S*)_:50_(*R*)_ er, with minor imine peaks of the (*R*_*S*_)-SIBA enantiomers appearing downfield of the major imine peaks of the (*S*_*S*_)-enantiomers (Figure 5c, Entries 2–6). Once again, incremental changes from 50_(*S*)_:50_(*R*)_ to 30_(*S*)_70_(*R*)_ er resulted in increasing overlap of the major and minor imine peaks, which coalesced into a single broad imine peak at 30_(*S*)_:70_(*R*)_ er (Figure 5c, Entry 8). However, derivatization of SIBA samples of ≤20_(*S*)_:80_(*R*)_ er gave ^1^H NMR spectra where the major imine peaks of the (*R*_S_)-enantiomers were now upfield to the minor imine peaks of their (*S*_S_)-enantiomers (Figure 5c, Entries 9 and 10, imine peaks partially overlapped). This means that an er-dependent switch in the Δδ_H_ sign of the imine resonances occurs in this system, with (*R*)-BINOL producing a large negative Δδ_H_ value of −0.030 ppm for a 90_(S)_:10_(R)_ er sample (Figure 5c, entry 2), but a small opposing positive Δδ_H_ value of +0.007 ppm value for a 10_(S)_:90_(R)_ er value (Figure 5c, entry 10). The results of this er determination study were verified by carrying out complementary complexation studies using (*S*)-BINOL as the CSA, which gave the expected mirrored results (see Figure S5 and Table S9 for details).

The multicomponent intermolecular hydrogen-bonding networks that are proposed in these BINOL–SIBA complexes provide a simple explanation to understand the er-dependent variations in ^1^H NMR imine chemical shift values that occur as the enantiopurity of the SIBA analyte is varied. The chemical shift values of the imine protons of each BINOL-coordinated SIBA enantiomer will be determined by the cumulative intramolecular and intermolecular shielding and deshielding effects that they experience. These intermolecular hydrogen-bonding networks are likely formed from interactions between the phenol groups of BINOL units and the sulfinyl and boronic acid groups of the SIBA analyte. This means that different scalemic SIBA samples will contribute different relative amounts of (*R*)- and (*S*)- enantiomers to their respective hydrogen bond networks, resulting in variable intermolecular imine shielding/deshielding effects being present in different scalemic samples.

We believe these concentration- and er-dependent variations in chemical shift values have important implications for how hydrogen-bonding-CSA-based NMR protocols should be bechmarked, with a lack of appreciation for these effects potentially leading to incorrect er values or absolute configurations being assigned. For example, the use of (*R*)-BINOL as a CSA for a SIBA sample of 10_(*S*)_:90_(*R*)_ er produces a ^1^H NMR spectrum containing a single imine peak that could be misinterpreted as evidence that this SIBA sample was enantiopure. In contrast, the ^1^H NMR spectrum of the same SIBA sample 10_(S)_:90_(R)_ er in the presence of (*S*)-BINOL displays a set of well-resolved major and minor imine peaks, whose imine integral ratio is an accurate reflection of the enantiopurity of the sample (cf. Entries 10 of Figure 5a,5b). Alternatively, comparison of the ^1^H NMR spectra of SIBA samples of 90_(s)_:10_(R)_ er and 10_(S)_:90_(R)_ er in the presence of 1.00 equiv BINOL (Figure 5c, cf. Entries 2 and 9) reveals that their minor imine peaks are more deshielded in both cases. This could easily be misconstrued to mean that the same SIBA enantiomer is present in excess in both samples, with this example clearly illustrating the potential pitfalls of using the sign of Δδ_H_ values to assign the absolute configuration of chiral analytes in hydrogen-bonding CSA systems.

Given the findings described herein, we now recommend that new hydrogen-bonding-CSA-based NMR protocols used to determine the ers of chiral analytes should be repeated using both enantiomers of the CSA to generate duplicate sets of ^1^H NMR spectra at a fixed concentration and stoichiometry. These complementary ^1^H NMR spectra should then be compared to check for the presence of any er*-*dependent variations in diagnostic chemical shifts, thus ensuring that the correct CSA enantiomer is chosen to determine accurate er values or absolute configurations. Furthermore, the dramatic variations in chemical shift observed in this hydrogen-bonding CSA NMR study clearly illustrate the importance of reporting NMR sample concentration to ensure the accuracy and reproducibility of er determination protocols. We would also like to note that these variations can also occur to a lesser extent in CDA protocols that produce derivatized species that are capable of forming hydrogen-bonding networks or other noncovalent interactions, so similar precautions should also be taken in these cases.^46−48^

## Conclusions

This study reports that the enantiomers of SIBA **4** coordinate to BINOL (acting as a CSA) in CDCl_3_ to produce diastereomeric hydrogen-bonded BINOL–SIBA complexes whose imine protons are distinguishable in their ^1^H NMR spectra. The inability of BINOL to produce a three-component SIBE complex (like other diols) is due to the difficulty of incorporating its conformationally restricted diol unit and a planar sp^2^ boron atom into a strained seven-membered boronate ester ring. Additionally, SIBAs and SIBEs were found to lack the N→B bond typical of IBEs, which further disfavors the incorporation of BINOL. The use of BINOL as a CSA to determine the ers of scalemic SIBA **4** samples revealed concentration- and er-dependent variations in diagnostic imine ^1^H NMR chemical shift values that can result in imine peak overlap for selected er values. This imine peak overlap issue can be resolved by repeating the protocol using the opposite enantiomer of BINOL as the CSA. This adaptation enables BINOL to be used to accurately determine the enantiopurity levels of scalemic samples of SIBA **4** (and hence Ellman’s sulfinamide **3**) up to 99.5:0.5 er (99% ee).

Due to the potential complications highlighted throughout this work, we recommend that hydrogen-bonding-based CSA protocols used to determine er values of scalemic analytes should be repeated using both CSA enantiomers to identify whether er-dependent variations in diagnostic chemical shifts need to be accounted for, particularly in those cases where single resonances are observed (risk of peak overlap) or absolute configuration is being determined (possible signal crossover). We also re-emphasize the importance of carefully reporting analyte concentrations and CSA stoichiometries in experimental protocols to ensure that optimal CSA conditions can be reproduced effectively, and any risk of misassignment minimized.

## Supporting Information Available

Experimental procedures, synthetic details, characterization, NMR spectra, and X-ray crystal structures (PDF)
