# Supplementary material for: BINOL as a Chiral Solvating Agent for Sulfiniminoboronic Acids
Source: Anal Chem. 2023 Nov 6;95(46):16801–9. doi: 10.1021/acs.analchem.3c01613 (PMC10666087; doi:10.1021/acs.analchem.3c01613)
Supplement: Supplementary file 1 — ac3c01613_si_001.pdf [file ac3c01613_si_001.pdf]

SUPPORTING INFORMATION

BINOL as a chiral solvating agent for sulfiniminoboronic acids

Robin R. Groleau[a], Robert S. L. Chapman[a], John P. Lowe[a], Catherine L. Lyall[a] ,Gabriele Kociok-Köhn[a], Tony D. James[a,b], and Steven D. Bull.[a,c]*

[a] Department of Chemistry, University of Bath, Claverton Down, Bath, BA2 7AY, U.K.

[b] School of Chemistry and Chemical Engineering, Henan Normal University, Xinxiang 453007, China.

[c] School of Chemistry, University of Leicester, Leicester, LE1 7RH, U.K.

E-mail: *sdb45@leicester.ac.uk*

Contents

1 Previously reported SIBE results 2

2 General experimental details 4

3 ^{1}H NMR data of SIBA-BINOL assemblies 5

4 ^{15}N NMR studies of N-B coordination in IBE, SIBE and SIBA assemblies 14

5 Reactions of (1*S*,2*S*,3*R*,5*S*)-pinanediol and BINOL with selected boronic acids. 15

6 Literature reports of BINOL boronates containing planar *sp*^{2}-boron atoms. 16

7 Synthesis and characterisation of compounds 17

8 NMR spectra of pure compounds 23

9 NMR spectra of IBEs, SIBE and SIBA assemblies 37

10 X-ray crystallography 50

11 References 71

1 Previously reported SIBE results

Tables S1 and S2 show $\Delta\delta_H$ values previously published from our SIBE assembly CDA protocol that are relevant to this study.[1]

Table S1: Chemical shift differences ($\Delta\delta_H$) ^{1}H NMR (500 MHz, dried $CDCl_3$, 100 mM) spectra of diastereomeric iminoboronate complexes of Ellman's sulfinamide **3** ($75_{(S)}$:$25_{(R)}$ *er*), 2-FPBA and a range of enantiopure diols.[1]

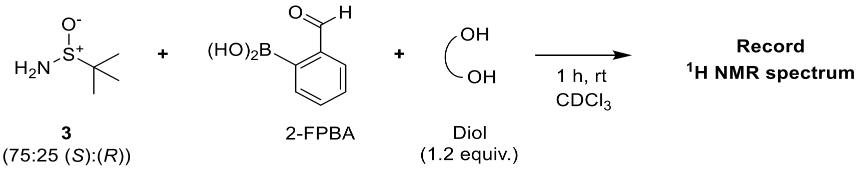

Entry[a]	Diol	$\Delta\delta_H$ (ppm)[b,c]
1[d]	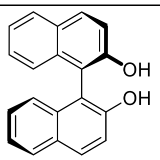	−0.012
2	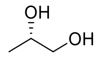	+0.006
3	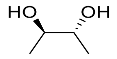	+0.027
4	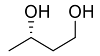	+0.010
5	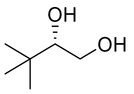	+0.014
6[e]	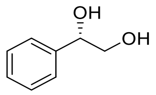	+0.037
7[e]	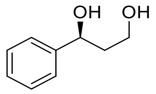	+0.047
8[e]	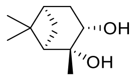	−0.085

[a] One-pot complexation reactions carried out on 0.1 mmol of sulfinamide **3** ($75_{(S)}$:$25_{(R)}$ *er*) at 0.1 M concentration. [b] $\Delta\delta_H$ is the difference in chemical shifts of the pairs of the diastereomeric imine protons in the ^{1}H NMR spectra of the complexation reaction of each chiral diol. [c] A negative value for $\Delta\delta_H$ indicates that the imine proton resonance of the homochiral imine peak was most deshielded. [d] BINOL does not form SIBE products (see manuscript). [e] Full baseline resolution observed for the imine resonances of their respective diastereomeric iminoboronate esters.

Table S2: Three-component stepwise assembly of diastereomeric SIBE complexes of 2-FPBA , (1*R*,2*R*,3*S*,5*R*)-pinanediol, and racemic sulfinamides.[1]

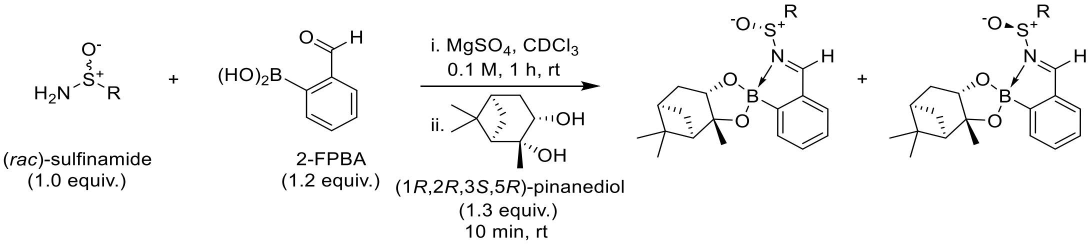

Entry	Sulfinamide	Conversion (%)	$\Delta\delta_H$ (ppm)[a]
1	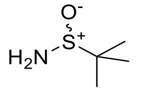	99	0.085
2	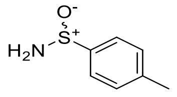	62	0.069
3	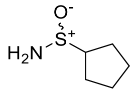	98	0.061
4	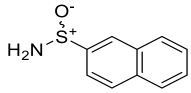	97	0.077
5	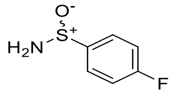	63	0.057
6	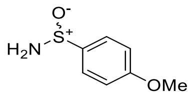	69	0.070
7	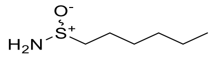	80	0.062
8	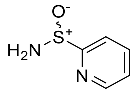	55	0.061

[a] δ_H is the difference in chemical shifts of the imine protons of the pairs of diastereomeric SIBE complexes for each chiral diol.

2 General experimental details

Reagents and solvents were obtained from commercial suppliers and used without further purification. Reactions were performed without air exclusion or drying, at room temperature, and with magnetic stirring, unless otherwise stated. Anhydrous $MgSO_4$ or Na_2SO_4 were used as drying agents for organic solvents. Thin layer chromatography (TLC) was carried out on Macherey-Nagel aluminium-backed plates that were precoated with silica. Compounds were visualised by either quenching of UV fluorescence at 254 nm, or by dip-staining ($KMnO_4$, PMA, curcumin,[2] I_2) followed by gentle heating. Purification by flash column chromatography was performed using high-purity grade silica gel (60 Å pore size, 40-75 µm particle size). PE refers to Petroleum ether 40-60 °C.

Capillary melting points were determined using a Stuart digital SMP10 melting point apparatus and are reported uncorrected to the nearest °C. Optical rotations were measured using an Optical Activity Ltd AA-10 Series Automatic Polarimeter, with a path length of 1 dm, and with concentration (*c*) quoted in g/100 mL.

Nuclear Magnetic Resonance (NMR) spectroscopy experiments were performed in deuterated solvents at 298 K (unless otherwise stated) on either a Bruker 500 MHz spectrometer or an Agilent ProPulse 500 MHz spectrometer. ^{1}H, ^{13}C, ^{11}B, and ^{19}F NMR chemical shifts (δ) are quoted in parts per million (ppm) and are referenced to either the residual solvent peak or tetramethylsilane (TMS) when possible.[3] ^{11}B NMR spectra were referenced directly, using the lock signal, to external $BF_3.Et_2O$ (0 ppm). Coupling constants (J) are quoted in Hz. In those cases where ^{13}C signals could not be observed by 1D NMR spectroscopy due to low solubility, adjacent quadrupolar nuclei, or lack of adjacent ^{1}H nuclei, then chemical shifts were measured indirectly from 2D ^{1}H-^{13}C HMBC experiments.[1]

^{15}N NMR chemical shifts were measure indirectly from 2D ^{1}H-^{15}N HMBC spectra using a Bruker Avance 500 MHz spectrometer.[4] ^{15}N NMR experiments were carried out at 50 mM sample concentrations. ^{15}N NMR spectroscopy was carried out in $CDCl_3$ containing 50 mM nitromethane internal standard. The $MeNO_2$ ^{15}N resonance was used as the reference for ^{15}N chemical shifts, setting $\delta_N(MeNO_2) = 0.00$ ppm.[5]

Infrared (IR) spectra were recorded using a PerkinElmer Spectrum 100 FTIR spectrometer fitted with a Universal ATR FTIR accessory, with samples run neat and selected absorbances quoted as v in cm^{-1}.

High resolution mass spectrometry (HRMS) results were acquired on an externally calibrated Bruker Daltonics maXis HD™ UHR-TOF mass spectrometer coupled to an electrospray source (ESI-TOF), or an Agilent QTOF 6545 with Jetstream ESI. In most cases molecular ions were detected either in positive mode as their protonated, sodiated, or ammonium adduct forms, or in negative mode as deprotonated or acetate adduct species.

3 ^{1}H NMR data of SIBA-BINOL assemblies

3.1 BINOL loading and concentration screening experiments

Table S3: Tabulated data for **Figure 2a**. Chemical shifts and $\Delta\delta_H$ values of ^{1}H NMR (500 MHz, $CDCl_3$, 100 mM) imine signals of heterochiral and homochiral mixtures of (*rac*)-**4** and (*R*)-BINOL at different CSA loadings.[a]

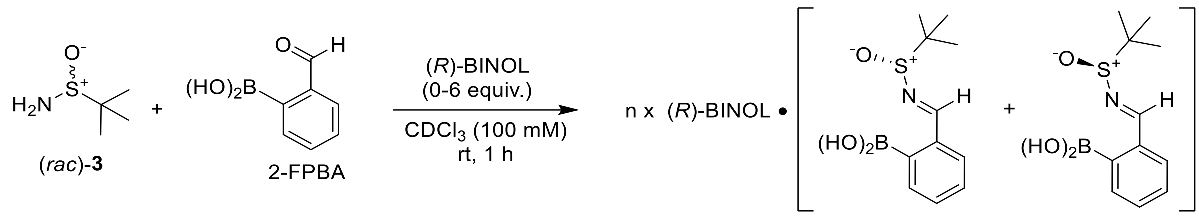

n x (*R*)-BINOL • [(R_S)-**4**+(S_S)-**4**]

Entry	(*R*)-BINOL loading (%)	Imine δ_H (ppm)[b]		$\Delta\delta_H$ (ppm)[d]
		(S_S)-**4**	(R_S)-**4**	
1	0	9.033[c]		--
2	10	9.156[c]		--
3	20	9.155[c]		--
4	40	9.129[c]		--
5	60	9.117	9.123	−0.006
6	100	9.075	9.085	−0.010
7	150	9.015	9.030	−0.015
8[e]	300	9.010	9.028	−0.018
9[e]	450	8.968	8.992	−0.024
10[e]	600	8.974	8.997	−0.023

[a] Data extracted from ^{1}H NMR spectra shown in **Figure 2a** using MestReNova software. [b] Chemical shifts of the imine protons of the respective SIBA **4** complexes. [c] Imine signals coalesced into a singlet, so chemical shift differences could not be reported. [d] A negative value for $\Delta\delta_H$ indicates that the imine proton resonance of the homochiral SIBA·BINOL system is most deshielded. [e] Concentration of (*R*)-BINOL reached saturation, with samples producing poor quality spectra that required filtration, meaning that the actual concentration of BINOL is unknown.

Table S4: Tabulated data for **Figure 2b**. Chemical shifts and $\Delta\delta_H$ values of ^{1}H NMR (500 MHz, $CDCl_3$) imine signals of heterochiral and homochiral mixtures of (*rac*)-**4** and (*R*)-BINOL (1.0 equiv.) at different concentrations.[a]

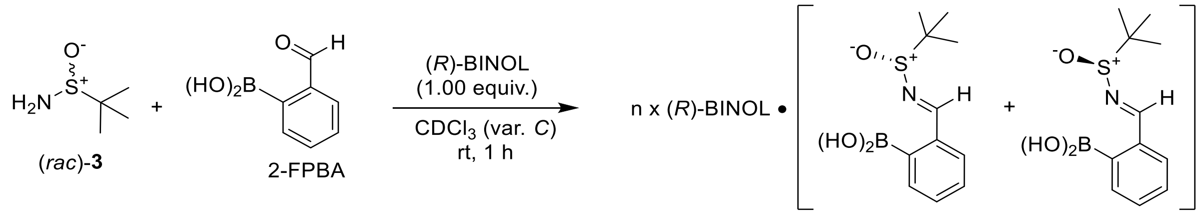

n x (*R*)-BINOL • [(R_S)-**4**+(S_S)-**4**]

Entry	Concentration (mM)	Imine δ_H (ppm)[b]		$\Delta\delta_H$ (ppm)[d]
		(S_S)-**4**	(R_S)-**4**	
1	100	9.051	9.061	−0.010
2	50	9.034	9.040	−0.006
3	25	8.973[c]		--
4	1.5	8.883[c]		--
5	5.0	8.809[c]		--
6	2.5	8.728[c]		--
7	1.0	8.691[c]		--

[a] Data extracted from ^{1}H NMR spectra shown in **Figure 2b** using MestReNova software. [b] Chemical shift of the imine protons of the respective SIBA **5** complexes. [c] Imine signals coalesced into a singlet, so chemical shift differences could not be reported. [d] A negative value for $\Delta\delta_H$ indicates that the imine proton resonance of the homochiral SIBA·BINOL system was most deshielded.

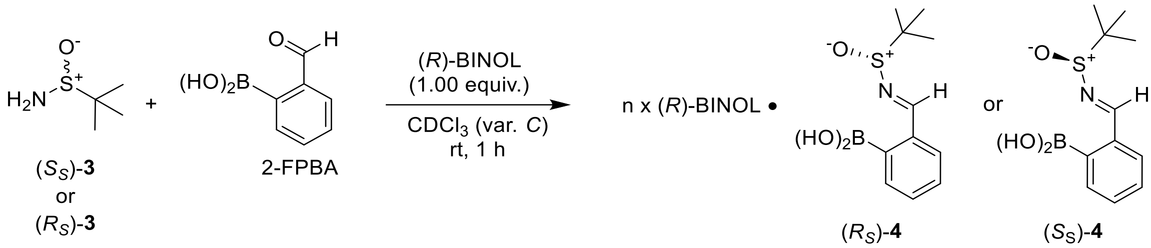

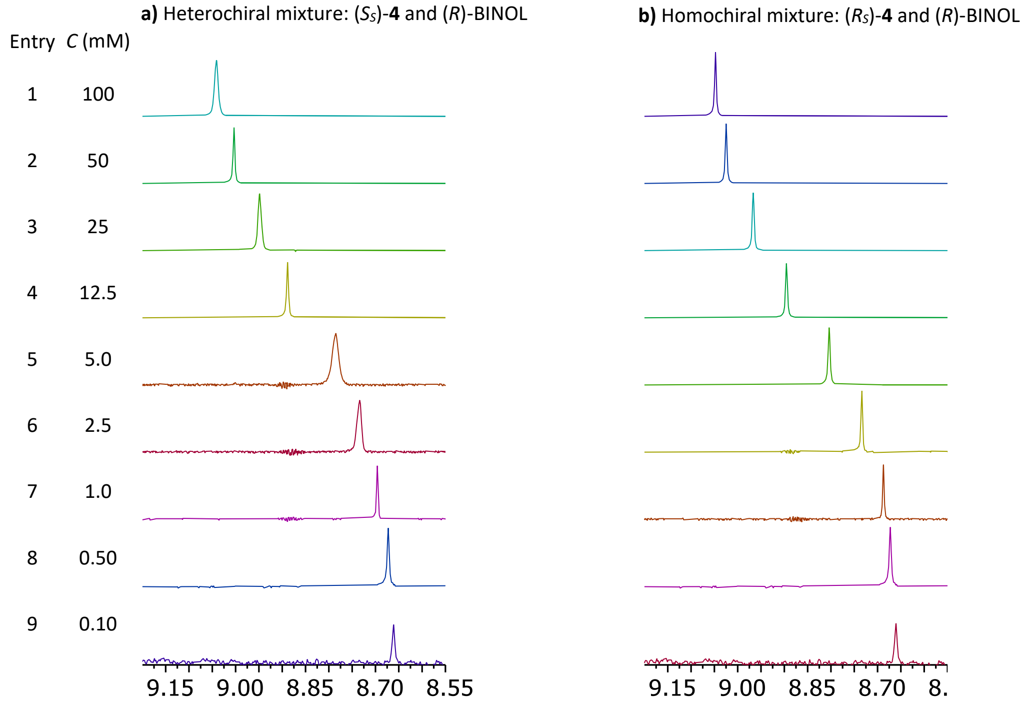

Figure S1 :Expanded imine regions of the ^{1}H NMR spectra (500 MHz, $CDCl_3$) of equimolar mixtures of (*R*)-BINOL, 2-FPBA and enantiopure Ellman's sulfinamide **3** to produce: (a) heterochiral mixtures from (S_S)-**3**; or (b) homochiral mixtures from (R_S)-**3**. Spectra acquired at decreasing sample concentrations from 100 mM (top) to 0.10 mM (bottom). Chemical shifts referenced to TMS as an internal standard (~6 mM in original 100 mM stock solution).[3]

Table S5: Tabulated data for Figure S1. Chemical shifts and $\Delta\delta_H$ values of ^{1}H NMR imine signals of heterochiral and homochiral mixtures of (S_S)-**4** or (R_S)-**4** and (*R*)-BINOL at different concentrations.[a]

Entry	Concentration (mM)	*Imine δ_H (ppm)*[b]		$\Delta\delta_H$ (ppm)c[d]
		(S_S)-**4**	(R_S)-**4**	
1	100	9.041	9.048	−0.007
2	50	9.003	9.025	−0.022
3	25	8.948	8.967	−0.019
4	12.5	8.889	8.896	−0.007
5	5.0	8.786	8.804	−0.018
6	2.5	8.734[e]	8.734[e]	0.000
7	1.0	8.696	8.687	+0.009
8	0.50	8.673[e]	8.673[e]	0.000
9	0.10	8.667	8.661	+0.006

[a] Data extracted from ^{1}H NMR spectra shown in Figure S1 using MestReNova software. [b] Chemical shift of the imine protons of the respective SIBA **5** sample. [c] 'Baseline' chemical shift differences between enantiopure mixtures at the same concentration. [d] A negative value for $\Delta\delta_H$ indicates that the imine proton resonance of the homochiral SIBA·BINOL system was most deshielded. [e] Imine signals displayed same chemical shift, so chemical shift differences could not be measured.

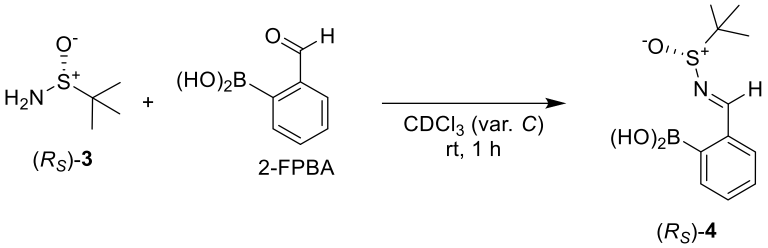

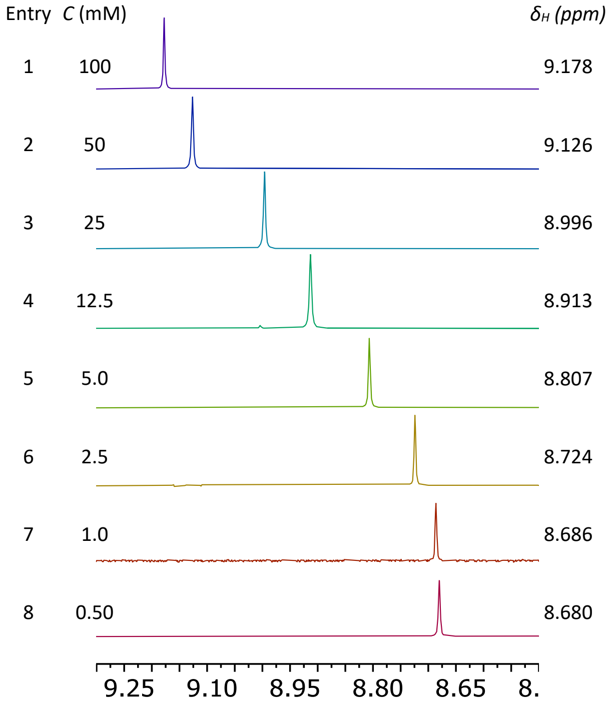

Figure S2 :Expanded imine regions of the ^{1}H NMR spectra (500 MHz, $CDCl_3$) of equimolar mixtures of 2-FPBA and enantiopure Ellman's sulfinamide **3** to produce SIBA (R_S)-**4**. Spectra acquired at decreasing sample concentrations from 100 mM (top) to 0.10 mM (bottom). Chemical shifts referenced to TMS as an internal standard (~6 mM in original 100 mM stock solution).

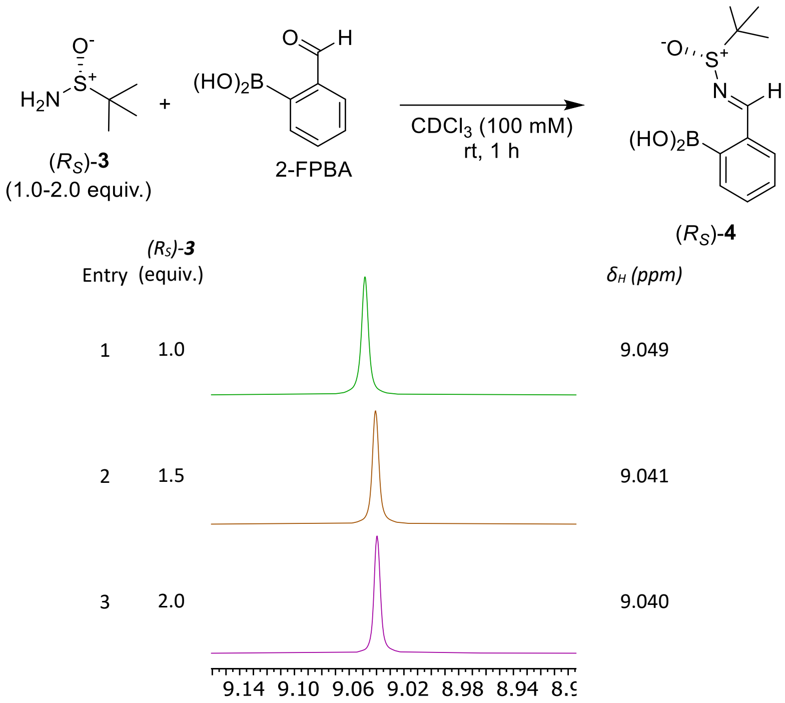

Figure S3 :Expanded imine regions of the ^{1}H NMR spectra (500 MHz, $CDCl_3$) of mixtures of 2-FPBA and enantiopure Ellman's sulfinamide (R_S)-**3** to produce SIBA (R_S)-**4**. Chemical shifts referenced to TMS as an internal standard (~6 mM in original 100 mM stock solution).

3.2 Enantiomeric ratio screening experiments

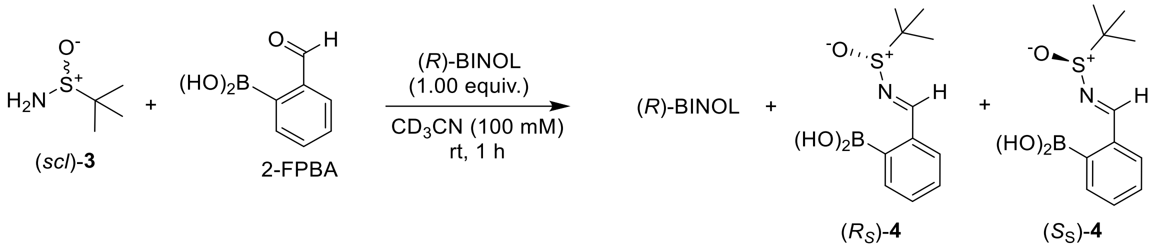

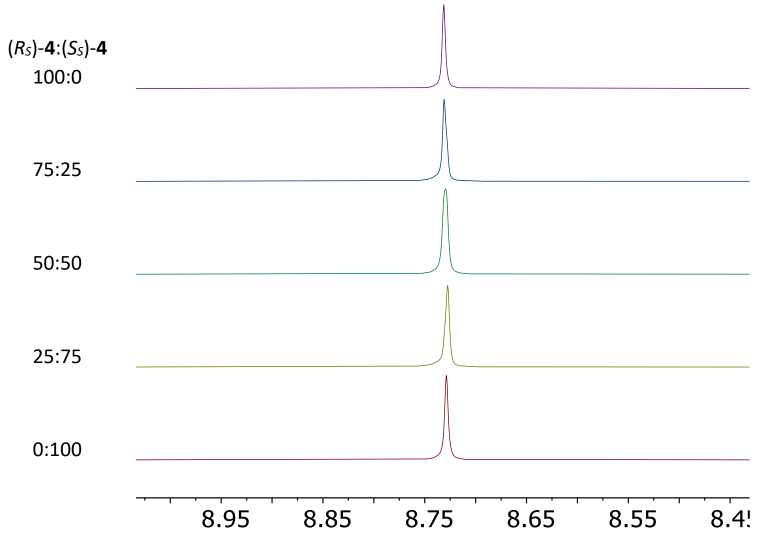

Figure S4: Expanded imine regions of the ^{1}H NMR (500 MHz, CD_3CN, 100 mM) spectra of equimolar mixtures of (*R*)-BINOL (1.00 equiv.), 2-FPBA and enantiopure Ellman's sulfinamide **3** ($100_{(R)}:0_{(S)} \rightarrow 0_{(S)}:100_{(R)}$ er). Chemical shifts referenced to TMS as an internal standard (~6 mM in original 100 mM stock solution).

Table S6: Tabulated data for **Figure 5a**. ^{1}H NMR δ_H and $\Delta\delta_H$ values for the imine resonances of (S_S)-**4** and (R_S)-**4** (varying er) in the presence of 1.50 equiv. (R)-BINOL.[a]

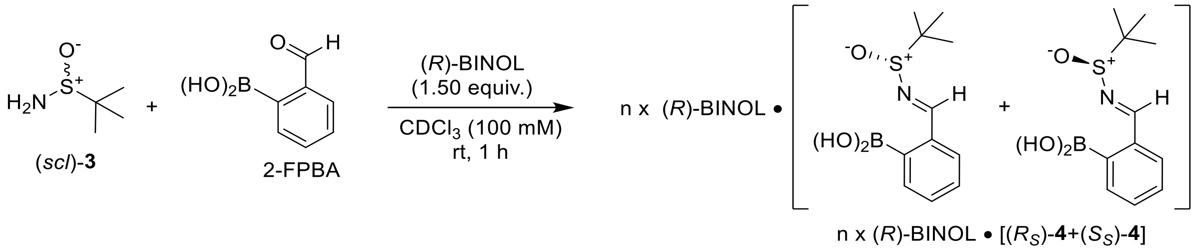

Entry	(S_S)-**3**:(R_S)-**3** (er)	*Imine δH (ppm)*[b]		$\Delta\delta_H$ (ppm)[c]
		(S_S)-**4**	(R_S)-**4**	
1	100:0	8.999	N/A	N/A
2	90:10	9.009	9.045	−0.036
3	80:20	9.009	9.039	−0.030
4	70:30	9.013	9.038	−0.025
5	60:40	9.017	9.035	−0.018
6	50:50	9.015	9.030	−0.015
7	40:60	9.031	9.021	−0.010
8	30:70	9.025	9.020	−0.005
9[d]	20:80	9.020	9.020	--
10[d]	10:90	9.011	9.011	--
11	0:100	N/A	9.000	N/A

[a] Data extracted from ^{1}H NMR spectra shown in **Figure 5a** using MestReNova software. [b] Chemical shift of the imine protons of the respective SIBA complex. [c] A negative value for $\Delta\delta_H$ indicates that the imine proton resonance of the homochiral SIBA·BINOL system was most deshielded. [d] Signals coalesced, so no $\Delta\delta_H$ value reported.

Table S7: Tabulated data for **Figure 5b**. ^{1}H NMR δ_H and $\Delta\delta_H$ values for the imine resonances of (S_S)-**4** and (R_S)-**4** (varying er) in the presence of 1.50 equiv. (S)-BINOL.[a]

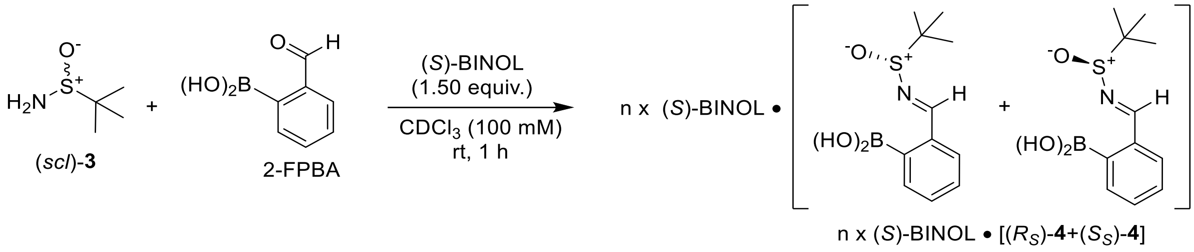

Entry	(S_S)-**3**:(R_S)-**3** (er)	*Imine δH (ppm)*[b]		$\Delta\delta_H$ (ppm)[c]
		(S_S)-**4**	(R_S)-**4**	
1	100:0	9.020	N/A	N/A
2	90:10	9.023	9.023	--
3	80:20	9.025	9.025	--
4	70:30	9.025	9.019	−0.006
5	60:40	9.028	9.018	−0.010
6	50:50	9.030	9.015	−0.015
7	40:60	9.036	9.016	−0.020
8	30:70	9.042	9.018	−0.024
9[d]	20:80	9.038	9.008	−0.030
10[d]	10:90	9.037	9.002	−0.035
11	0:100	N/A	8.999	N/A

[a] Data extracted from ^{1}H NMR spectra shown in **Figure 5b** using MestReNova software. [b] Chemical shift of the imine protons of the respective SIBA complex. [c] A negative value for $\Delta\delta_H$ indicates that the imine proton resonance of the homochiral SIBA·BINOL system was most deshielded. [d] Signals coalesced, so no $\Delta\delta_H$ value reported.

Table S8: Tabulated data for **Figure 5c**. ^{1}H NMR δ_H and $\Delta\delta_H$ values for the imine resonances of (S_S)-**4** and (R_S)-**4** (varying er) in the presence of 1.00 equiv. (*R*)-BINOL.[a]

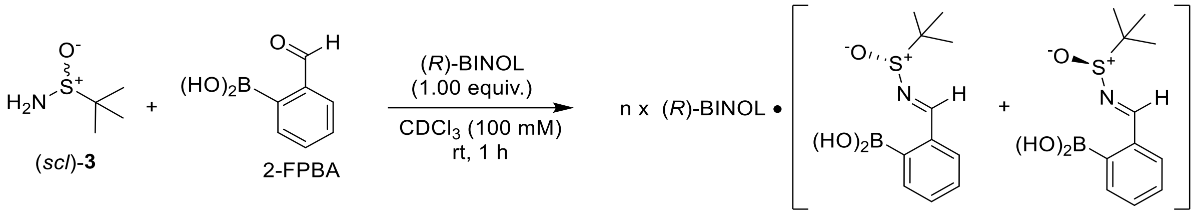

Entry	(S_S)-**3**:(R_S)-**3** (er)	Imine δ_H (ppm)[b]		$\Delta\delta_H$ (ppm)[c]
		(S_S)-**4**	(R_S)-**4**	
1	100:0	9.041	N/A	N/A
2	90:10	9.044	9.074	−0.030
3	80:20	9.045	9.071	−0.026
4	70:30	9.046	9.071	−0.025
5	60:40	9.046	9.063	−0.017
6	50:50	9.051	9.061	−0.010
7	40:60	9.051	9.057	−0.006
8[d]	30:70	9.053	9.053	--
9	20:80	9.056	9.052	+0.004
10	10:90	9.058	9.051	+0.007
11	0:100	N/A	9.048	N/A

[a] Data extracted from ^{1}H NMR spectra shown in **Figure 5c** using MestReNova software. [b] Chemical shift of the imine protons of the respective SIBA complex. [c] A negative value for $\Delta\delta_H$ indicates that the imine proton resonance of the homochiral SIBA·BINOL system was most deshielded. [d] Signals coalesced, so no $\Delta\delta_H$ value reported.

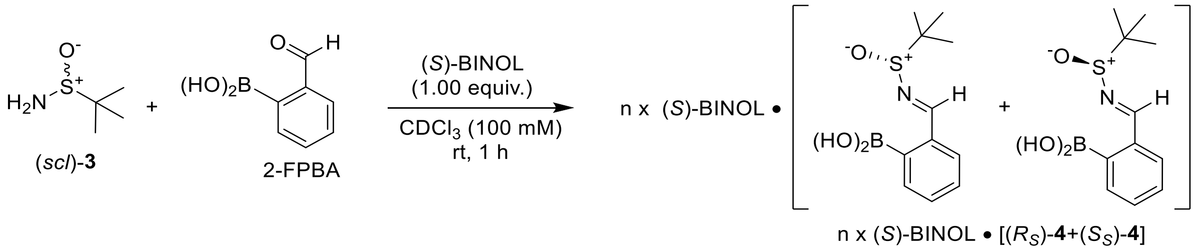

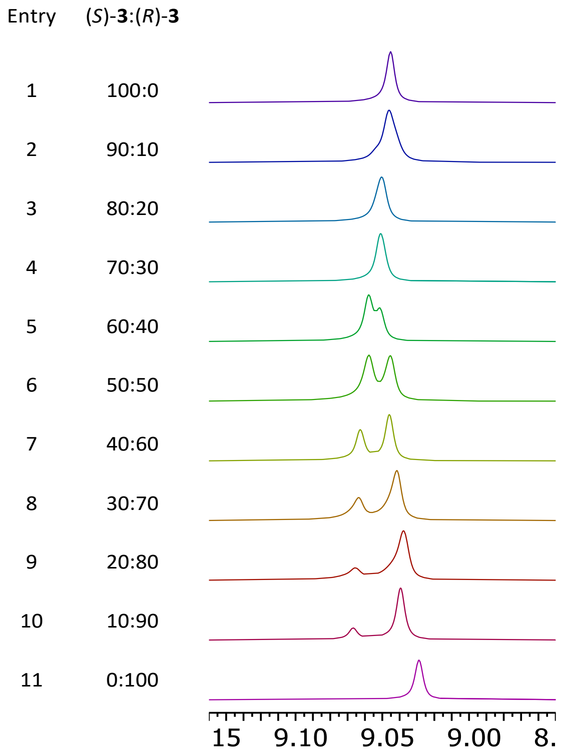

Figure S5**:** Expanded imine regions of the ^{1}H NMR spectra (500 MHz, $CDCl_3$, 100 mM) of SIBA **4** of varying er (ranging from 100% (*S*)-**4**→100% (*R*)-**4** in 10% er increments from top to bottom) in the presence of 1.00 equiv. (*R*)-BINOL. Chemical shifts referenced to TMS as an internal standard (~6 mM).

Table S9: Tabulated data for Figure S5. ^{1}H NMR δ_H and $\Delta\delta_H$ values for the imine resonances of (S_S)-**4** and (R_S)-**4** (varying er) in the presence of 1.00 equiv. (*S*)-BINOL.[a]

Entry	(S_S)-**3**:(R_S)-**3** (er)	*Imine δ_H (ppm)*[c]		$\Delta\delta_H$ (ppm)[d]
		(S_S)-**4**	(R_S)-**4**	
1	100:0	9.045	N/A	N/A
2	90:10	9.046	9.054	+0.008
3[b]	80:20	9.051	9.051	--
4[b]	70:30	9.051	9.051	--
5	60:40	9.058	9.051	−0.007
6	50:50	9.058	9.045	−0.013
7	40:60	9.063	9.046	−0.017
8	30:70	9.064	9.042	−0.023
9	20:80	9.066	9.038	−0.028
10	10:90	9.067	9.039	−0.028
11	0:100	N/A	9.029	N/A

[a] Data extracted from ^{1}H NMR spectra shown in Figure S4 using MestReNova software. [b] Chemical shift of the imine protons of the respective SIBA complex. [d] A negative value for $\Delta\delta_H$ indicates that the imine proton resonance of the homochiral SIBA·BINOL system was most deshielded. [e] Signals coalesced, so no $\Delta\delta_H$ value reported.

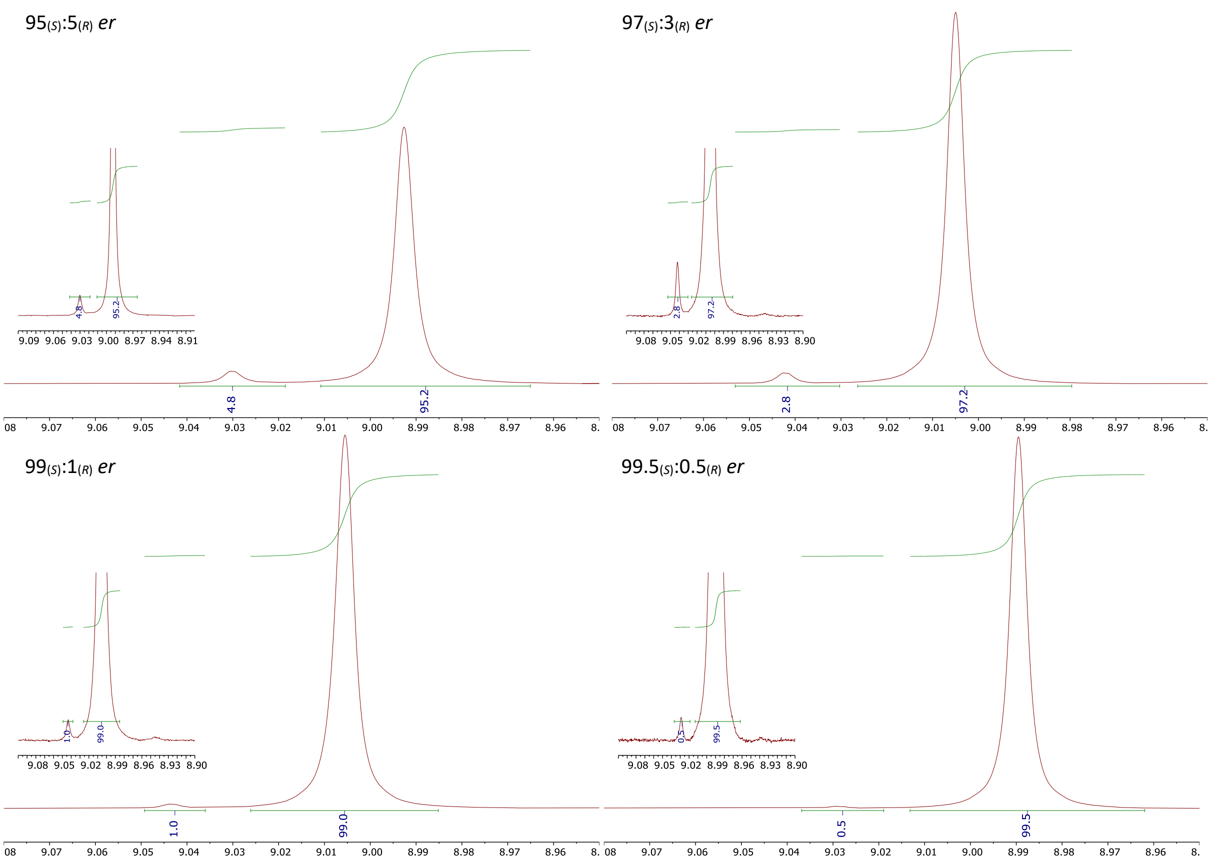

Figure S6**:** Detection limits. Expanded imine regions of the ^{1}H NMR spectra (500 MHz, $CDCl_3$, 100 mM) of SIBA **4** of high enantiopurity ($95_{(S)}$:$5_{(R)}$-$99.5_{(S)}$:$0.5_{(R)}$ er) in the presence of 1.50 equiv. (*R*)-BINOL. Chemical shifts referenced to TMS as an internal standard (~6 mM). Measured integrals are well within accepted margins of error. Insets show enhanced view of minor resonances.

4 ^{15}N NMR studies of N-B coordination in IBE, SIBE and SIBA assemblies

Table S10: ^{1}H, ^{11}B and ^{15}N NMR chemical shift values of IBEs, SIBAs and SIBEs, as a method of determining whether intramolecular N⟶B coordination is present.

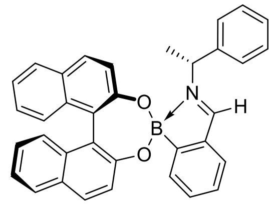

(α-*R*,*R*)-**2a**

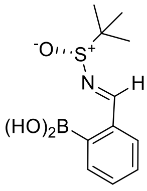

(*R*$_S$)-**4**

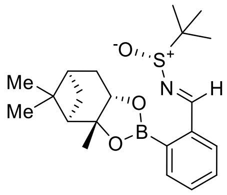

(*R*$_S$,*R*)-**5a**

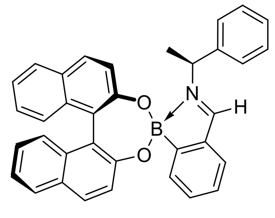

(α-*S*,*R*)-**2b**

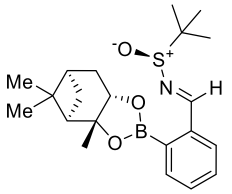

(*S*$_S$,*R*)-**5b**

Entry	Compound	δ_H (ppm)^a	δ_B (ppm)	δ_N (ppm)^b	N⟶B bond
1	(α-*R*,*R*)-**2a**	8.08	12.7	−118.1	✓
2	(α-*S*,*R*)-**2b**	8.25	12.5	−116.7	✓
3	(*R*$_S$)-**4**	9.12	28.6	−67.0	×
4	(*R*$_S$,*R*)-**5a**	9.36	30.5	−53.3	×
5	(*S*$_S$,*R*)-**5b**	9.27	31.2	−51.7	×
6	(*R*$_S$)-**4** + (*R*)-BINOL (1.00 eq.)	9.05	28.7	−68.0	×
7[1]	(*S*$_S$)-**4** + (*R*)-BINOL (1.00 eq.)	9.04	29.0	−67.0	×
8[1]	(*R*$_S$)-**4** + (*R*)-BINOL (1.50 eq.)	9.02	28.8	−68.5	×

^{a} Chemical shifts of imine proton resonances (N=C*H*). ^{b} ^{15}N NMR spectra recorded at 50 mM in CDCl$_3$. ^{15}N NMR chemical shifts δ_N referenced to CH_3NO_2 internal standard (50 mM, $\delta_{H/N}$ 4.33/0.00 ppm).

Table S11: Reference/benchmarking ^{1}H, ^{11}B and ^{15}N NMR chemical shift values of compounds containing non-coordinated imine and sulfinimine groups.

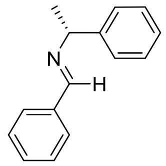

(α-*R*)-**6**

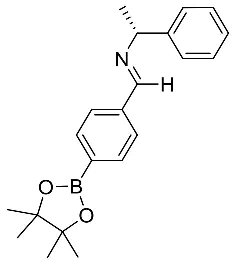

(α-*R*)-**7**

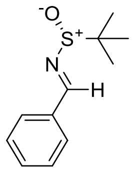

(*R*$_S$)-**8**

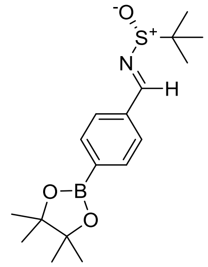

(*R*$_S$)-**9**

Entry	Compound	δ_H (ppm)^{a,b}	δ_B (ppm)	δ_N (ppm)^c	N⟶B bond
1	(α-*R*)-**6**	8.38	-	−35.1	N/A
2	(α-*R*)-**7**	8.38	30.4	−31.6	×
3	(*R*$_S$)-**8**	8.60	-	−55.1	N/A
4	(*R*$_S$)-**9**	8.60	30.4	−51.7	×

^{a} Chemical shifts of imine proton resonances (N=C*H*). ^{b} ^{1}H NMR spectra recorded at 100 mM concentration. ^{b} ^{15}N NMR spectra recorded at 50 mM in CDCl$_3$. ^{15}N NMR chemical shifts δ_N referenced to CH_3NO_2 internal standard (50 mM, $\delta_{H/N}$ 4.33/0.00 ppm).

5 Reactions of (1*S*,2*S*,3*R*,5*S*)-pinanediol and BINOL with selected boronic acids.

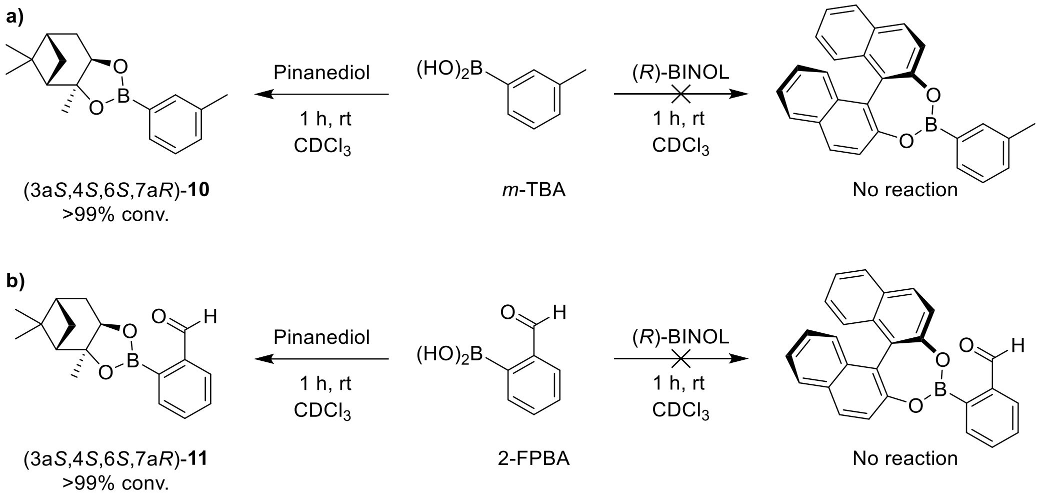

Scheme S1**:** Boronate ester complexation reactions between (1*S*,2*S*,3*R*,5*S*)-pinanediol or (*R*)-BINOL and either: (a) *m*-tolyl boronic acid (*m*-TBA); or (b) 2-FPBA.

6 Literature reports of BINOL boronates containing planar *sp²*-boron atoms.

Kaufmann *et al.*, 2002:[6]

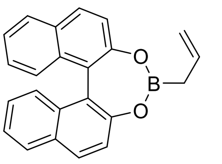

δ_B = 28.2 ppm

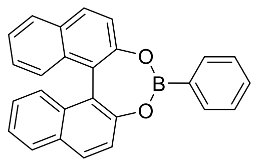

δ_B = 26.4 ppm

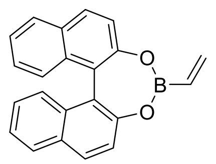

δ_B = 24.5 ppm

Chong *et al.* (formed *in situ*):

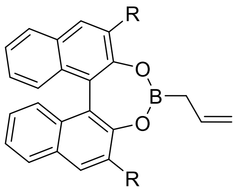

8 examples[7]
no ^{11}B NMR data

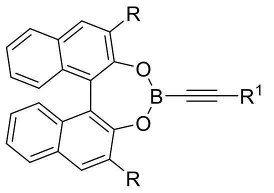

>10 examples[8,9]
no ^{11}B NMR data

Ishihara *et al.* (formed *in situ*):

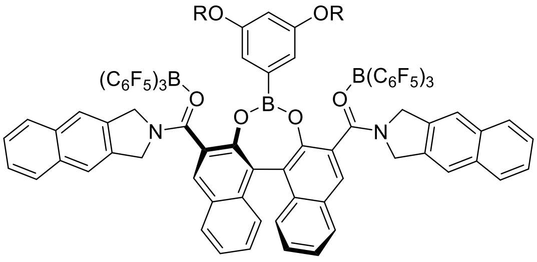

8 examples[10]
no ^{11}B NMR data

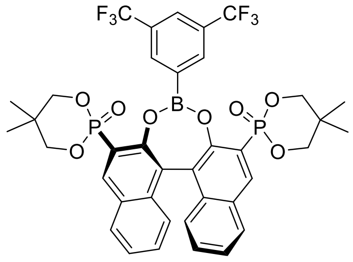

no ^{11}B NMR data[11]

Figure S7: Selected examples of prior reports of planar *sp²*-B BINOL boronate esters with ^{11}B chemical shifts indicating some *sp³*-boron character or no reported ^{11}B NMR spectroscopic data.[6–11]

7 Synthesis and characterisation of compounds

7.1 Synthesis of simple boronate esters

(3a*S*,4*S*,6*R*,7a*S*)-3a,5,5-Trimethyl-2-(*m*-tolyl)hexahydro-4,6-methanobenzo[*d*][1,3,2]dioxaborole (3a*S*,4*S*,6*R*,7a*S*)-10.

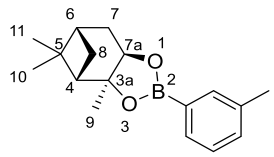

(1*S*,2*S*,3*R*,5*S*)-Pinanediol (17 mg, 0.10 mmol, 1.0 equiv.) was added to a stirred suspension of *m*-tolyl boronic acid (14 mg, 0.1 mmol, 1.0 equiv.) in $CDCl_3$ (1.0 mL). After 30 min, the reaction was diluted with *n*-hexane (1.0 mL) and passed through a silica plug. The plug was washed with 1:1 DCM/*n*-hexane until no more product eluted and the solvent was removed *in vacuo* to afford the title compound (3a*S*,4*S*,6*R*,7a*S*)-**10** (24 mg, 0.089 mmol) as a clear oil in 89% yield.

R_f = 0.51 (SiO_2, 1:1 DCM/*n*-hexane); $[\alpha]_D^{19}$= +16 (*c* 1.0, $CHCl_3$); IR (neat): 2917, 2870, 2104,1607, 1583, 1418, 1399, 1346, 1359, 1282, 1236, 1206, 1122, 1079, 1028, 706, 664 cm^{-1}; ^{1}H NMR (500 MHz, $CDCl_3$) δ_H 7.65 (bs, 1H, BCC*H*CCH$_3$), 7.65-7.60 (m, 1H, Ar*H*), 7.31-7.27 (m, 2H, Ar*H*), 4.46 (dd, 1H, *J* = 8.8, 1.9, H-7a), 247-2.38 (m, 1H, H-7), 2.37 (s, 3H, ArC*H$_3$*), 2.28-2.20 (m, 1H, H-8), 2.16 (dd, 1H, *J* = 6.1, 5.0, H-4), 2.02-1.92 (m, 2H, H-6+H-7), 1.49 (s, 3H, H-9), 1.32 (s, 3H, H-10/11), 1.23 (d, 1H, *J* = 10.7, H-8), 0.90 (s, 3H, H-10/11); $^{13}C\{^1H\}$ NMR (126 MHz, $CDCl_3$) δ_C 137.3, 135.5, 132.1, 131.9, 127.9, 86.3, 78.4, 51.6, 39.7, 38.4, 35.8, 28.9, 27.3, 26.7, 24.2, 21.4; ^{11}B NMR (375.5 MHz, $CDCl_3$) δ_B 30.3; HRMS (ESI+): Calculated for $[M+Na]^+$ $C_{17}H_{23}BO_2Na^+$: 293.1683; Found: 293.1743.

2-((3a*S*,4*S*,6*S*,7a*R*)-3a,5,5-Trimethylhexahydro-4,6-methanobenzo[*d*][1,3,2]dioxaborol-2-yl)benzaldehyde (3a*S*,4*S*,6*S*,7a*R*)-11.

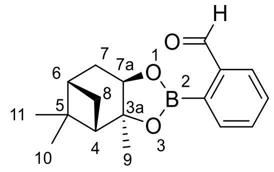

(1*S*,2*S*,3*R*,5*S*)-Pinanediol (85 mg, 0.50 mmol, 1.0 equiv.) was added to a stirred suspension of 2-FPBA (83 mg, 0.55 mmol, 1.1 equiv.) in $CHCl_3$ (5.0 mL). After 15 min, the reaction was diluted with an equivalent amount of CH_2Cl_2 and passed through a silica plug. The plug was washed with CH_2Cl_2 until no more product eluted and the solvent was removed *in vacuo* to afford the title compound (3a*S*,4*S*,6*S*,7a*R*)-**11** (110 mg, 0.39 mmol) as a clear oil in 70% yield. Characterisation data were consistent with previous literature reports.[1]

R_f = 0.21 (SiO_2, 1:1 DCM/*n*-hexane); $[\alpha]_D^{23}$= +18 (*c* 1.0, $CHCl_3$); ^{1}H NMR (500 MHz, $CDCl_3$) δ_H 10.55 (s, 1H, C*H*O), 7.98-7.95 (m, 1H, Ar*H*), 7.90-7.86 (m, 1H,Ar*H*), 7.62-7.53 (m, 2H, Ar*H*), 4.52 (dd, 1H, *J* = 8.8,

1.9, H-7a), 2.48-2.39 (m, 1H, H-7), 2.32-2.23 (m, 1H, H-8), 2.16 (dd, 1H, *J* = 6.0, 4.9, H-4), 2.04-1.94 (m, 2H, H-6 + H-7), 1.53 (s, 3H, H-9), 1.33 (d, 1H, *J* = 10.8, H-8), 1.32 (s, 3H, H-10/11), 0.90 (s, 3H, H-10/11); $^{13}C\{^1H\}$ NMR (126 MHz, $CDCl_3$) δ_C 194.7, 141.4, 135.7, 133.1, 132.2 (deduced from HMBC), 130.8, 128.0, 86.9, 78.6, 51.5, 39.7, 38.4, 35.5, 28.7, 27.2, 26.6, 24.2; ^{11}B NMR (375.5 MHz, $CDCl_3$) δ_B 30.7.

4-(4,4,5,5-tetramethyl-1,3-dioxolan-2-yl)benzaldehyde 4-FPBPin.

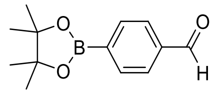

4-FPBA (2.70 g, 18.0 mmol, 1.20 mmol), pinacol (1.77 g, 15.0 mmol, 1.00 equiv.) and anhydrous $MgSO_4$ (2.00 g) were suspended in $CHCl_3$ (18 mL). The reaction was left to stir at room temperature for 16 h, before filtering and concentrating to dryness *in vacuo*. The crude product was purified by silica plug (SiO_2, 1:1 DCM/hexanes) to afford the desired 4-FPBPin (3.303 g, 14.25 mmol) as a white solid in 95% yield. Characterisation data were consistent with previous literature reports.[12]

R_f = 0.18 (SiO_2, 1:1 DCM:*n*-hexane); m.p.: 56-59 °C (lit.[13] 58-59 °C); IR (neat): *v* 2979, 2931, 2849, 2747, 1698, 1606, 1563, 1508, 1461, 1398, 1386, 1357, 1334, 1304, 1272, 1204, 1167, 1138, 1088, 1014, 962, 854, 823, 798, 731, 661 cm^{-1}; ^1H NMR (500 MHz, $CDCl_3$) δ_H 10.05 (s, 1H, C(O)*H*), 7.96 (m, apparent d from AA'XX', *J* = 7.7 Hz, 2H, Ar*H*), 7.86 (m, apparent d from AA'XX', *J* = 7.7 Hz, 2H, Ar*H*), 1.36 (s, 12H, 4 × C*H*$_3$); $^{13}C\{^1H\}$ NMR (126 MHz, $CDCl_3$) δ_C 192.8, 138.3, 135.6 (deduced by HMBC), 135.4, 128.8, 84.5, 25.0; ^{11}B NMR (160 MHz, $CDCl_3$) δ_B 30.4; HRMS (ESI$^+$): Calculated for $[M+NH_4]^+$ $C_{13}H_{21}BO_3N^+$: 250.1611; Found: 250.1646.

7.2 Synthesis of reference imines and sulfinimines

General procedure 1 for the synthesis of imines – adapted from the procedure of Cid *et al*.[14]

The desired amine or sulfinamide (1.0 mmol, 1.0 equiv.), aldehyde (1.0 mmol, 1.0 equiv.), pyrrolidine (0.10 mmol, 8.3 µL, 10 mol%) and activated molecular sieves (4 Å, powdered, 1.00 g) were combined in dichloromethane in a sealed pressure tube. The reaction was stirred at the specifed temperature for the stated amount of time (see individual products), before being filtered and concentrated to dryness under high vacuum to afford the desired imine or sulfinimine without further purification.

(*R*,*E*)-1-phenyl-*N*-(1-phenylethyl)methanimine (α-*R*)-6.

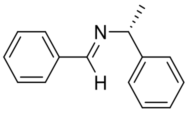

General procedure 1 was followed using (*R*)-α-methylbenzylamine (α-*R*)-**1** (128 µL, 1.00 mmol, 1.00 equiv.) and benzaldehyde (102 µL, 1.00 mmol, 1.00 equiv.), carrying out the reaction for 2 h at room temperature, to afford the title compound (α-*R*)-**6** (195 mg, 0.93 mmol) as a yellow oil in 93% yield. Characterisation data were consistent with previous literature reports.[14,15]

$[\alpha]_D^{29}$= −60 ($CHCl_3$, *c* = 1.0) (lit.[14] $[\alpha]_D^{25}$ = +61.5 for (α-*S*)-**6**, $CHCl_3$, *c* = 1.04); IR (neat): 3061, 3027, 2970, 2925, 2849, 1950, 1644, 1601, 1580, 1492, 1450, 1379, 1292, 1278, 1218, 1200, 1169, 1157, 1118, 1105, 1069, 1025, 1013, 1000, 966, 907, 861, 778, 751, 691 cm^{-1}; ^1H NMR (500 MHz, $CDCl_3$) δ_H 8.38 (s, 1H, N=C*H*), 7.79 (dd, *J* = 6.6, 3.0 Hz, 2H, Ar*H*), 7.46 – 7.38 (m, 5H, Ar*H*), 7.35 (t, *J* = 7.6 Hz, 2H, Ar*H*), 7.27 – 7.22 (m, 1H, Ar*H*), 4.55 (q, *J* = 6.6 Hz, 1H, NC*H*), 1.60 (d, *J* = 6.7 Hz, 30H).; $^{13}C\{^1H\}$ NMR (126 MHz, $CDCl_3$) δ_C 159.6, 145.3, 136.6, 130.7, 128.7, 128.6, 128.4, 127.0, 126.8, 69.9, 25.0; ^{15}N NMR (51 MHz, $CDCl_3$) δ_N –35.1; HRMS (ESI+): Calculated for $[M+H]^+$ $C_{15}H_{16}^+$: 210.1277; Found: 210.1273.

(*R*,*E*)-*N*-(1-phenylethyl)-1-(4-(4,4,5,5-tetramethyl-1,3,2-dioxaborolan-2-yl)phenyl)methanimine (α-*R*)-7.

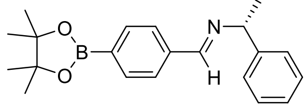

The general procedure 1 was followed using (*R*)-α-methylbenzylamine (α-*R*)-**1** (128 µL, 1.00 mmol, 1.00 equiv.) and 4-FPBPin (232 mg, 1.00 mmol, 1.00 equiv.) to afford the title compound (α-*R*)-**7** (304 mg, 0.91 mmol) as a pink solid in 91% yield.

$[\alpha]_D^{29}$= −73 ($CHCl_3$, *c* = 1.0); m.p.: 73-75 °C; IR (neat): *v* 2979, 2933, 2866, 1635, 1608, 1557, 1511, 1493, 1453, 1392, 1372, 1355, 1326, 1300, 1270, 1213, 1167, 1141, 1113, 1085, 1061, 1031, 1014, 963, 908, 855, 833, 812, 779, 761, 740, 702, 672, 654 cm^{-1}; ^1H NMR (500 MHz, $CDCl_3$) δ_H 8.38 (s, 1H, N=C*H*), 7.87 – 7.82 (m, 2H, Ar*H*), 7.80-7.75 (m, 2H, Ar*H*), 7.45-7.41 (m, 2H, Ar*H*), 7.36-7.32 (m, 2H, Ar*H*), 7.28 – 7.21 (m, 1H, Ar*H*), 4.56 (q, *J* = 6.6 Hz, 1H, NC*H*), 1.60 (dd, *J* = 6.7 Hz, 3H, CHCH_3), 1.35 (s, 12H, 4 × OCCH_3); $^{13}C\{^1H\}$ NMR (126 MHz, $CDCl_3$) δ_C 159.7, 145.2, 138.9, 135.1, 131.5 (deduced by HMBC), 128.6, 127.6, 127.0, 126.8, 84.1, 70.0, 25.0, 25.0, 24.9; ^{11}B NMR (160 MHz, $CDCl_3$) δ_B 30.0; ^{15}N NMR (51 MHz, $CDCl_3$) δ_N –31.6; HRMS (ESI+): Calculated for [M+H] $C_{17}H_{26}BNO_3S^+$: 336.1799; Found: 336.1804.

(*R*,*E*)-*N*-benzylidene-2-methylpropane-2-sulfinamide (*R*$_S$)-8.

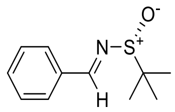

The general procedure 1 was followed using (*R*)-*tert*-butanesulfinamide (*R*$_S$)-**3**(121 mg, 1.00 mmol, 1.00 equiv.) and benzaldehyde (102 µL, 1.00 mmol, 1.00 equiv.) to afford the title compound (*R*$_S$)-**8** (198 mg, 0.95 mmol) as a yellow oil in 95% yield. Characterisation data were consistent with previous literature reports.[16,17]

$[\alpha]_D^{29}$ = −99 ($CHCl_3$, *c* = 1.0) (lit.[17] $[\alpha]_D^{25}$ = +98.3 for (*S*$_S$)-**8**, $CHCl_3$, *c* = 1.07); IR (neat): *v* 2980, 2931, 2867, 1636, 1608, 1573, 1557, 1512, 1493, 1453, 1395, 1356, 1326, 1301, 1270, 1214, 1167, 1142, 1113, 1085, 1018, 963, 909, 856, 833, 780, 761, 740, 702, 671, 655 cm^{-1}; ^1H NMR (500 MHz, $CDCl_3$) δ_H 8.60 (s, 1H, NC*H*), 7.86 (dd, *J* = 8.2, 1.3 Hz, 3H, Ar*H*), 7.57 – 7.43 (m, 3H, Ar*H*), 1.27 (s, 9H, 3 × C*H*$_3$); $^{13}C\{^1H\}$ NMR (126 MHz, $CDCl_3$) δ_C 162.9, 134.3, 132.6, 129.5, 129.1, 57.9, 22.8; ^{15}N NMR (51 MHz, $CDCl_3$) δ_N –55.1; HRMS (ESI+): Calculated for $[M+Na]^+$ $C_{11}H_{15}NOSNa^+$: 232.0767; Found: 232.0767.

(*R*,*E*)-2-methyl-*N*-(4-(4,4,5,5-tetramethyl-1,3,2-dioxaborolan-2-yl)benzylidene)propane-2-sulfinamide (*R*$_S$)-9.

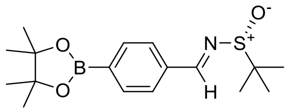

The general procedure 1 was followed using (*R*)-*tert*-butanesulfinamide (*R*$_S$)-**3** (121 mg, 1.00 mmol, 1.00 equiv.) and 4-FPBPin (232 mg, 1.00 mmol, 1.00 equiv.) to afford the title compound (*R*$_S$)-**9** (331 mg, 0.99 mmol) as a yellow solid in 99% yield.

$[\alpha]_D^{29}$= -45 ($CHCl_3$, *c* = 1.0); m.p.: 129-132 °C; IR (neat): *v* 2978, 2147, 2034, 1609, 1591, 1552, 1513, 1467, 1390, 1356, 1332, 1304, 1272, 1217, 1168, 1140, 1089, 1078, 1014, 989, 963, 861, 836, 807, 739, 668, 653 cm^{-1}; ^1H NMR (500 MHz, $CDCl_3$) δ_H 8.60 (s, 1H, N=C*H*), 7.92 – 7.87 (m, 2H, Ar*H*), 7.85 – 7.80 (m, 2H, Ar*H*), 1.36 (s, 12H, 4 × OCC*H*$_3$), 1.27 (s, 9H, 3 × SCC*H*$_3$); $^{13}C\{^1H\}$ NMR (126 MHz, $CDCl_3$) δ_C 163.0, 136.3, 135.4, 133.5 (deduced by HMBC) 128.6, 84.3, 58.1, 25.0, 25.0, 22.8; ^{11}B NMR (160 MHz, $CDCl_3$) δ_B 30.4; ^{15}N NMR (51 MHz, $CDCl_3$) δ_N –51.7; HRMS (ESI+): Calculated for $[M+Na]^+$ $C_{17}H_{26}BNO_3SNa^+$: 358.1622; Found: 358.1621.

7.3 Synthesis of IB complexes

(*R*)-2-methyl-*N*-((*E*)-2-((3a*R*,4*R*,6*R*,7a*S*)-3a,5,5-trimethylhexahydro-4,6-methanobenzo[*d*][1,3,2]dioxaborol-2-yl)benzylidene)propane-2-sulfinamide (*R*$_S$,*R*)-5a.

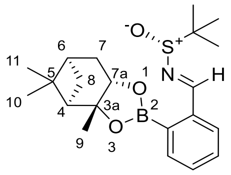

(*R*)-Ellman's sulfinamide (*R*$_S$)-**3** (61 mg, 0.50 mmol) was added to a stirred suspension of 2-formylbenzene boronic acid (90 mg, 0.60 mmol, 1.2 equiv.) and $MgSO_4$ (1.00 g) in $CHCl_3$ (5.0 mL) and the reaction stirred for 2 h, with (1*R*,2*R*,3*S*,5*R*)-pinanediol (111 mg, 0.65 mmol, 1.3 equiv.) then added. After 10 min, the reaction was filtered and concentrated to dryness *in vacuo* and the residue purified by chromatography (0.5% MeOH in 1:1 CH_2Cl_2/*n*-hexane), affording the title compound (*R*$_S$,*R*)-**5a** (24 mg, 0.062 mmol) as a clear oil in 12% yield as a 89:11 mixture with its parent formyl boronate ester **11**.[1]

^{1}H NMR (500 MHz, $CDCl_3$) δ_H 9.36 (s, 1H, NC*H*), 8.13-8.06 (m, 1H, Ar*H*), 7.94-7.88 (m, 1H, Ar*H*), 7.54-7.46 (m, 2H, Ar*H*), 4.51 (dd, 1H, *J* = 8.8, 2.0, H-7a), 2.48-2.37 (m, 1H, H-7), 2.29-2.21 (m, 1H, H-8), 2.18 (dd, 1H, *J* = 6.1, 5.1, H-4), 2.02 (ddd, 1H, *J* = 14.7, 3.4, 2.0, H-7), 1.97-1.97 (m, 1H, H-6), 1.51 (s, 3H,H-9), 1.30 (s, 3H, H-10/11), 1.26 (s, 9H, *tert*-butyl), 1.23 (d, 1H, *J* = 10.9, H-8), 0.88 (s, 3H, H-10/11); ^{11}B NMR (375.5 MHz, $CDCl_3$) δ_B 30.5; ^{15}N NMR (51 MHz, $CDCl_3$) δ_N –53.3; HRMS (ESI+): Calculated for $[M+H]^+$ $C_{21}H_{31}BNO_3S$: 388.2116, Found 388.2118; Calculated for $[M+Na]^+$ $C_{21}H_{30}BNO_3SNa^+$: 410.1936; Found: 410.1940.

(*S*)-2-methyl-*N*-((*E*)-2-((3a*R*,4*R*,6*R*,7a*S*)-3a,5,5-trimethylhexahydro-4,6-methanobenzo[*d*][1,3,2]dioxaborol-2-yl)benzylidene)propane-2-sulfinamide (*S*$_S$,*R*)-5b.

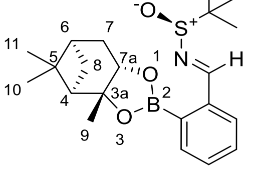

The same procedure as above was followed using (*S*)-Ellman's sulfinamide (*S*$_S$)-**3**, affording the title compound (*S*$_S$,*R*)-**5b** (37 mg, 0.096 mmol) as a clear oil in 19% yield, as a 96:4 mixture with its parent formyl boronate ester **11**.[1]

^{1}H NMR (500 MHz, $CDCl_3$) δ_H 9.27 (s, 1H, NC*H*), 8.08-8.03 (m, 1H, Ar*H*), 7.90-7.83 (m, 1H, Ar*H*), 7.54-7.47 (m, 2H, Ar*H*), 4.51 (dd, 1H, *J* = 8.7, 1.9, H-7a), 2.49-2.38 (m, 1H, H-7), 2.32-2.21 (m, 1H, H-8), 2.17 (dd, 1H, *J* = 6.0, 5.0, H-4), 2.09-1.91 (m, 2H H-7 + H-6), 1.51 (s, 3H, H-9), 1.31 (s, 3H, H-10/11), 1.28-1.22 (m, 12H, tert-butyl + H-8), 0.88 (s, 3H, H10/11); ^{11}B NMR (375.5 MHz, $CDCl_3$) δ_B 31.2; ^{15}N

NMR (51 MHz, $CDCl_3$) δ_N –51.7; HRMS (ESI+): Calculated for $[M+H]^+$ $C_{21}H_{31}BNO_3S^+$: 388.2116, Found 388.2112; Calculated for $[M+Na]^+$ $C_{21}H_{30}BNO_3S$: 410.1936; Found: 410.1937.

(*E*)-(2-(((*tert*-butylsulfinyl)imino)methyl)phenyl)boronic acid (*S*$_S$)-4.

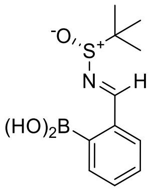

(*R*$_S$)-Ellman's sulfinamide (*S*$_S$)-**3** (33 mg, 0.27 mmol, 1.35 equiv.) was added to a stirred suspension of 2-FPBA (30 mg, 0.20 mmol, 1.0 equiv.) and $MgSO_4$ (500 mg) in $CDCl_3$ (2.0 mL) and the reaction stirred for 2 h, before filtering through a cotton wool-celite plug. The title compound (*S*)-**4** was formed in solution in 95% yield (5% 2-FPBA and 0.40 equiv. (*S*$_S$)-**3** remaining in solution). The product was analysed and characterised in solution and was not isolated.

^{1}H NMR (500 MHz, $CDCl_3$) δ_H 9.12 (s, 1H, NC*H*), 8.15-8.10 (m, 1H, Ar*H*), 7.96-7.89 (m, 1H, Ar*H*), 7.59-7.53 (m, 2H, Ar*H*), 7.19 (bs, 1H,2 x OH), 1.30 (s, 9H, 3 x C*H$_3$*), 0.88 (s, 3H, H-10/11); ^{13}C NMR (126 MHz, $CDCl_3$) δ_C 167.3, 138.0, 137.2, 134.7 (deduced by HMBC), 132.1, 132.0, 130.8, 58.3, 22.6.;^{11}B NMR (375.5 MHz, $CDCl_3$) δ_B 28.6; ^{15}N NMR (51 MHz, $CDCl_3$) δ_N –67.0; HRMS (ESI+): Calculated for $[M-2HO^- +2MeO^-+Na]^+$ $C_{13}H_{20}BNO_3SNa^+$: 304.1149, Found 304.1138. Slow evaporation from $CDCl_3$/*n*-hexane afforded white crystals suitable for X-ray crystallography (*vide infra*).

8 NMR spectra of pure compounds

8.1 ^{1}H, ^{13}C and ^{15}N NMR spectra of pure compounds

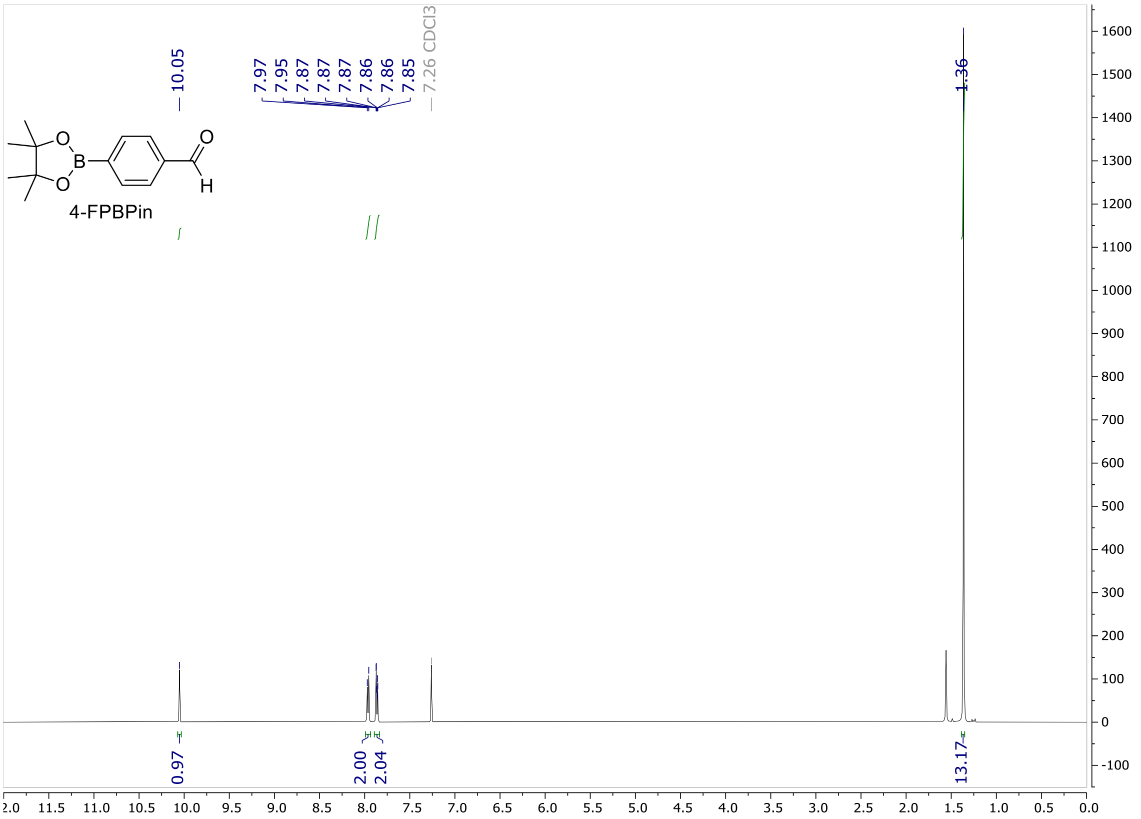

Figure S8: ^{1}H NMR (500 MHz, $CDCl_3$) of 4-FPBPin.

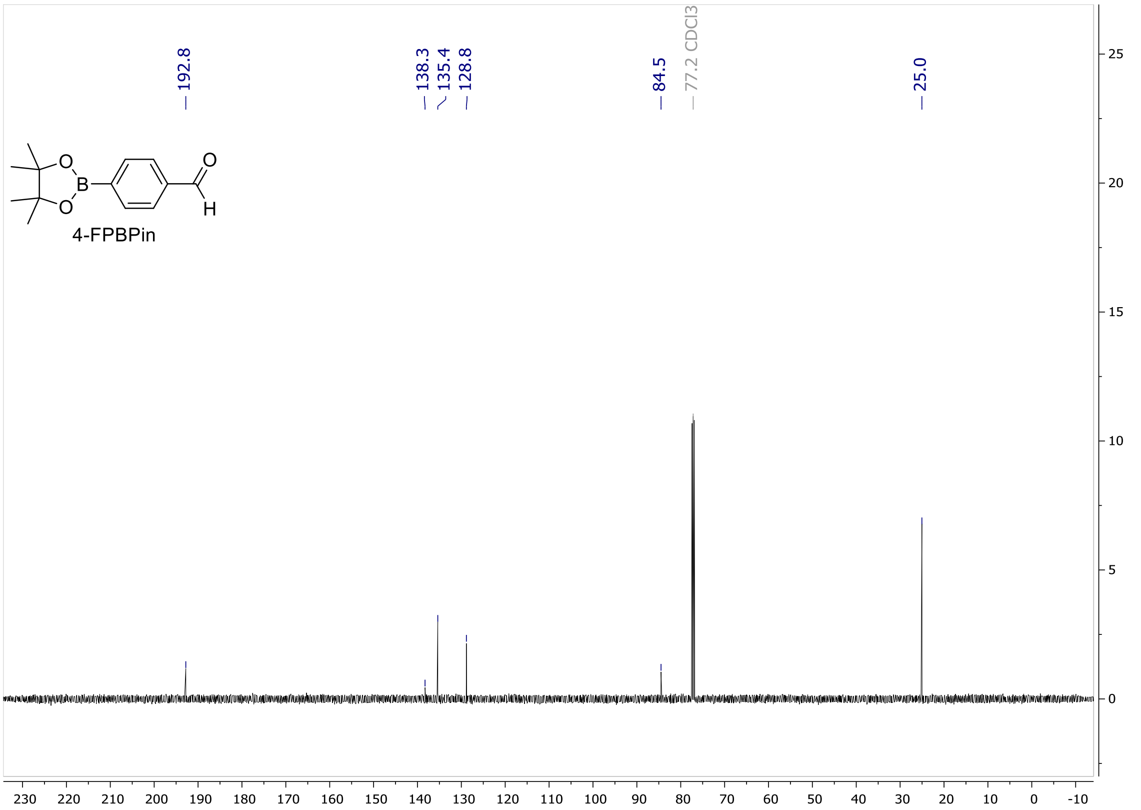

Figure S9: $^{13}C\{^{1}H\}$ NMR (126 MHz, $CDCl_3$) of 4-FPBPin.

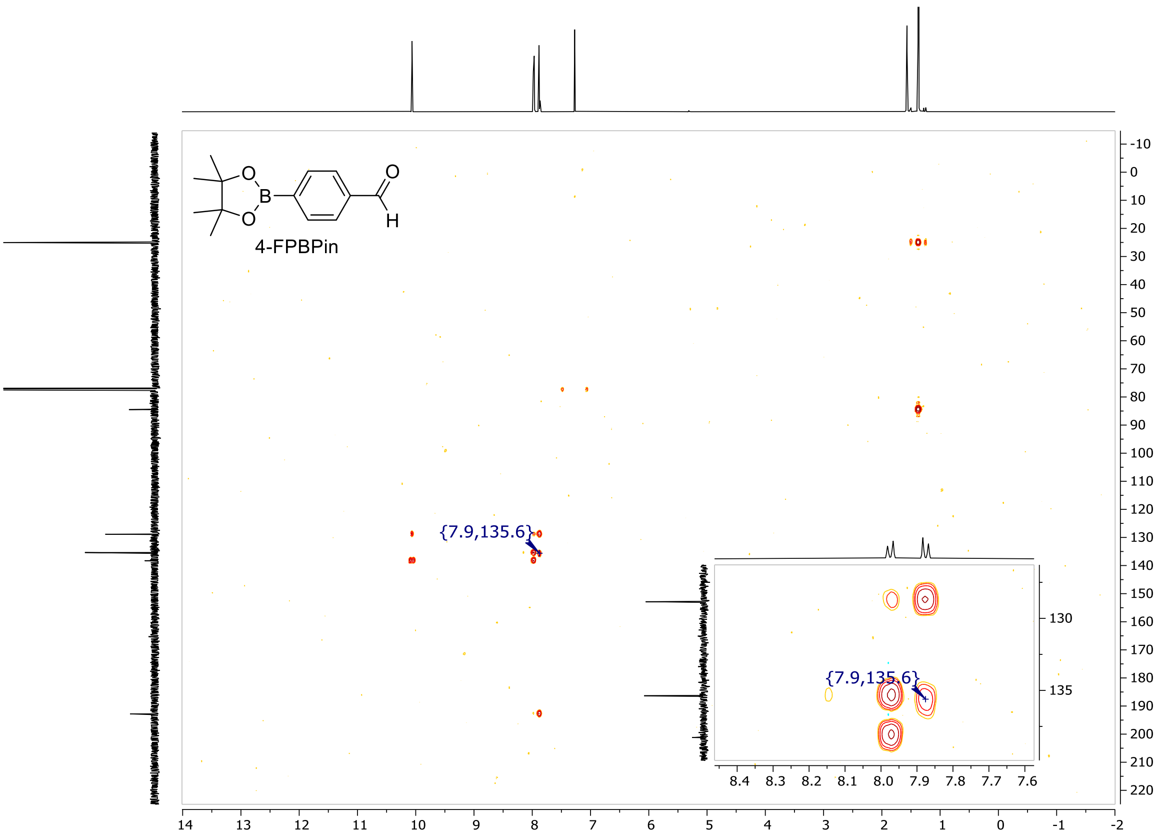

Figure S10: ^{1}H-^{13}C HMBC NMR (500, 126 MHz, $CDCl_3$) of 4-FPBPin. Ar*H*-C(B) correlations shown.

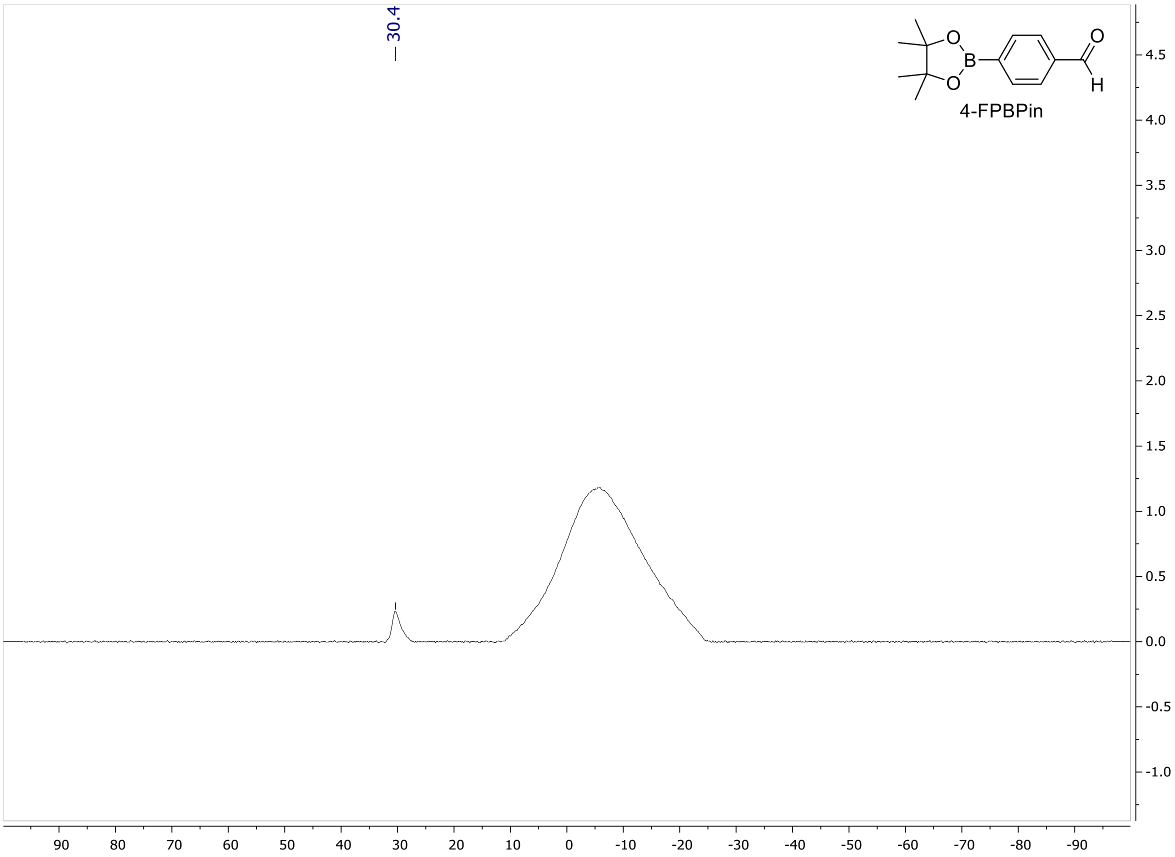

Figure S11: ^{11}B NMR (160 MHz, $CDCl_3$) of 4-FPBPin.

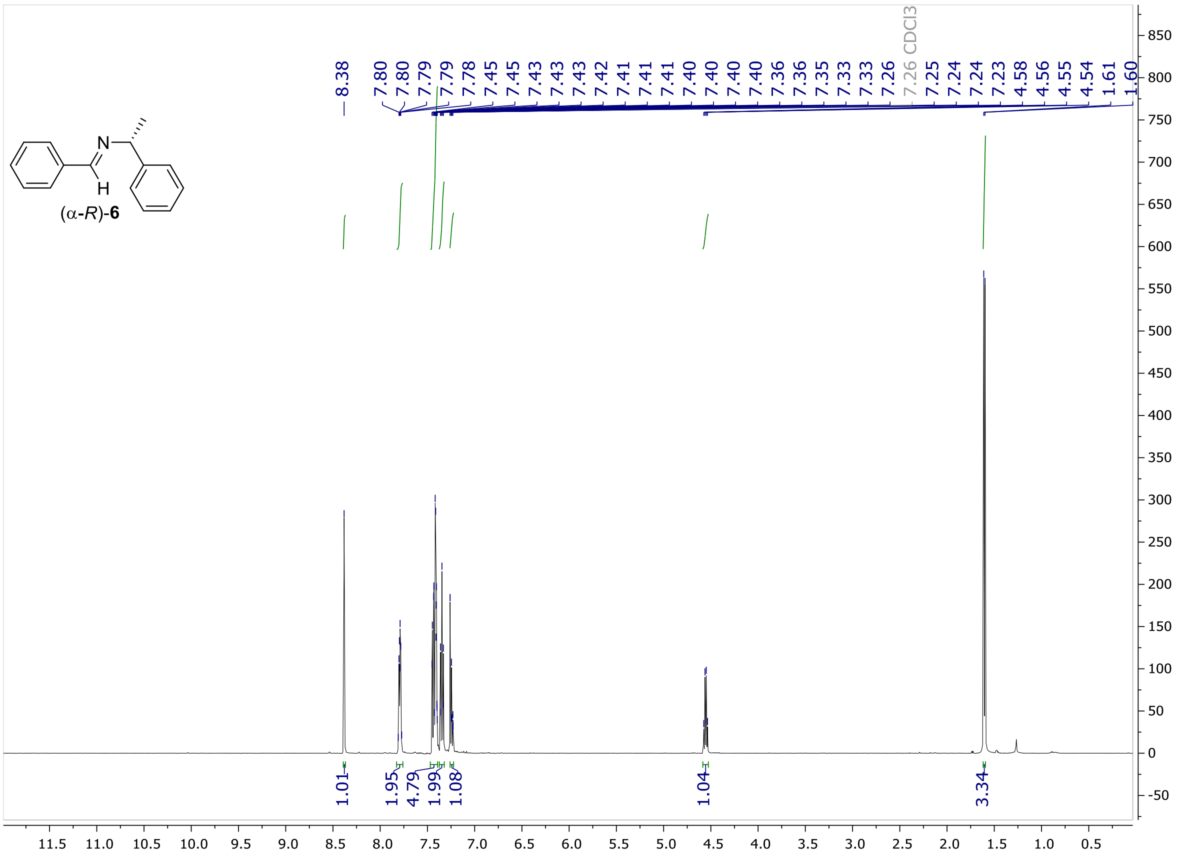

Figure S12: ^{1}H NMR (500 MHz, $CDCl_3$) of (α-*R*)-**6**.

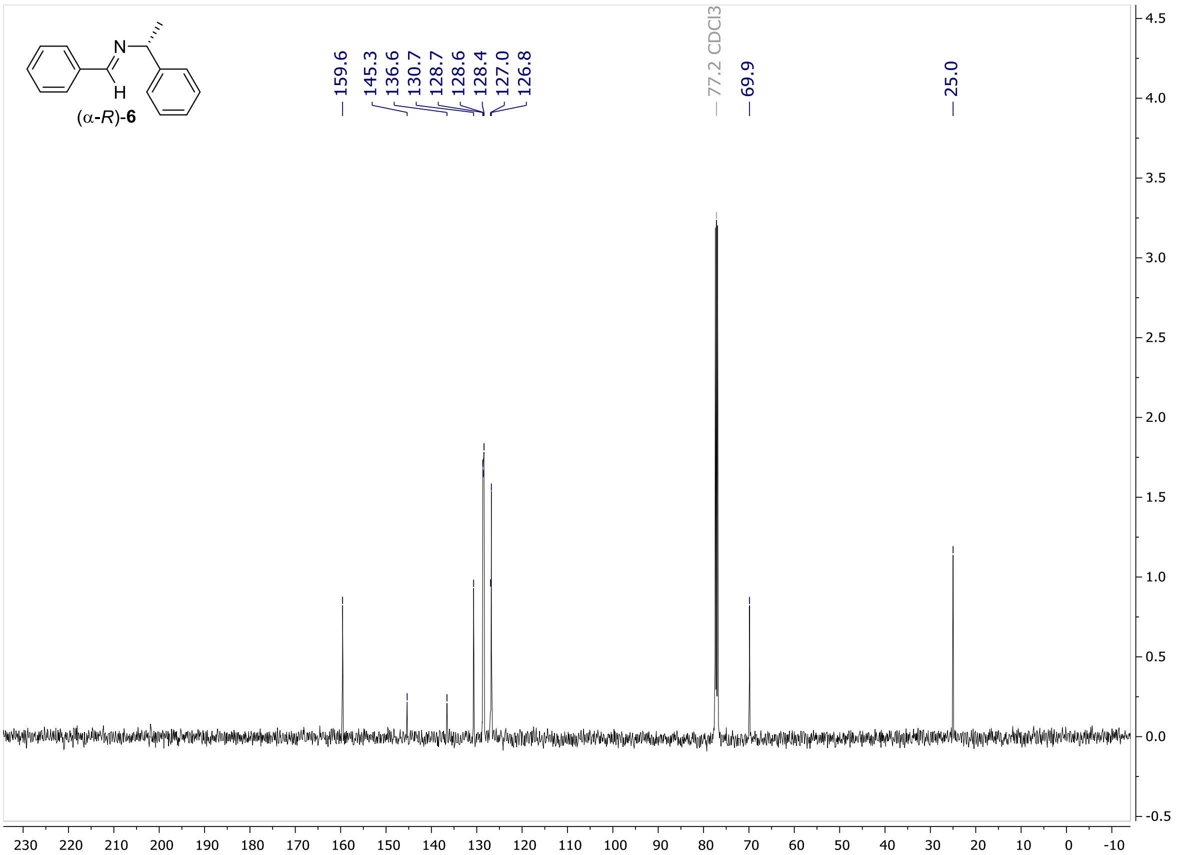

Figure S13: $^{13}C\{^{1}H\}$ NMR (126 MHz, $CDCl_3$) of (α-*R*)-**6**.

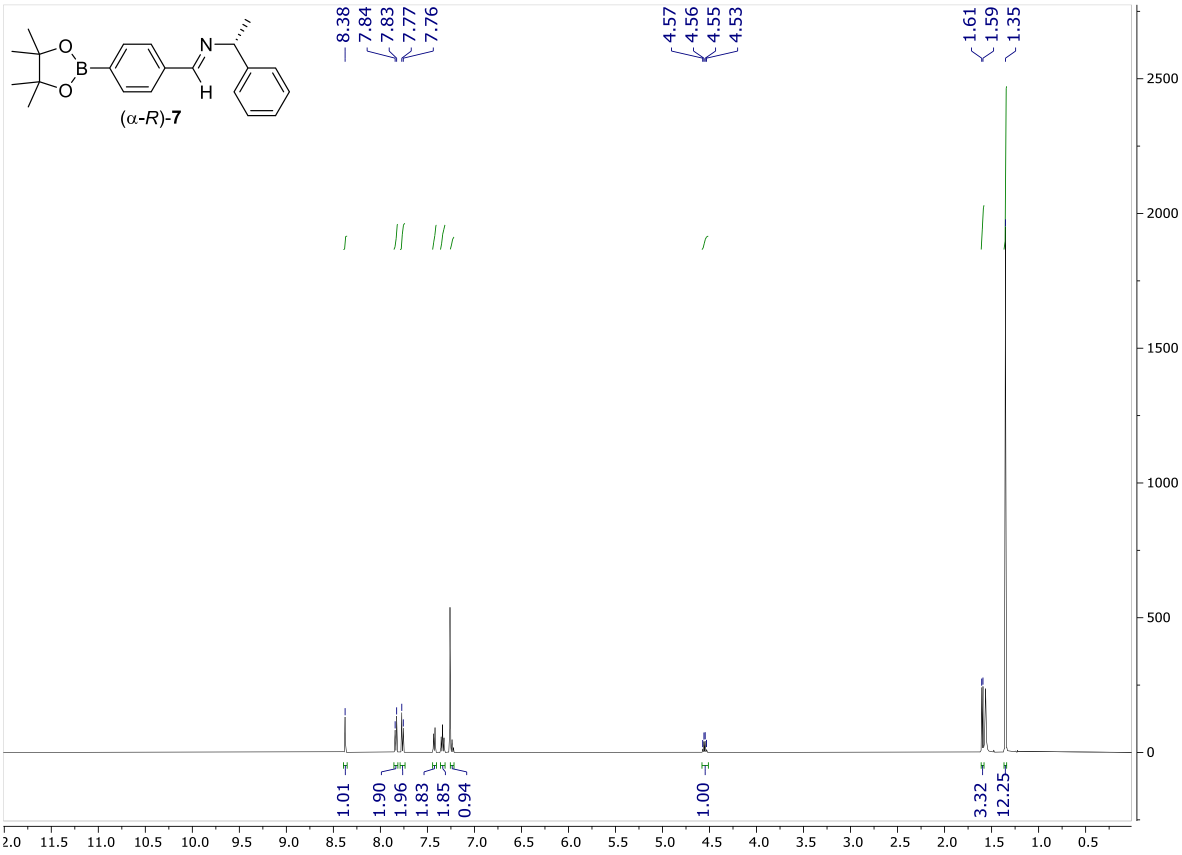

Figure S14: ^{1}H NMR (500 MHz, $CDCl_3$) of (α-*R*)-**7**.

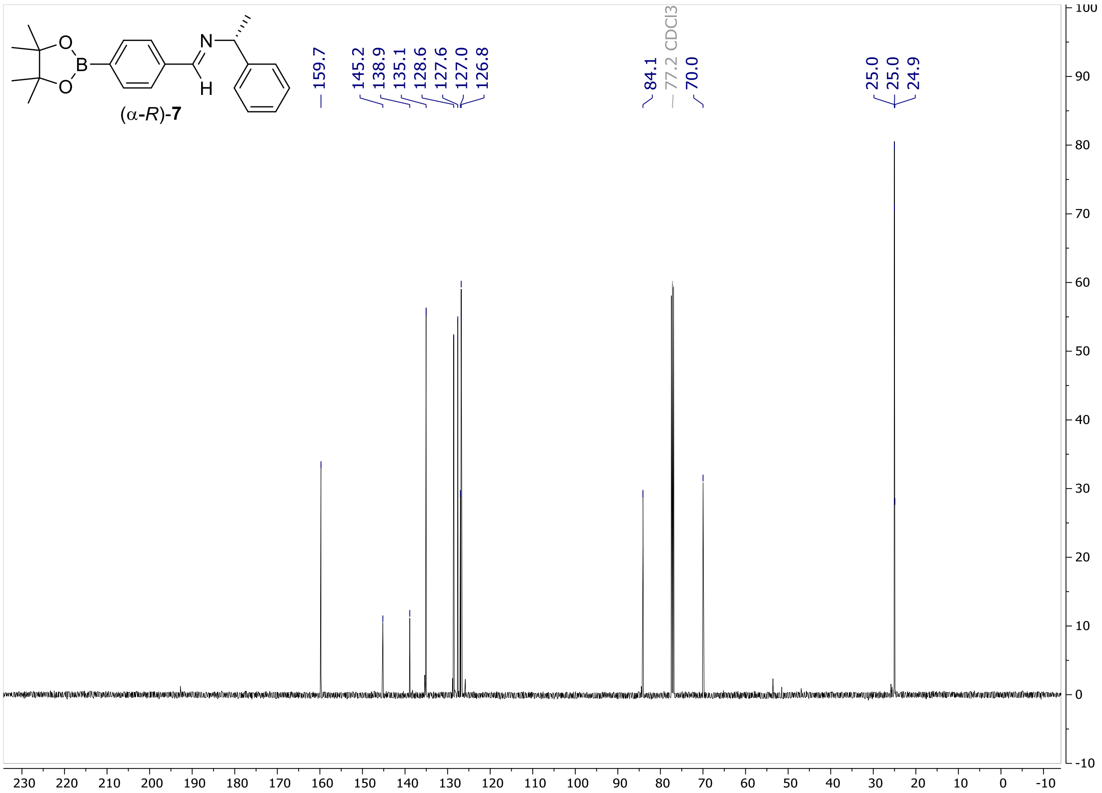

Figure S15: $^{13}C\{^{1}H\}$ NMR (126 MHz, $CDCl_3$) of (α-*R*)-**7**.

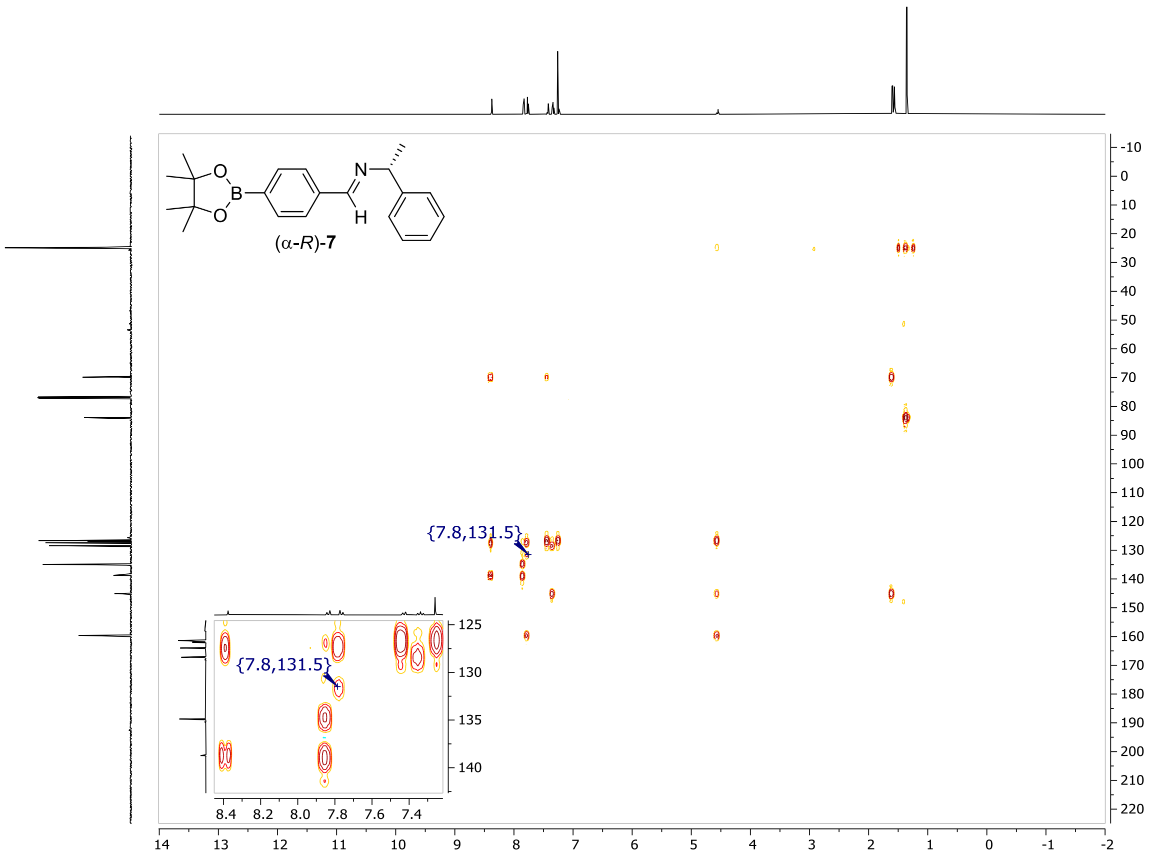

Figure S16: ^{1}H-^{13}C HMBC NMR (500, 126 MHz, $CDCl_3$) of (α-*R*)-**7**. Ar*H*-C(B) correlations shown.

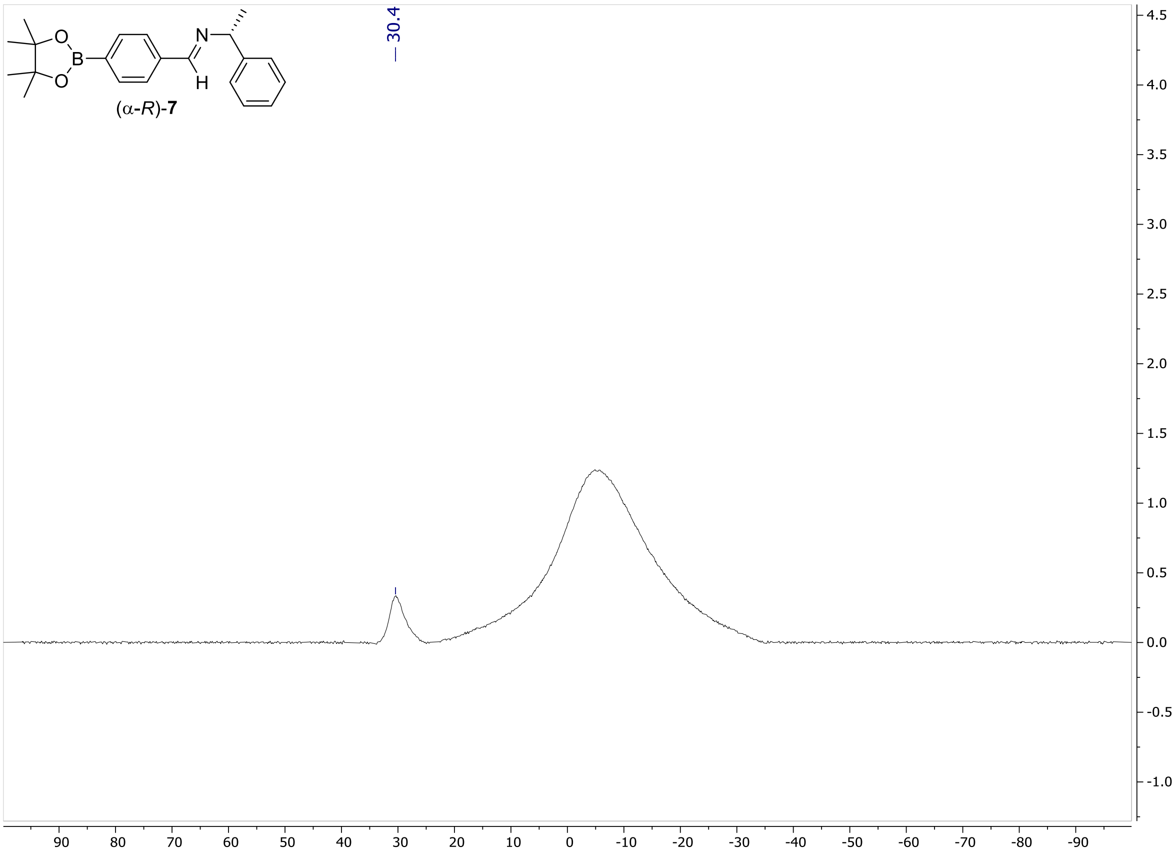

Figure S17: ^{11}B NMR (160 MHz, $CDCl_3$) of (α-*R*)-**7**.

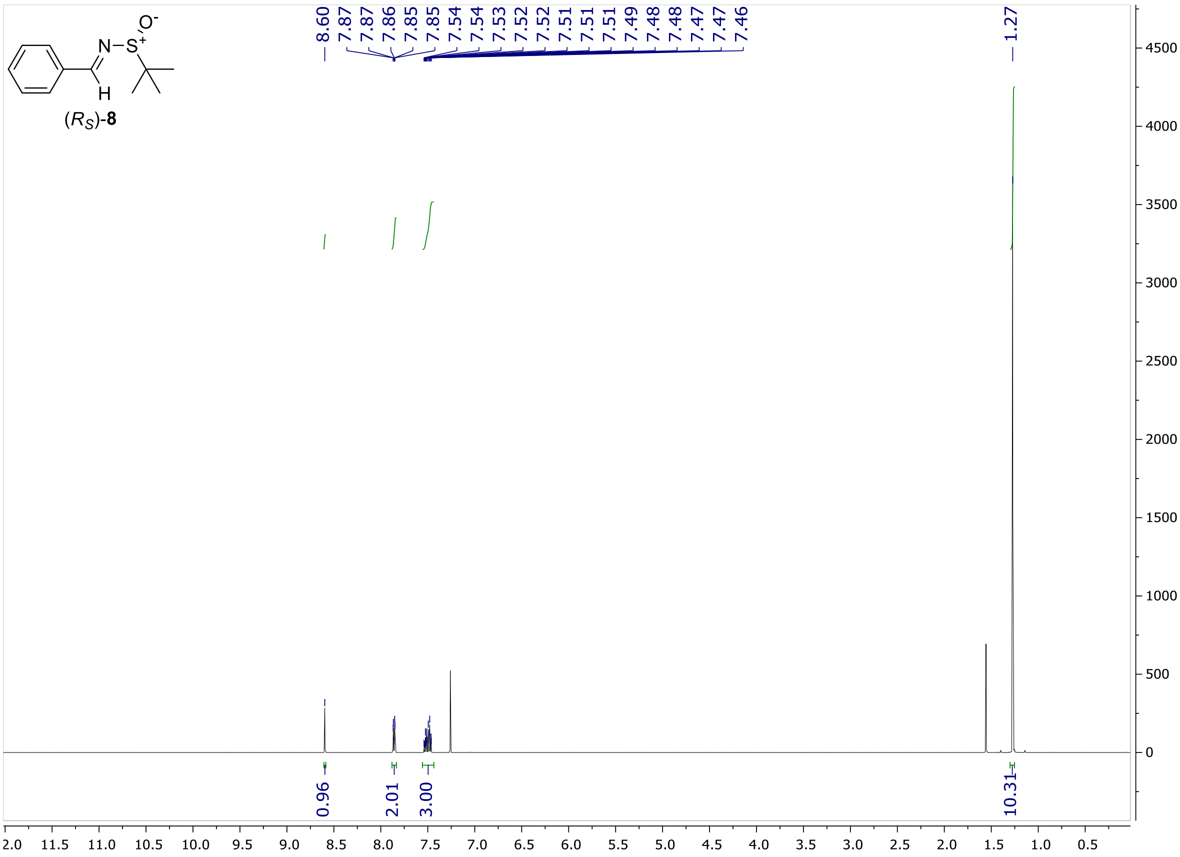

Figure S18: ^{1}H NMR (500 MHz, $CDCl_3$) of (R_S)-**8**.

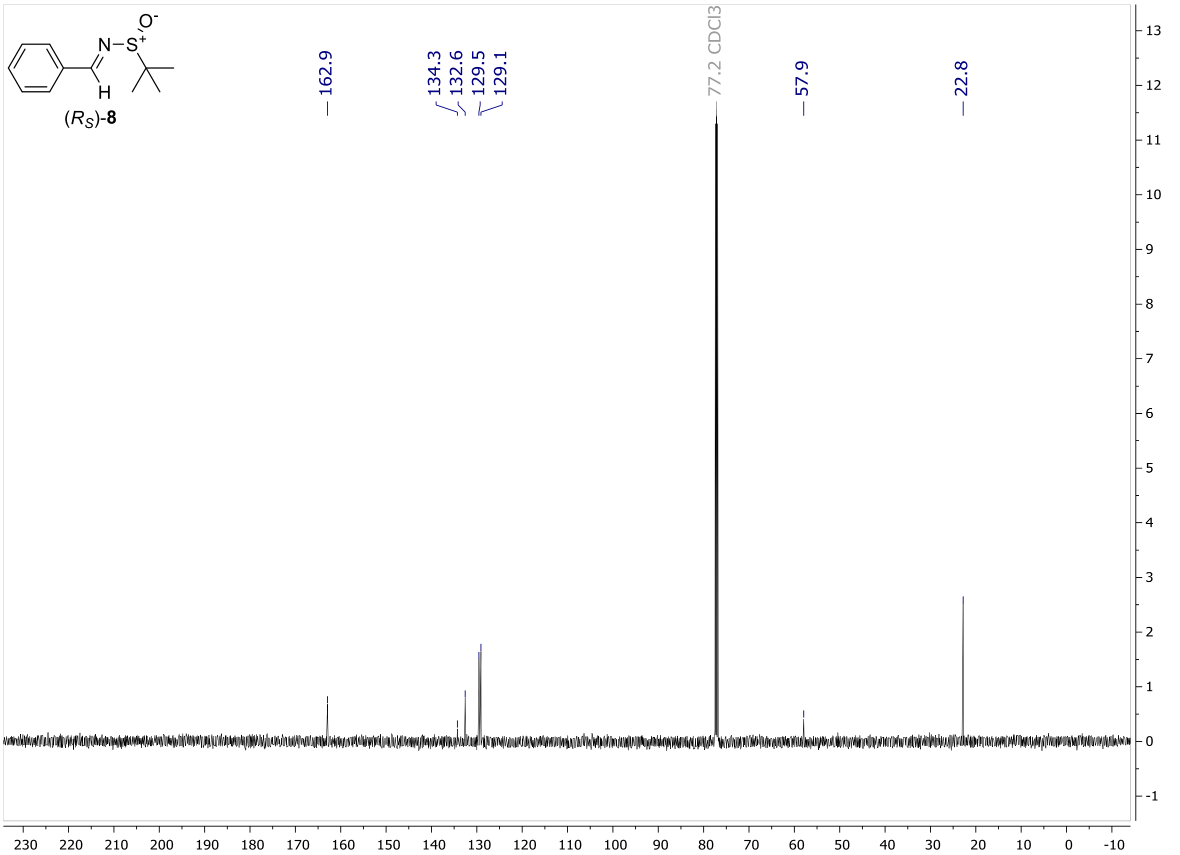

Figure S19: $^{13}C\{^{1}H\}$ NMR (126 MHz, $CDCl_3$) of (R_S)-**8**.

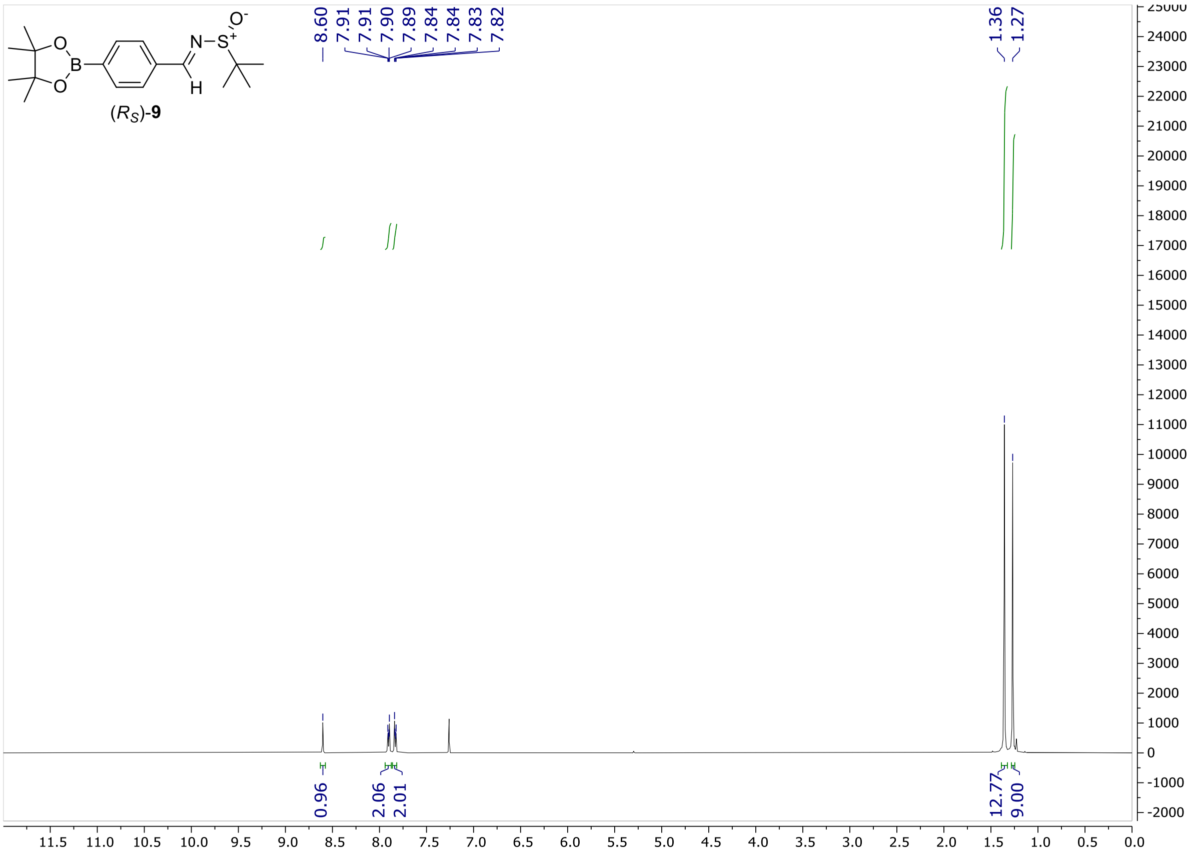

Figure S20: ^{1}H NMR (500 MHz, $CDCl_3$) of (R_S)-**9**.

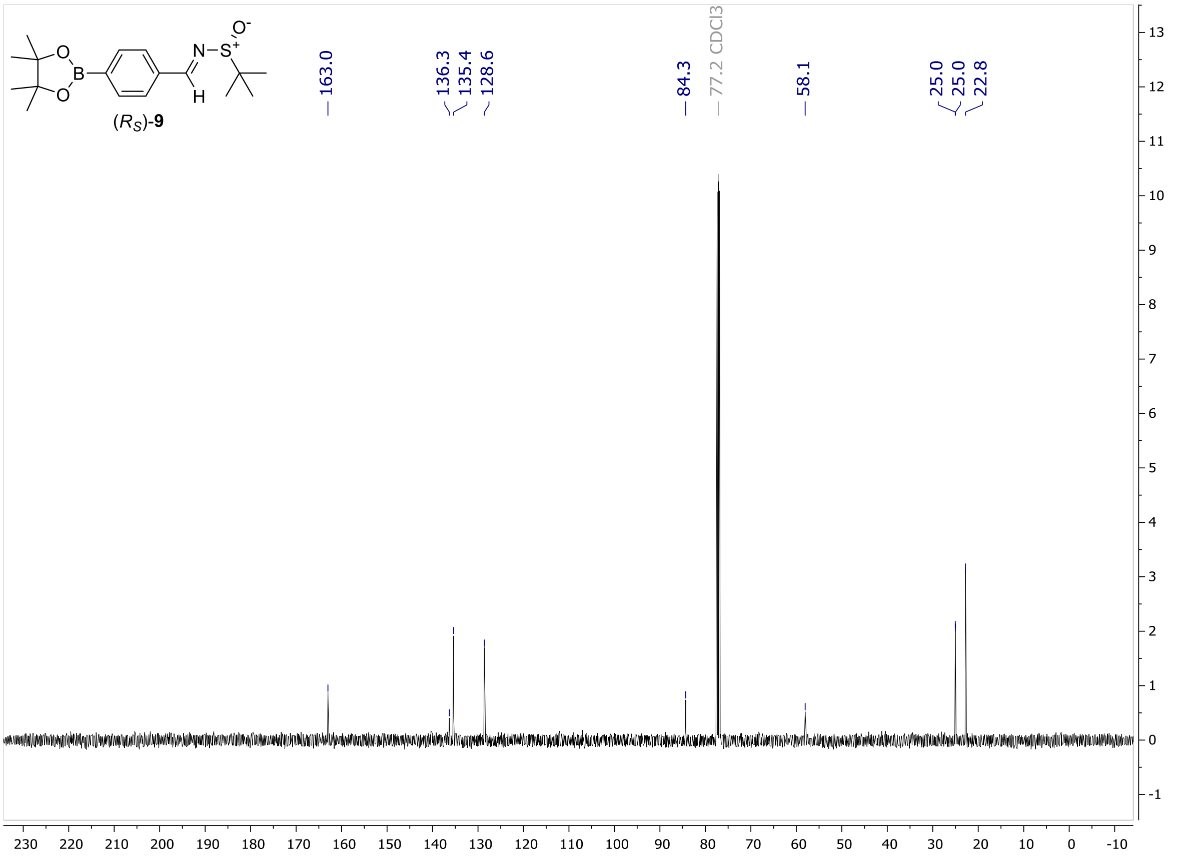

Figure S21: $^{13}C\{^{1}H\}$ NMR (126 MHz, $CDCl_3$) of (R_S)-**9**.

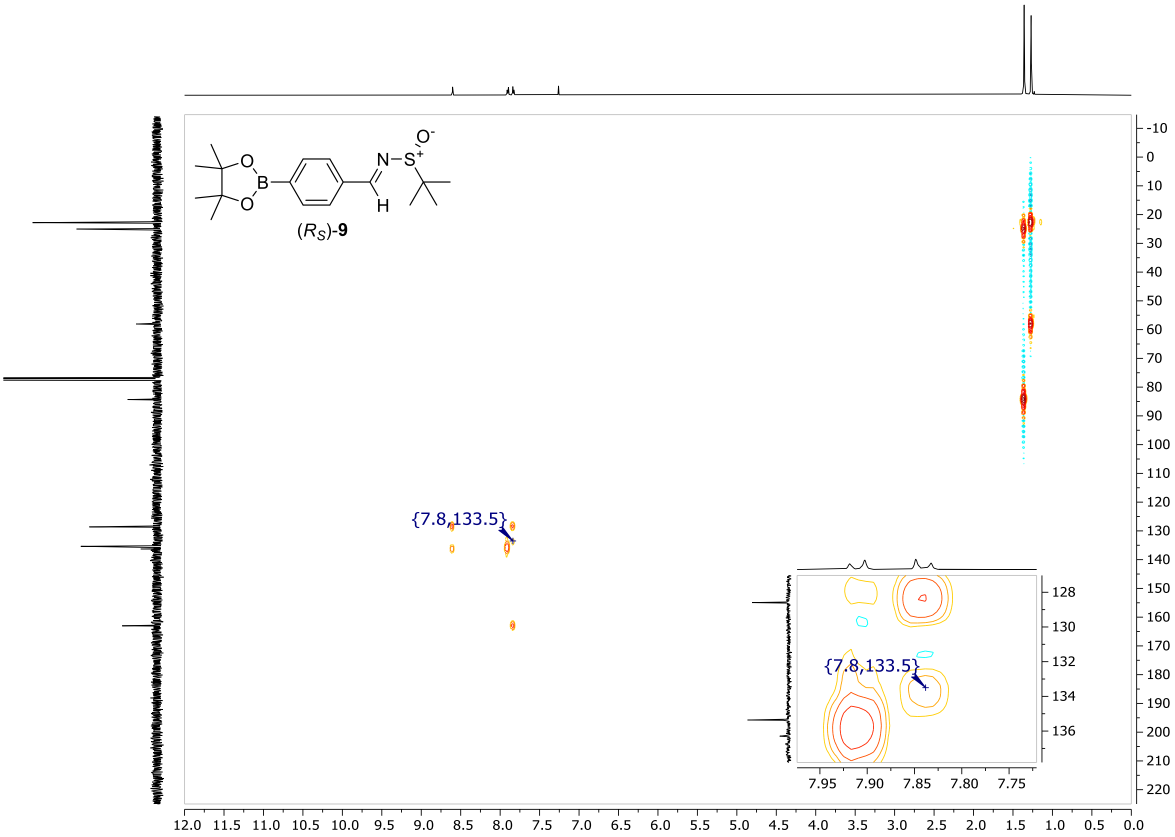

Figure S22: ^{1}H-^{13}C HMBC NMR (500, 126 MHz, $CDCl_3$) of (R_S)-**9**. Ar*H*-C(B) correlations shown.

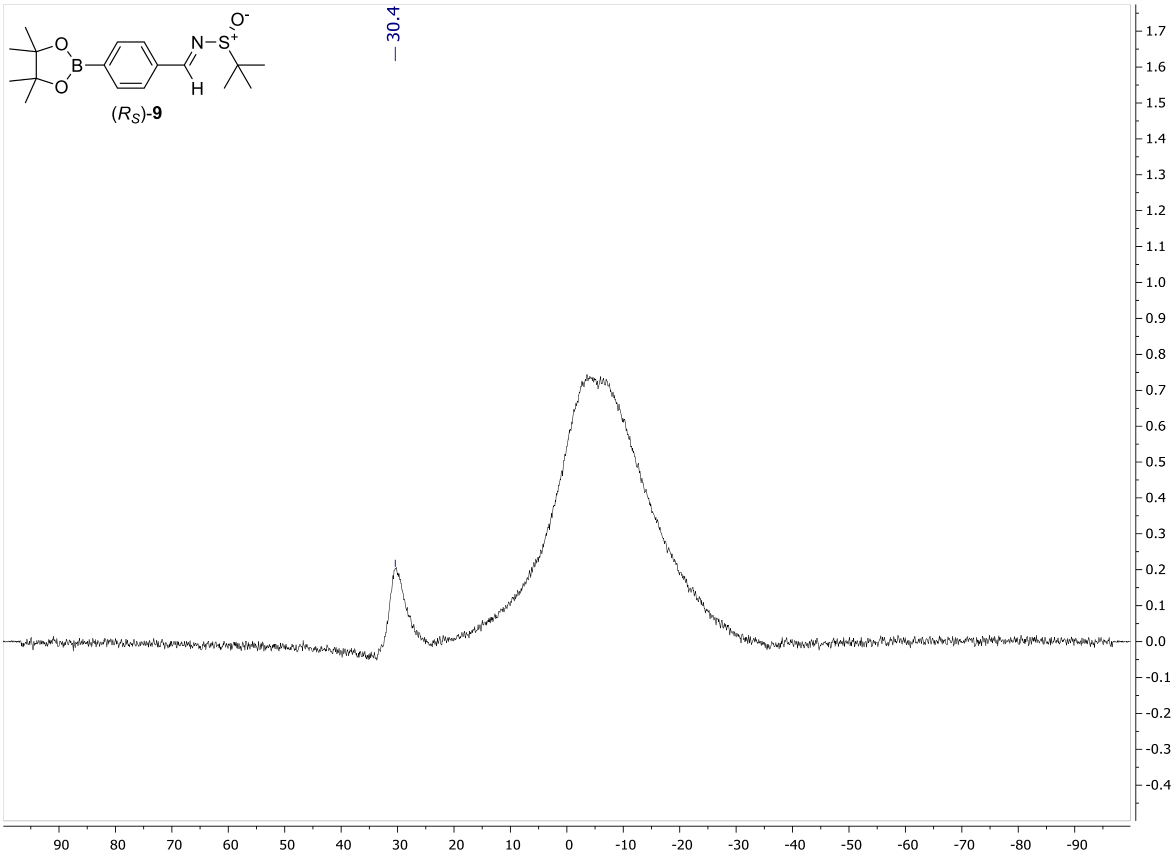

Figure S23: ^{11}B NMR (160 MHz, $CDCl_3$) of (R_S)-**9**.

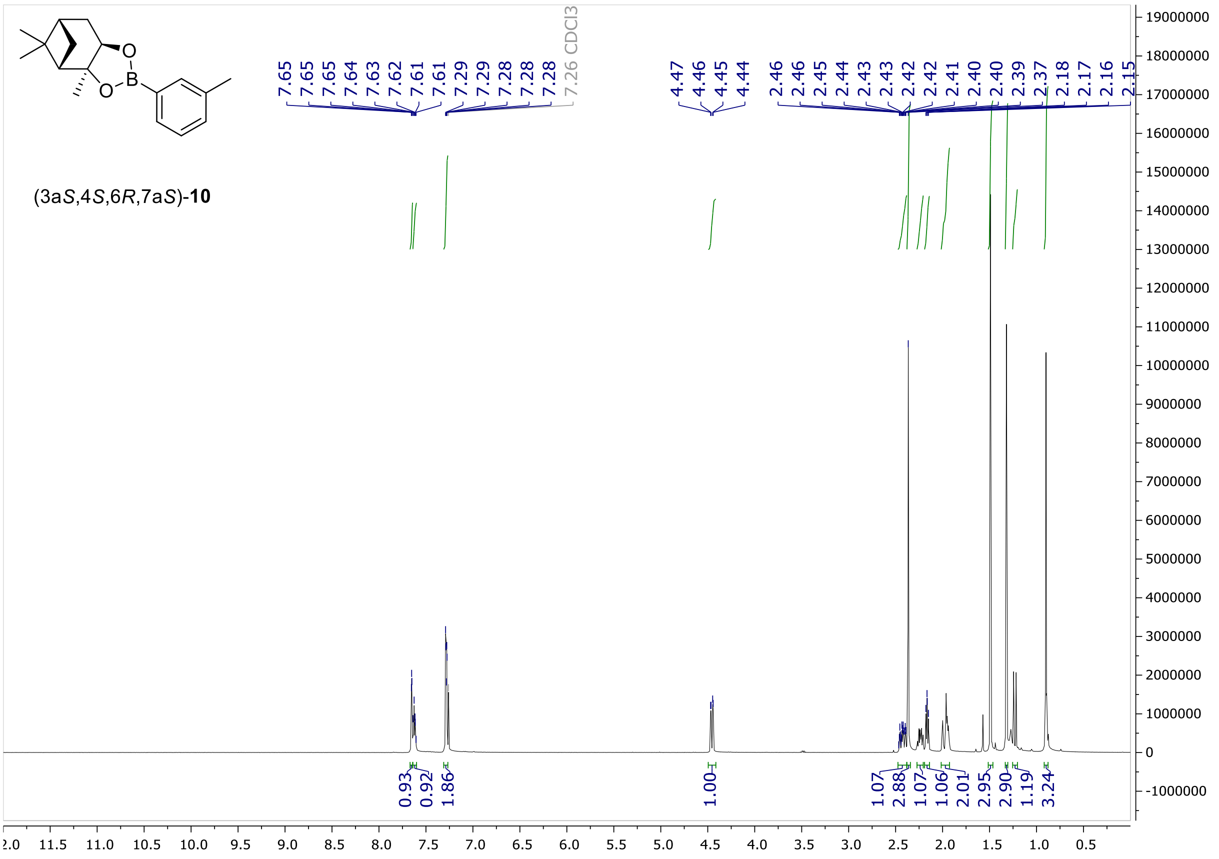

Figure S24: ^{1}H NMR (500 MHz, $CDCl_3$) of (3a*R*,4*R*,6*R*,7a*S*)-**10**.

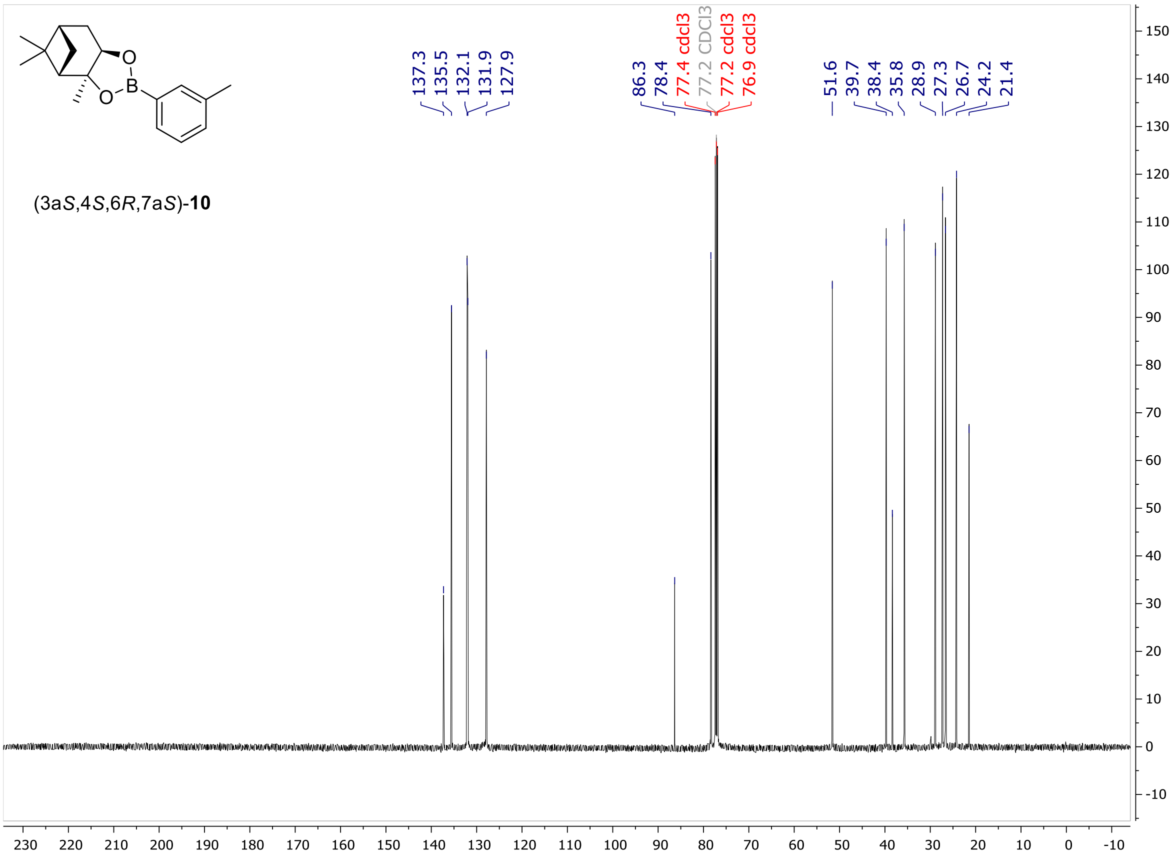

Figure S25: $^{13}C\{^{1}H\}$ NMR (126 MHz, $CDCl_3$) of (3a*R*,4*R*,6*R*,7a*S*)-**10**.

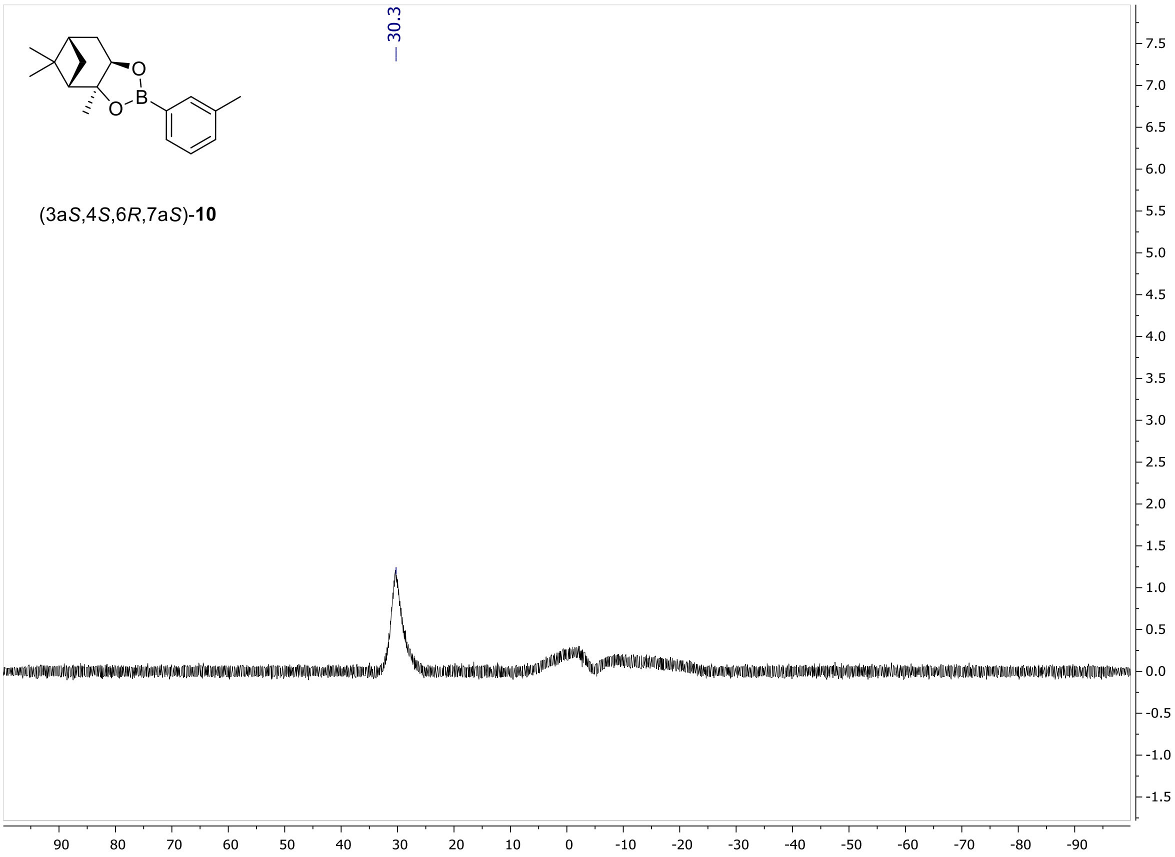

Figure S26: ^{11}B NMR (160 MHz, $CDCl_3$) of (3a*R*,4*R*,6*R*,7a*S*)-**10**.

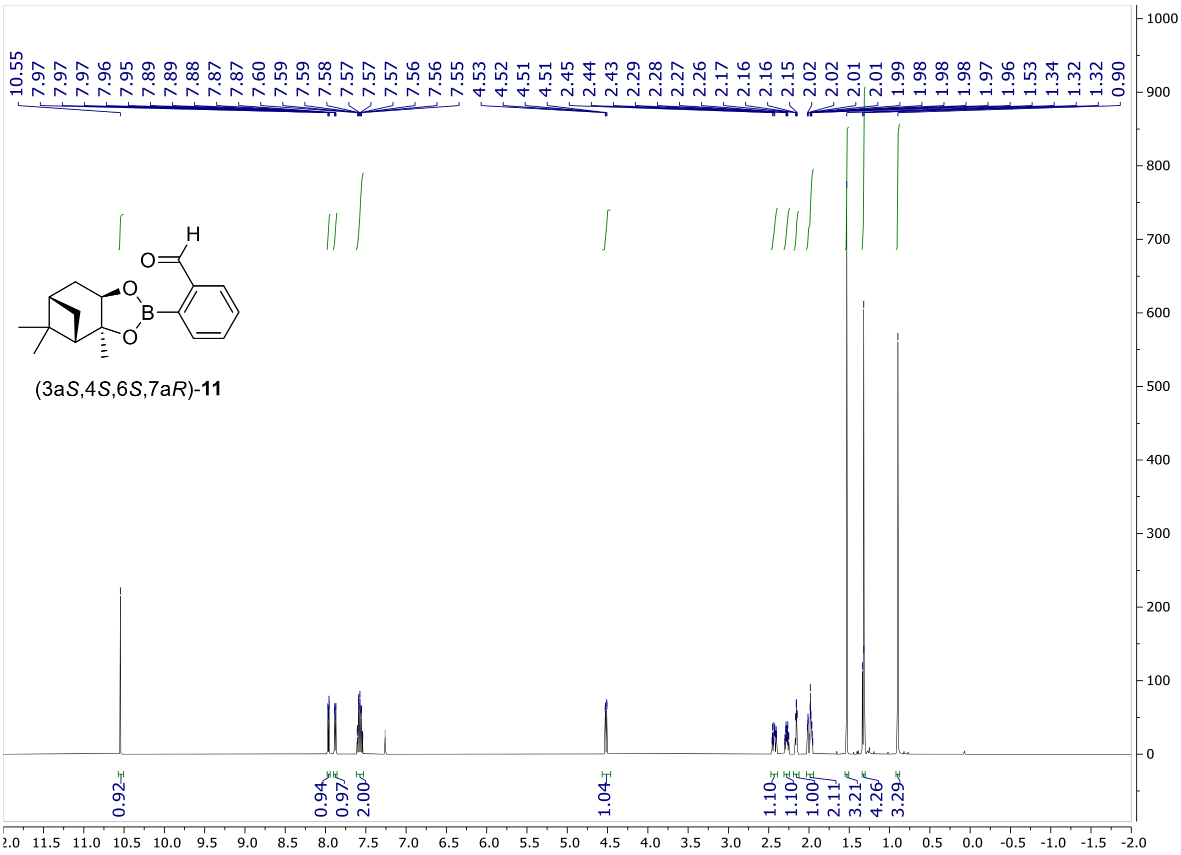

Figure S27: ^{1}H NMR (500 MHz, $CDCl_3$) of (3a*S*,4*S*,6*S*,7a*R*)-**11**.

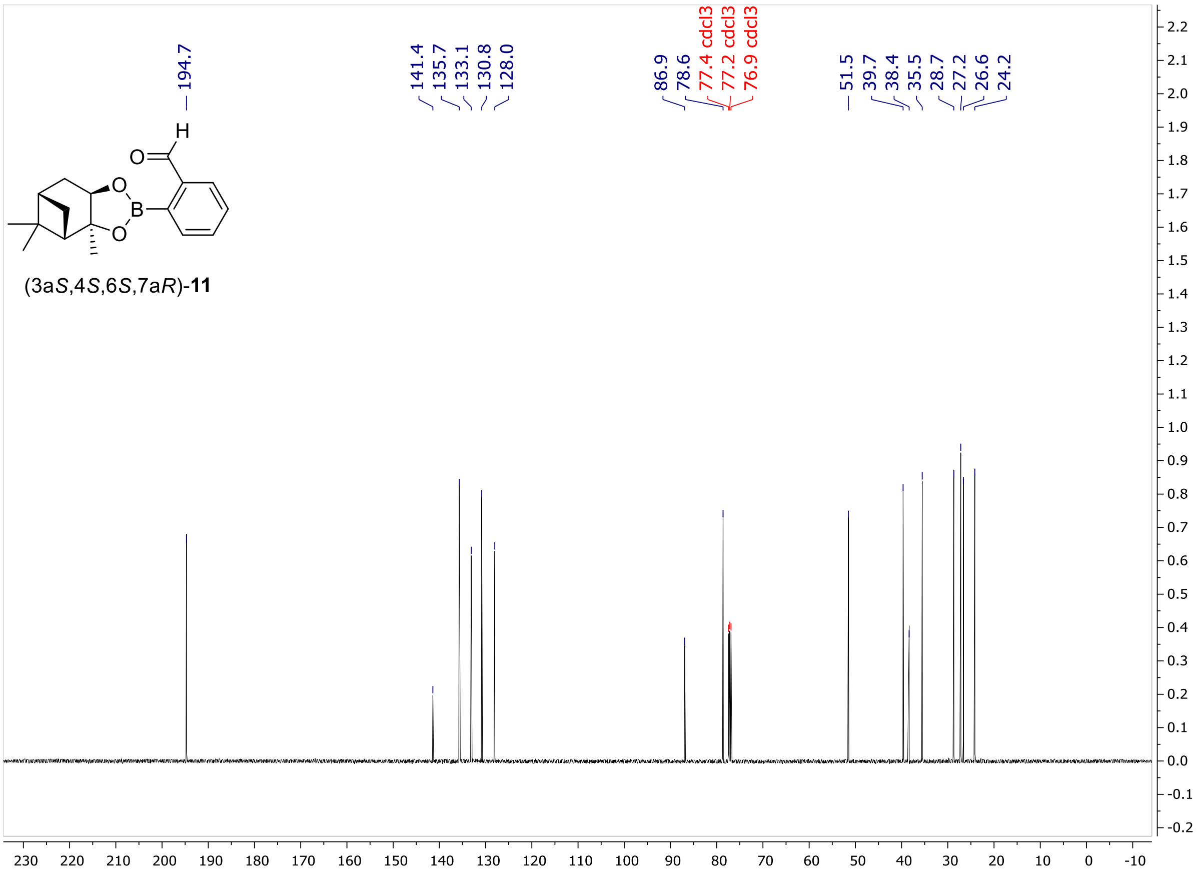

Figure S28: $^{13}C\{^{1}H\}$ NMR (126 MHz, $CDCl_3$) of (3a*S*,4*S*,6*S*,7a*R*)-**11**.

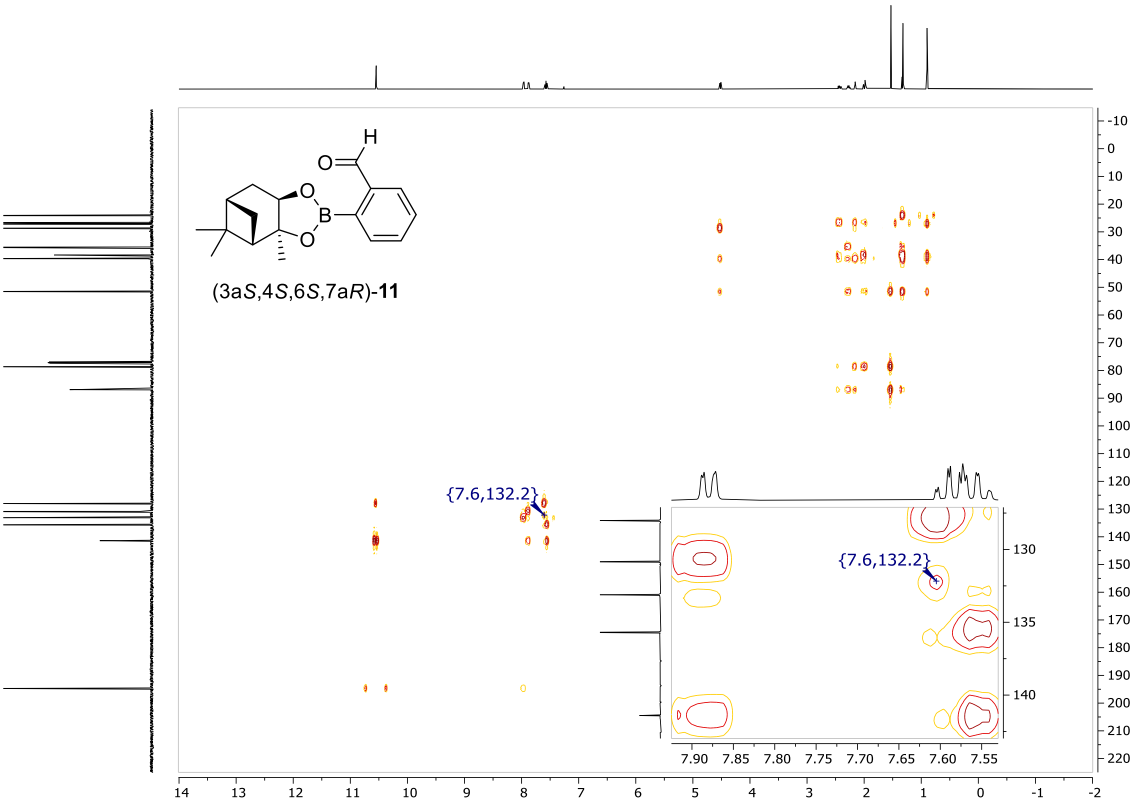

Figure S29: ^{1}H-^{13}C HMBC NMR (500, 126 MHz, $CDCl_3$) of (3a*S*,4*S*,6*S*,7a*R*)-**11**. Ar*H*-C(B) correlations shown. See Groleau *et al.*[1] for confirmation by variable temperature ^{13}C NMR.

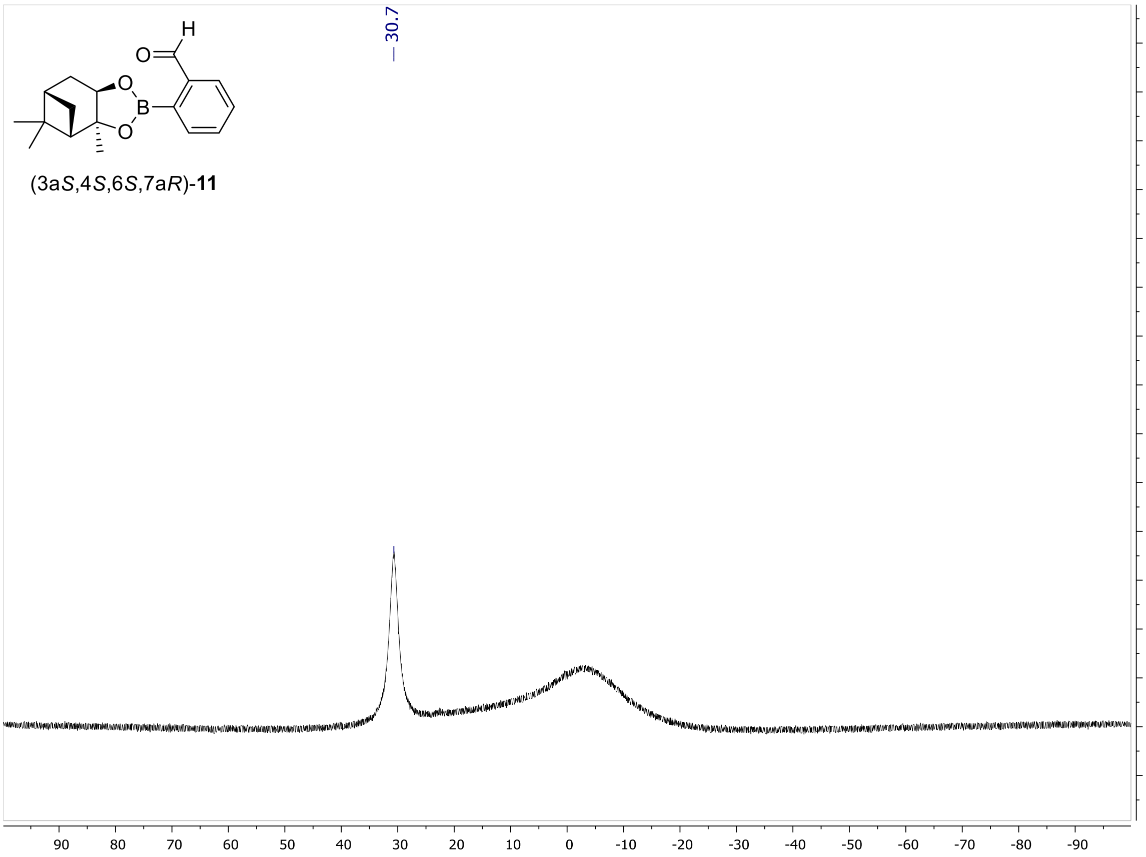

Figure S30: ^{11}B NMR (160 MHz, $CDCl_3$) of (3a*S*,4*S*,6*S*,7a*R*)-**11**.

8.2 ^{15}N NMR spectra of pure compounds

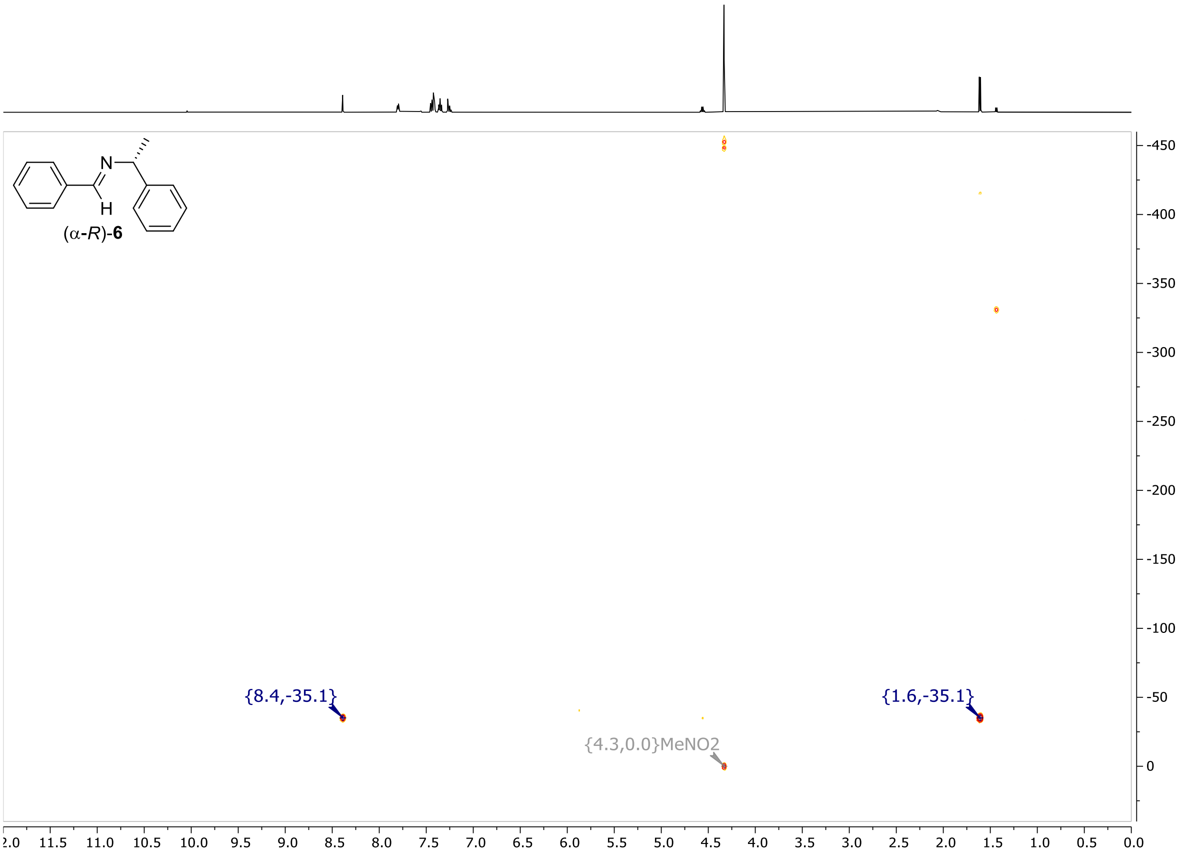

Figure S31: ^{1}H-^{15}N HMBC NMR (500, 51 MHz, $CDCl_3$, 50 mM, ref. 50 mM $MeNO_2$) of (α-*R*)-**6**.

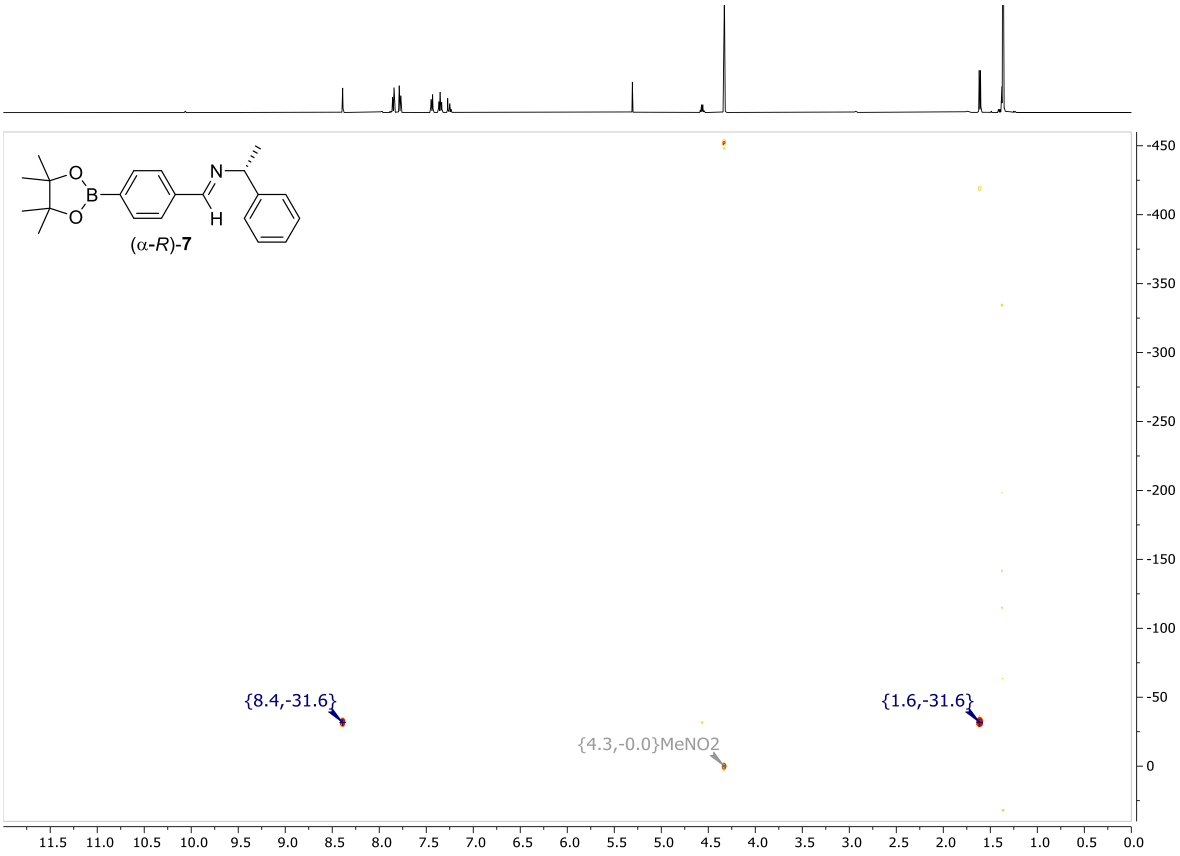

Figure S32: ^{1}H-^{15}N HMBC NMR (500, 51 MHz, $CDCl_3$, 50 mM, ref. 50 mM $MeNO_2$) of (α-*R*)-**7**.

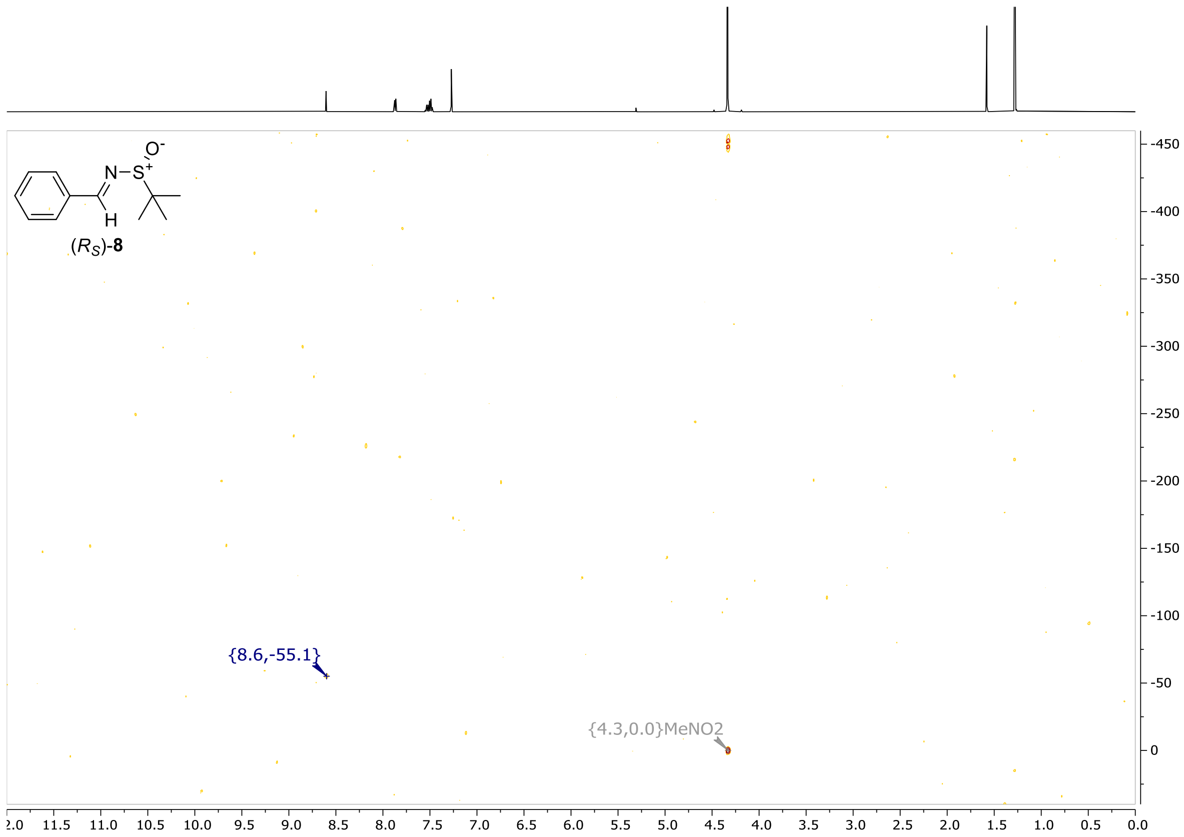

Figure S33: ^{1}H-^{15}N HMBC NMR (500, 51 MHz, $CDCl_3$, 50 mM, ref. 50 mM $MeNO_2$) of (R_S)-**8**.

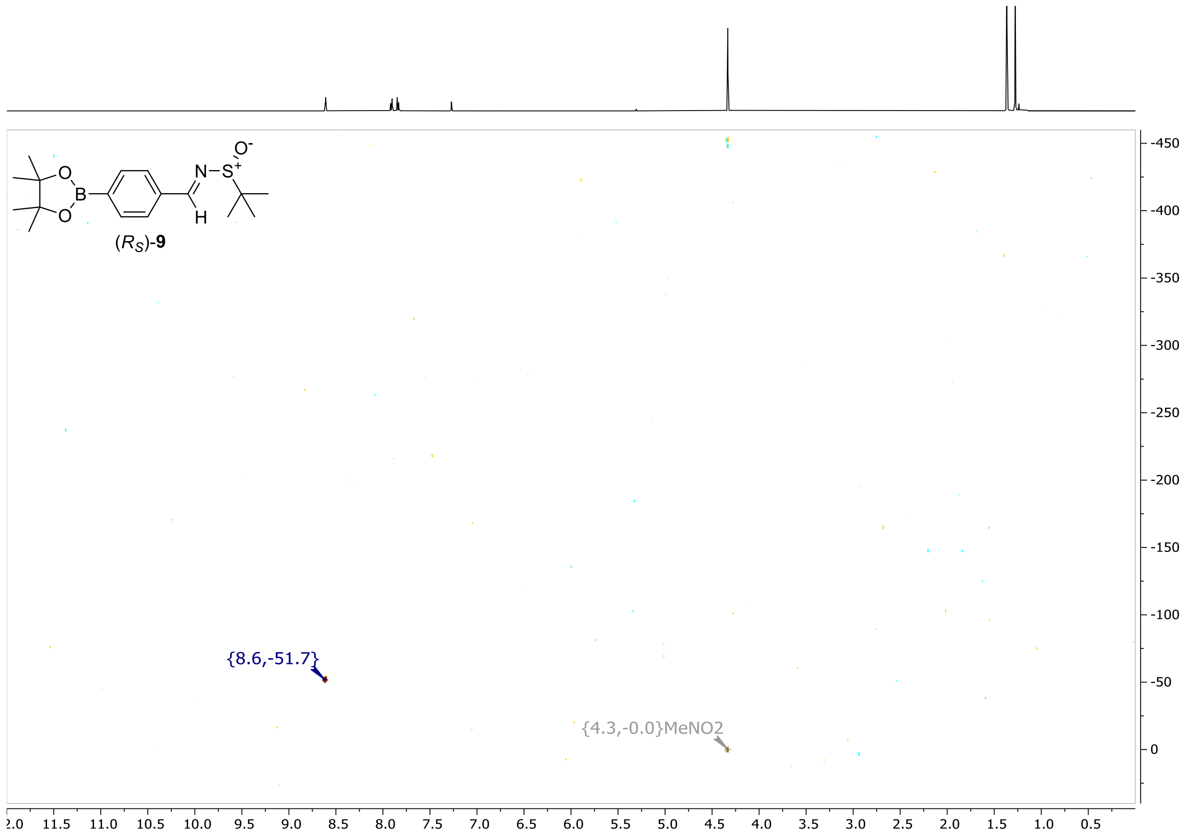

Figure S34: ^{1}H-^{15}N HMBC NMR (500, 51 MHz, $CDCl_3$, 50 mM, ref. 50 mM $MeNO_2$ of (R_S)-**9**.

9 NMR spectra of IBEs, SIBE and SIBA assemblies

9.1 Experimental procedures for multicomponent assemblies

General procedure 2 for the three-component derivatisation of α-methylbenzylamine 1 with 2-FPBA and (*R*)-BINOL – adapted from the procedure of Pérez-Fuertes *et al.*[18]

Enantiopure (*R*)- or (*S*)-α-methylbenzylamine **1** in $CDCl_3$ (1.0 mL, 0.10 M containing ~6 mM TMS internal standard) was added to 2-FPBA (15 mg, 0.10 mmol, 1.0 equiv.) and (*R*)-BINOL (31.5 mg, 0.11 mmol, 1.1 equiv.). The reaction mixture was stirred for 10 min at rt before an aliquot (650 µL) was removed and NMR spectra of the resultant iminoboronate esters **2a**/**2b** acquired.

General procedure 3 for the 'one-pot' stepwise three-component assembly of sulfinamides 3, 2-FPBA and pinanediol. – adapted from the procedure of Groleau *et al.*[1]

2-FPBA (0.12 mmol, 1.2 equiv.) and anhydrous $MgSO_4$ (200 mg) were added to a stirred solution of sulfinamide **3** (0.1 mmol, 1.0 equiv.) in $CDCl_3$ (1.0 mL, ~6 mM TMS internal standard). The reaction was stirred at room temperature for 1 h, before addition of pinanediol (22 mg, 0.13 mmol, 1.3 equiv.). The reaction was then stirred for a further 10 min, before the reaction was filtered, before a 650 µL aliquot was removed and its NMR spectra acquired.

General procedure 4 for the three-component stepwise derivatisation of *tert*-butanesulfinamide 3 with 2-FPBA and BINOL.

Ellman's sulfinamide **3** (1.0 mL, 0.1 M in $CDCl_3$ with ~6 mM TMS) of known enantiopurity was added to a mixture of 2-FPBA (15 mg, 0.10 mmol, 1.0 equiv.) and enantiopure (*R*)- or (*S*)-BINOL (variable amount per sample). The resulting solution was stirred for 1 h at rt, with a 650 µL aliquot then removed and its NMR spectra acquired.

Scalemic and racemic samples of Ellman's sulfinamide **3** were prepared through combination of different amounts of enantiopure solutions of (*R*)- and (*S*)-sulfinamide **3** in $CDCl_3$. Samples required for concentration screening experiments were prepared directly from these 100 mM mixtures by dilution, as required.

^{1}H-^{15}N HMBC spectra were acquired using a 50 mM solution of the desired compound in $CDCl_3$ containing 50 mM $MeNO_2$ as an internal standard.

9.2 ^{1}H, ^{13}C and ^{11}B NMR spectra of IB assemblies

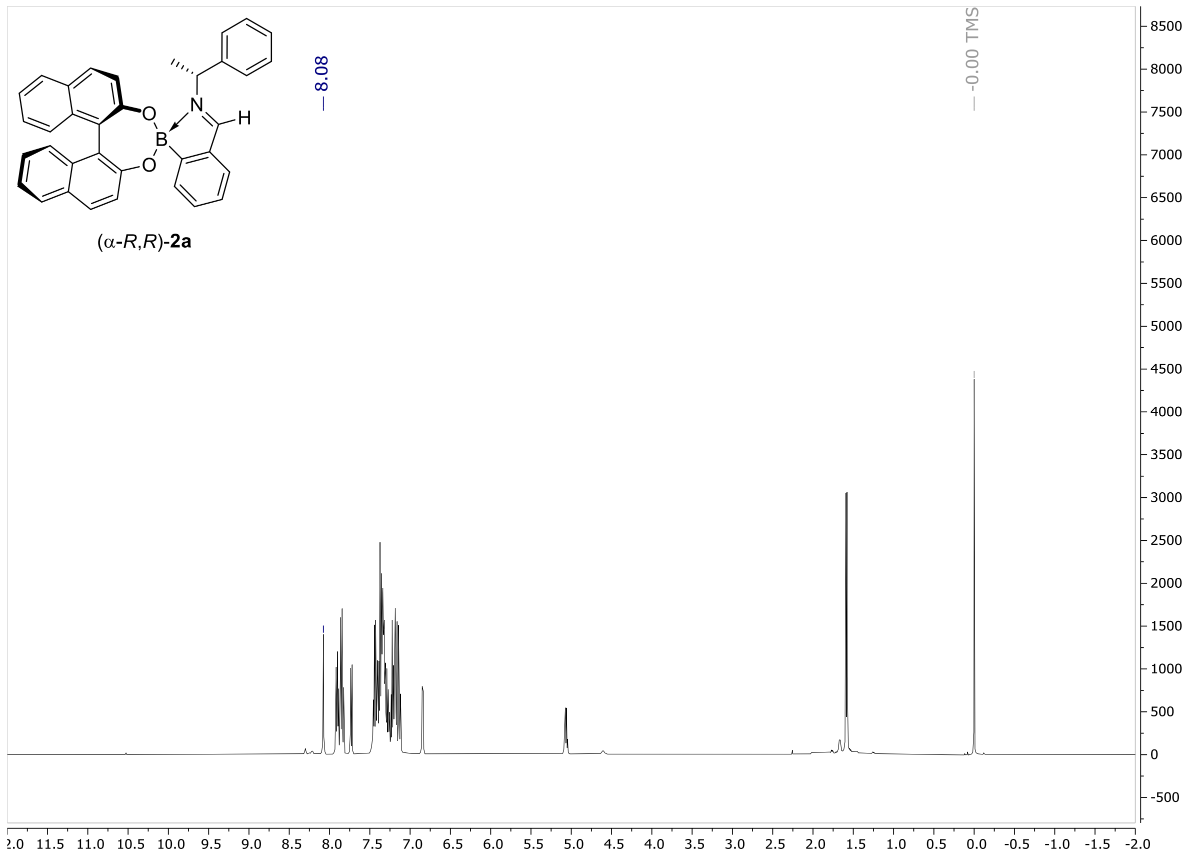

Figure S35: ^{1}H NMR (500 MHz, $CDCl_3$, 100 mM) of (α-*R*,*R*)-**2a** produced following general procedure 2.

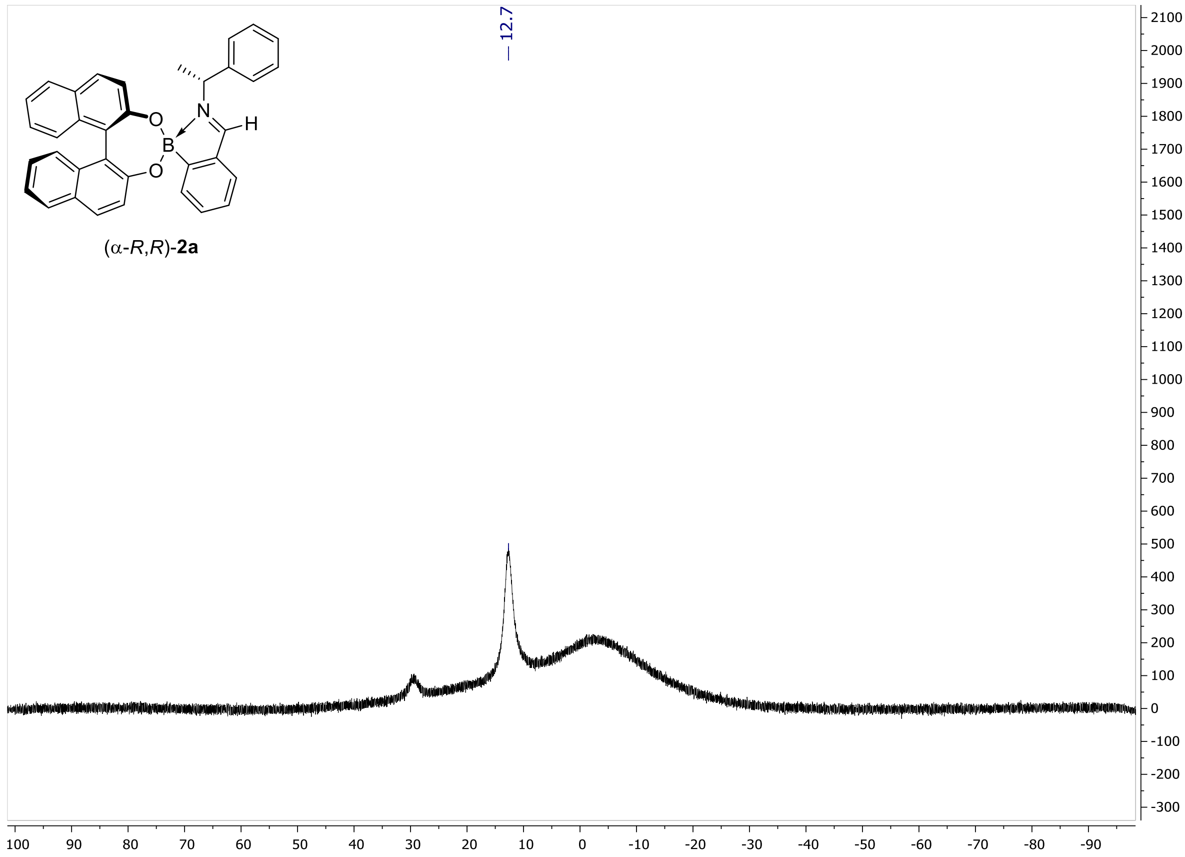

Figure S36: ^{11}B NMR (160 MHz, $CDCl_3$, 100 mM) of (α-*R*,*R*)-**2a** produced following general procedure 2.

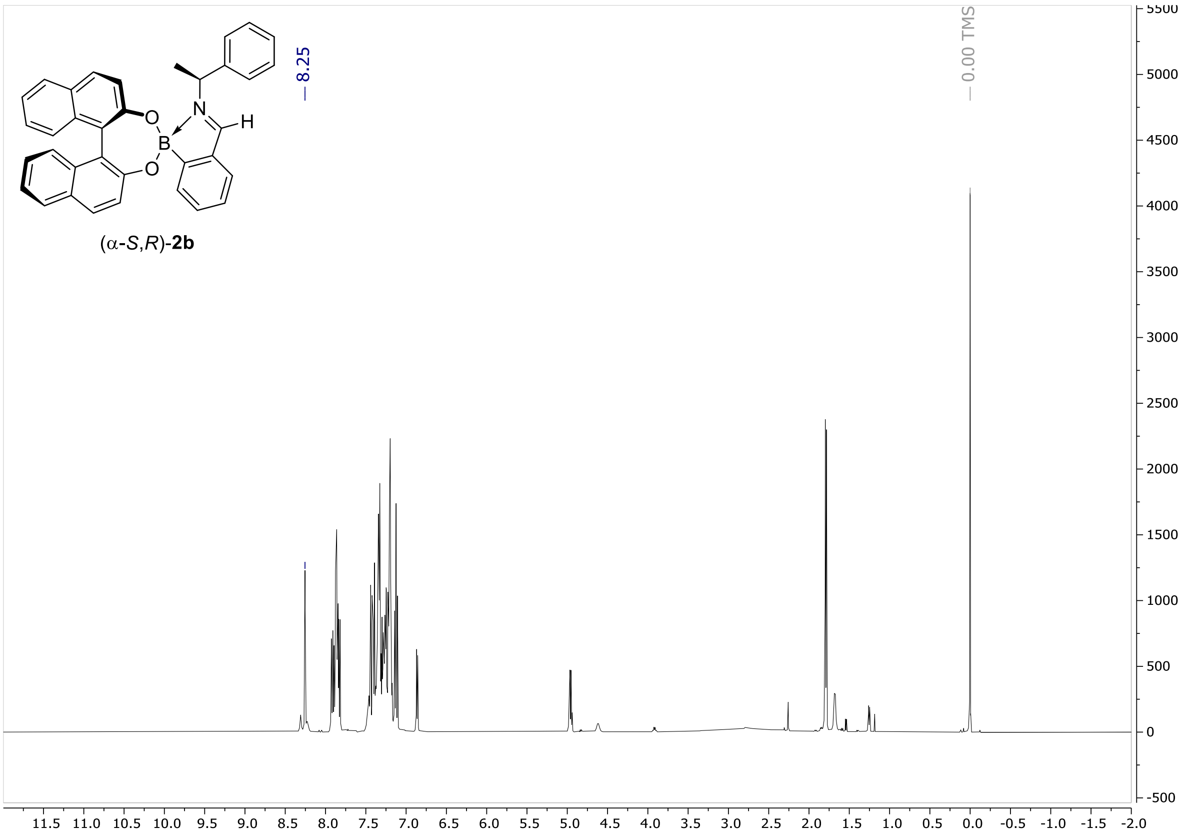

Figure S37: ^{1}H NMR (500 MHz, $CDCl_3$, 100 mM) of (α-*S*,*R*)-**2b** produced following general procedure 2.

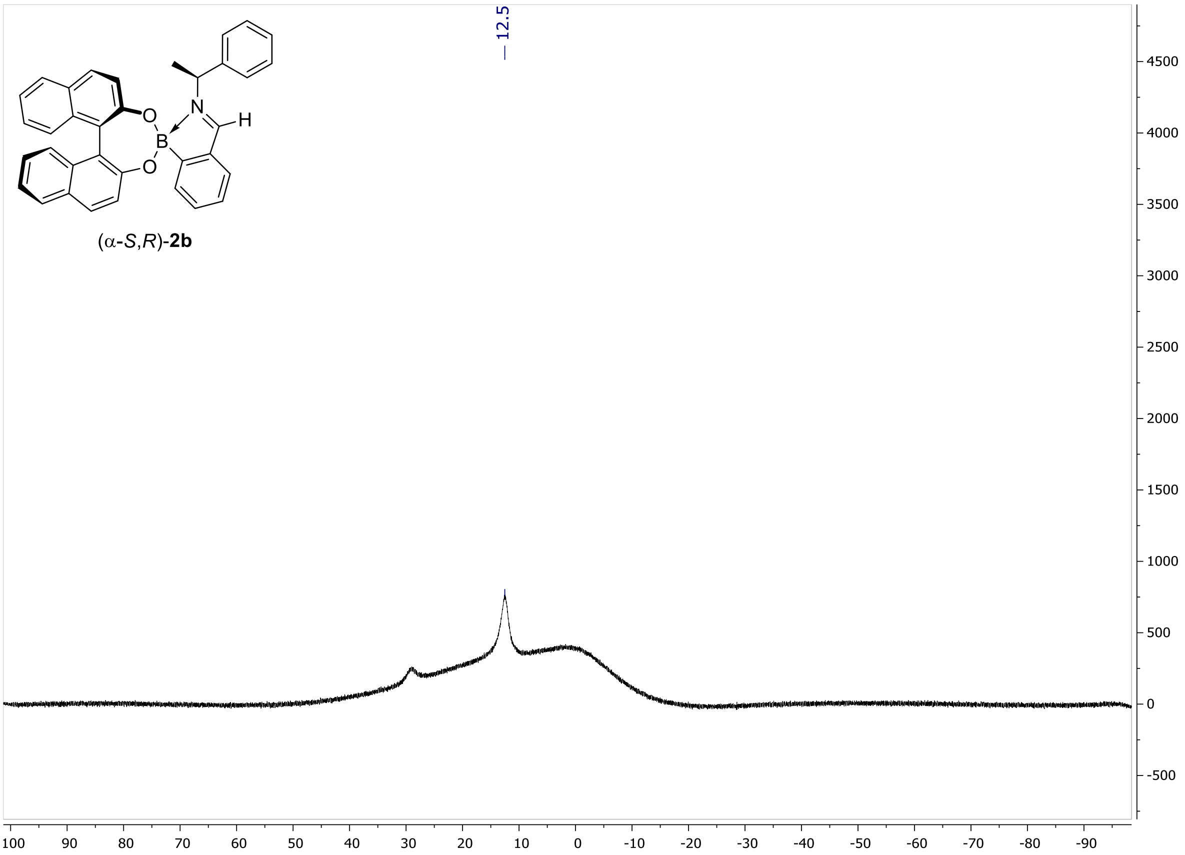

Figure S38: ^{11}B NMR (160 MHz, $CDCl_3$, 100 mM) of (α-*S*,*R*)-**2b** produced following general procedure 2.

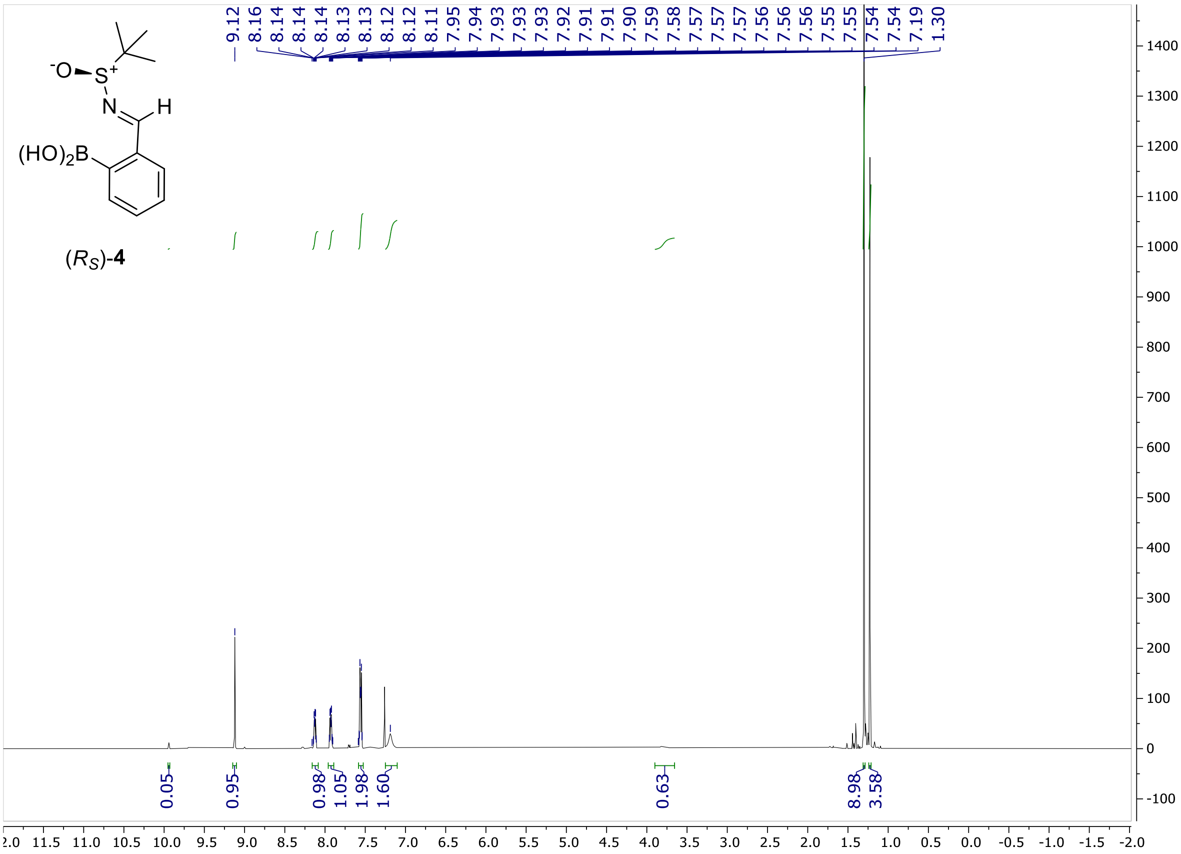

Figure S39: ^{1}H NMR (500 MHz, $CDCl_3$, 100 mM) of (R_S)-**4**.

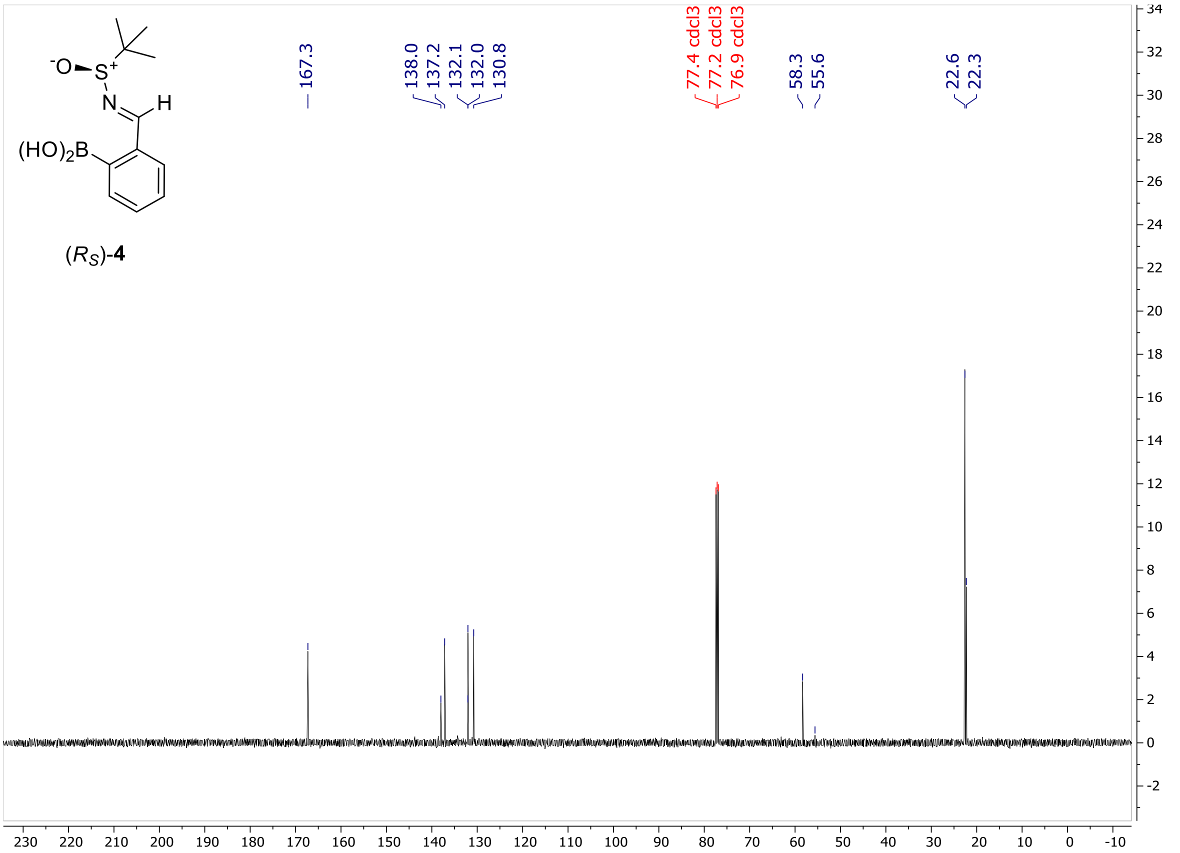

Figure S40: $^{13}C\{^{1}H\}$ NMR (126 MHz, $CDCl_3$, 100 mM) of (R_S)-**4**.

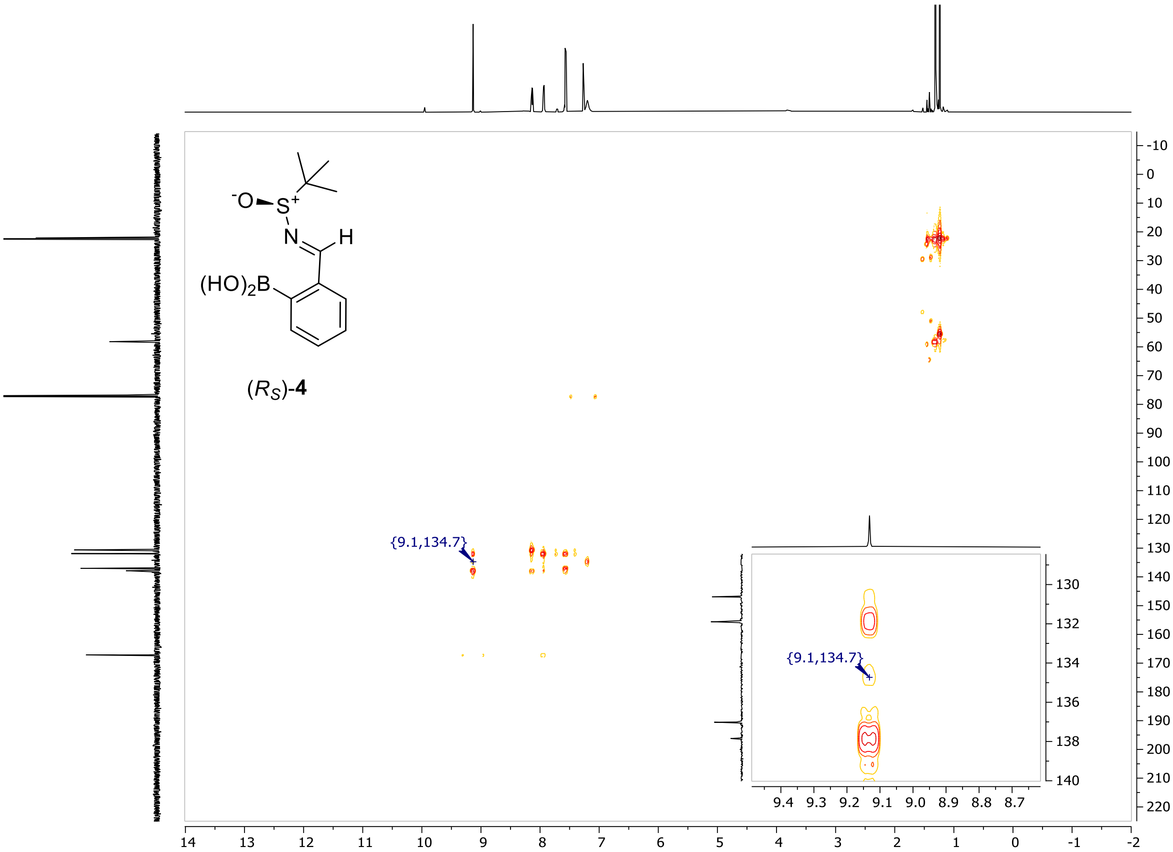

Figure S41: ^{1}H-^{13}C HMBC NMR (500, 126 MHz, $CDCl_3$, 100 mM) of (R_S)-**4**. Ar*H*-C(B) correlations shown.

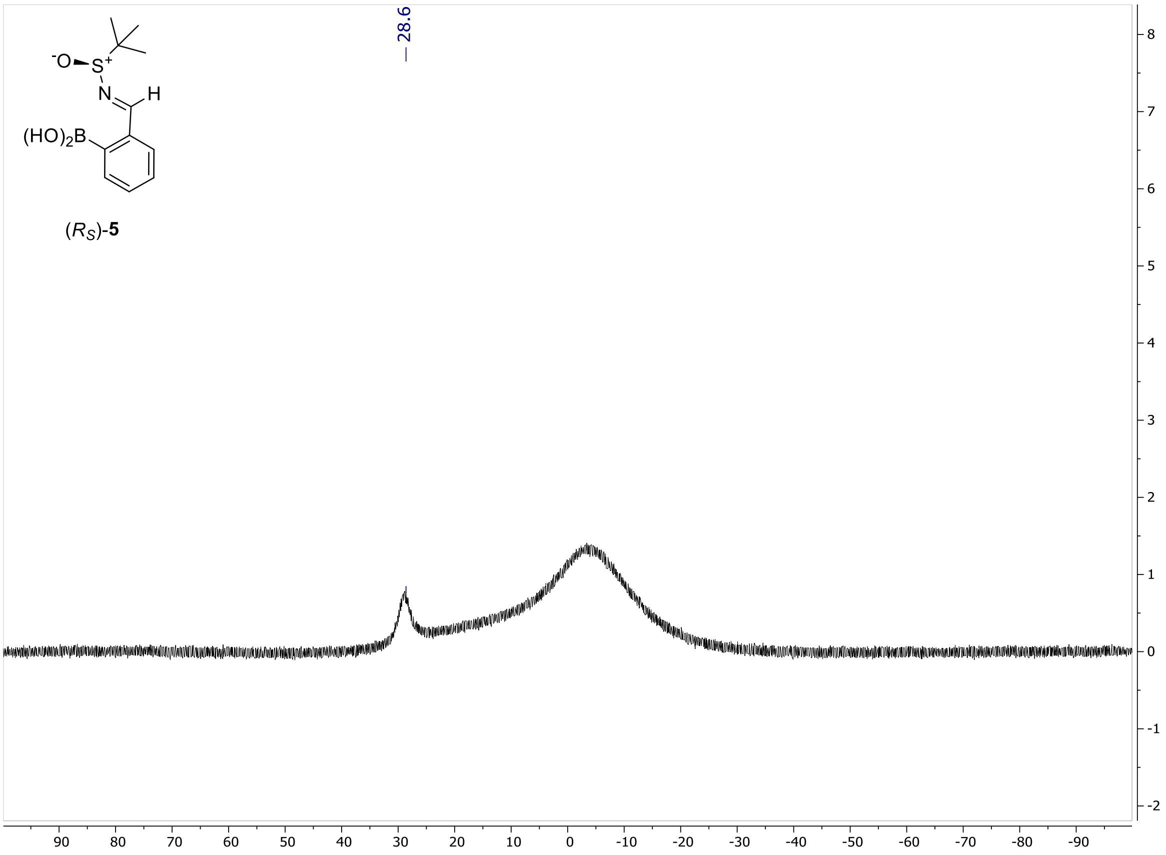

Figure S42: ^{11}B NMR (160 MHz, $CDCl_3$, 100 mM) of (R_S)-**4**.

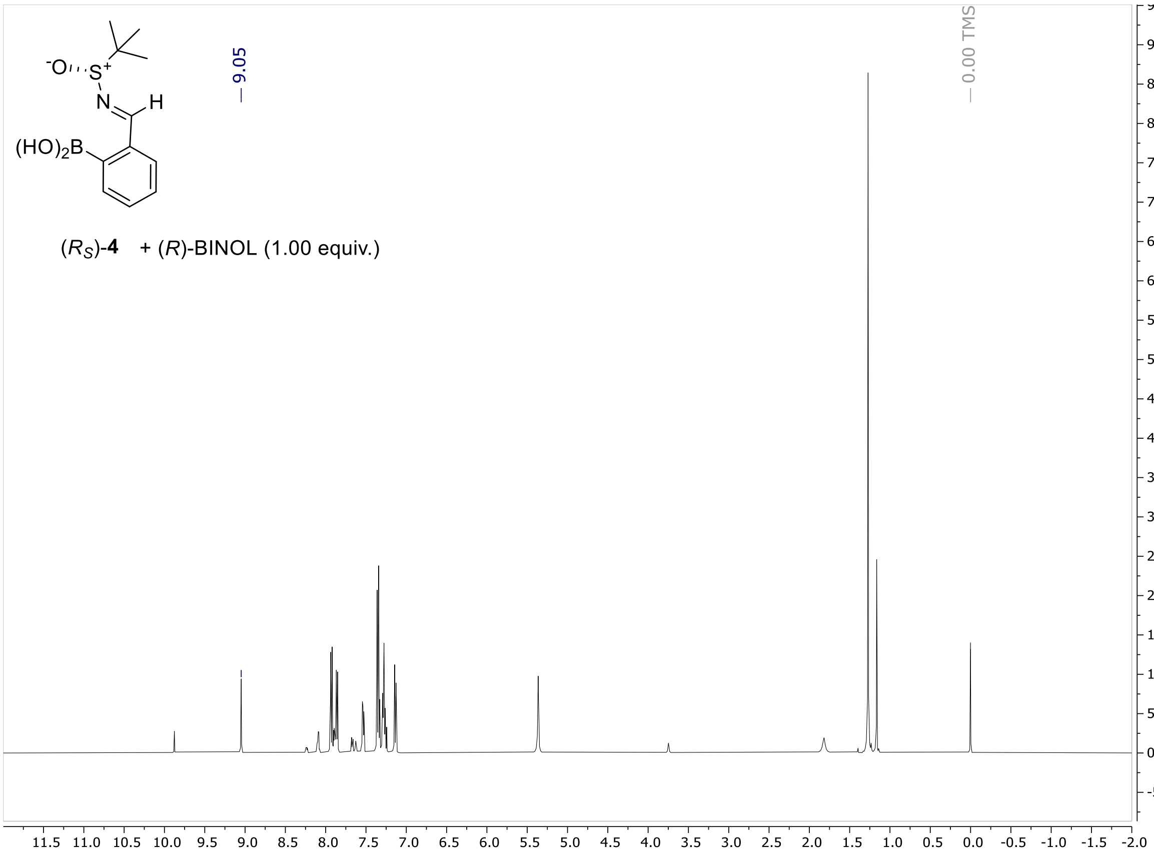

Figure S43: ^{1}H NMR (500 MHz, CDCl$_3$, 100 mM) of (R_S)-**4** + (R)-BINOL (1.00 equiv.) produced following general procedure 4.

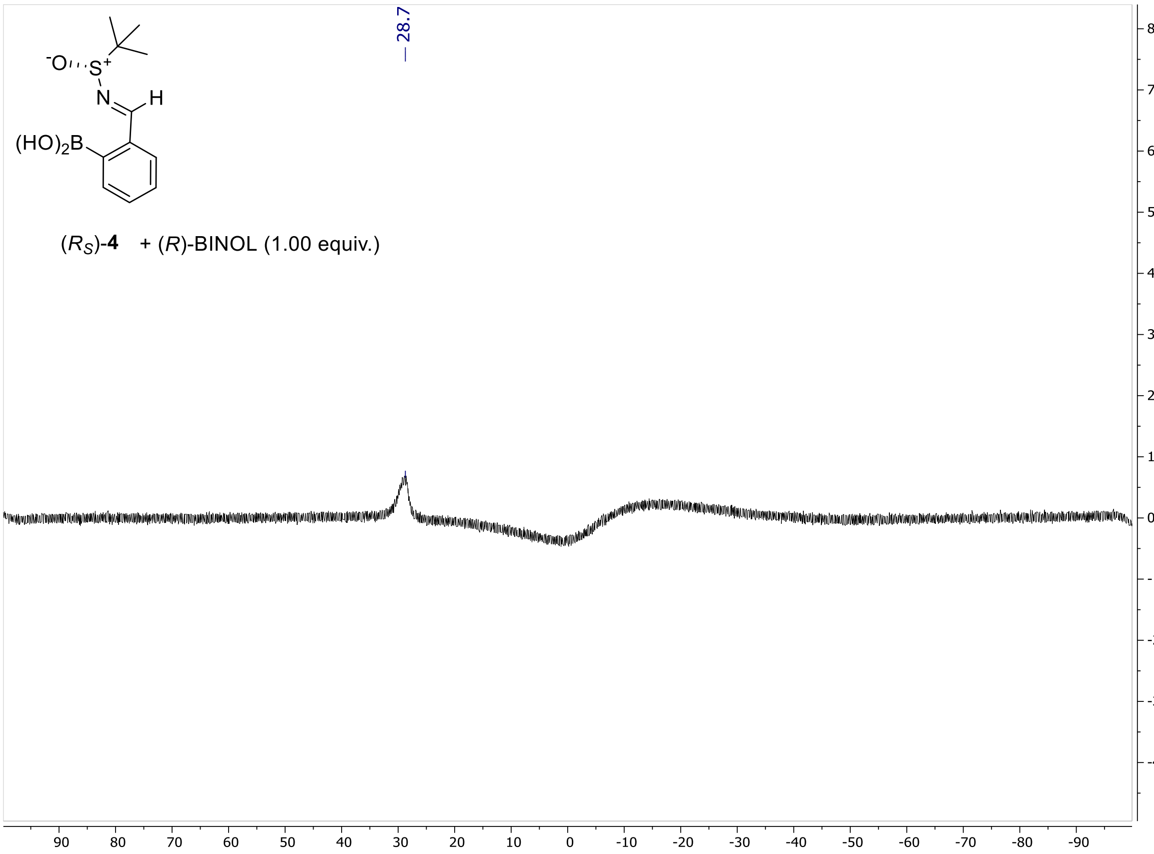

Figure S44: ^{11}B NMR (160 MHz, CDCl$_3$, 100 mM) of (R_S)-**4** + (R)-BINOL (1.00 equiv.) produced following general procedure 4.

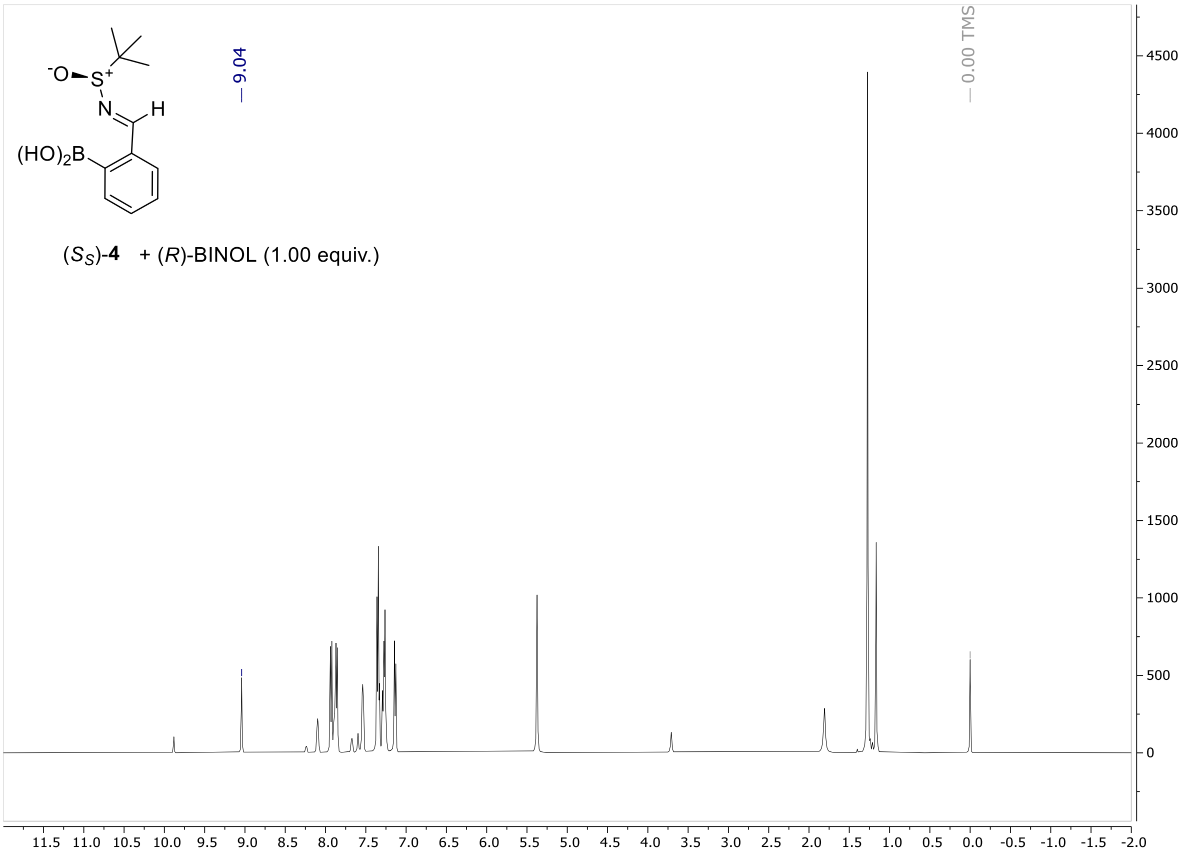

Figure S45: ^{1}H NMR (500 MHz, $CDCl_3$, 100 mM) of (S_S)-**4** + (*R*)-BINOL (1.00 equiv.) produced following general procedure 4.

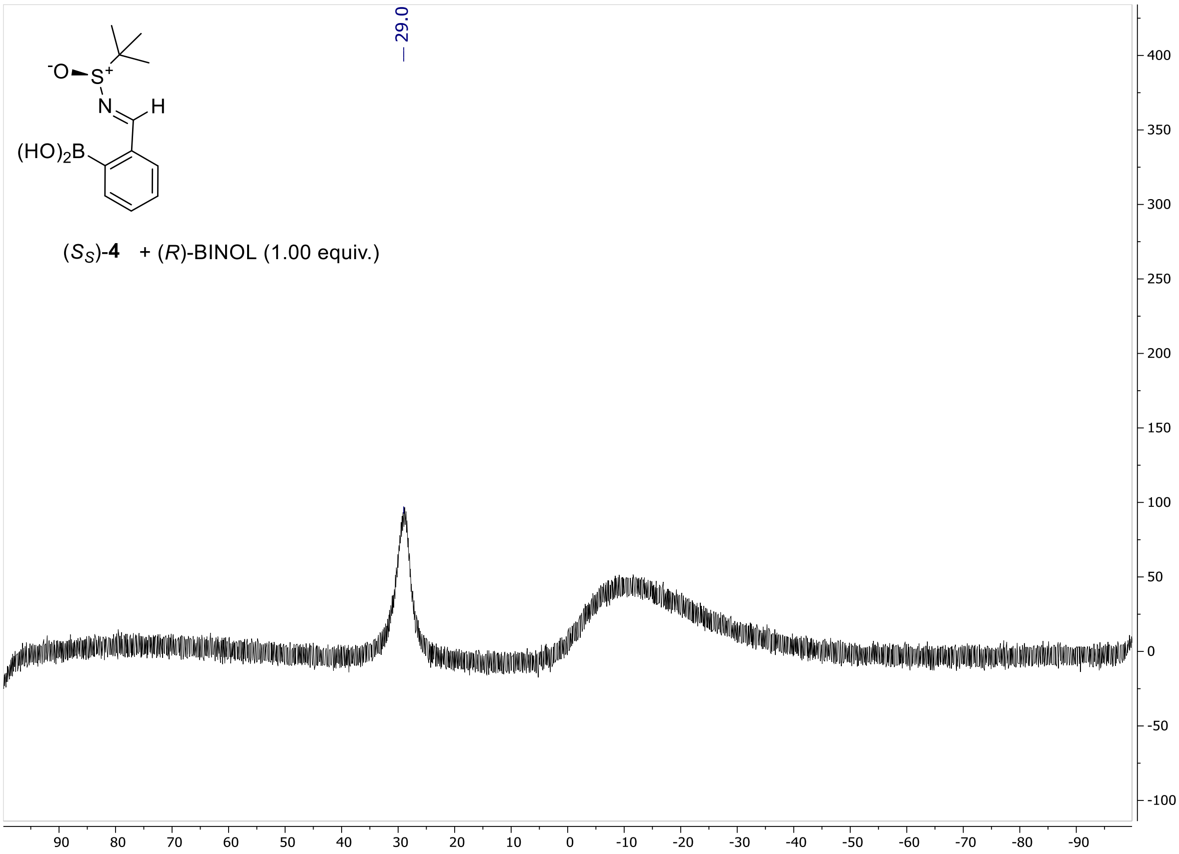

Figure S46: ^{11}B NMR (160 MHz, $CDCl_3$, 100 mM) of (S_S)-**4** + (*R*)-BINOL (1.00 equiv.) produced following general procedure 4.

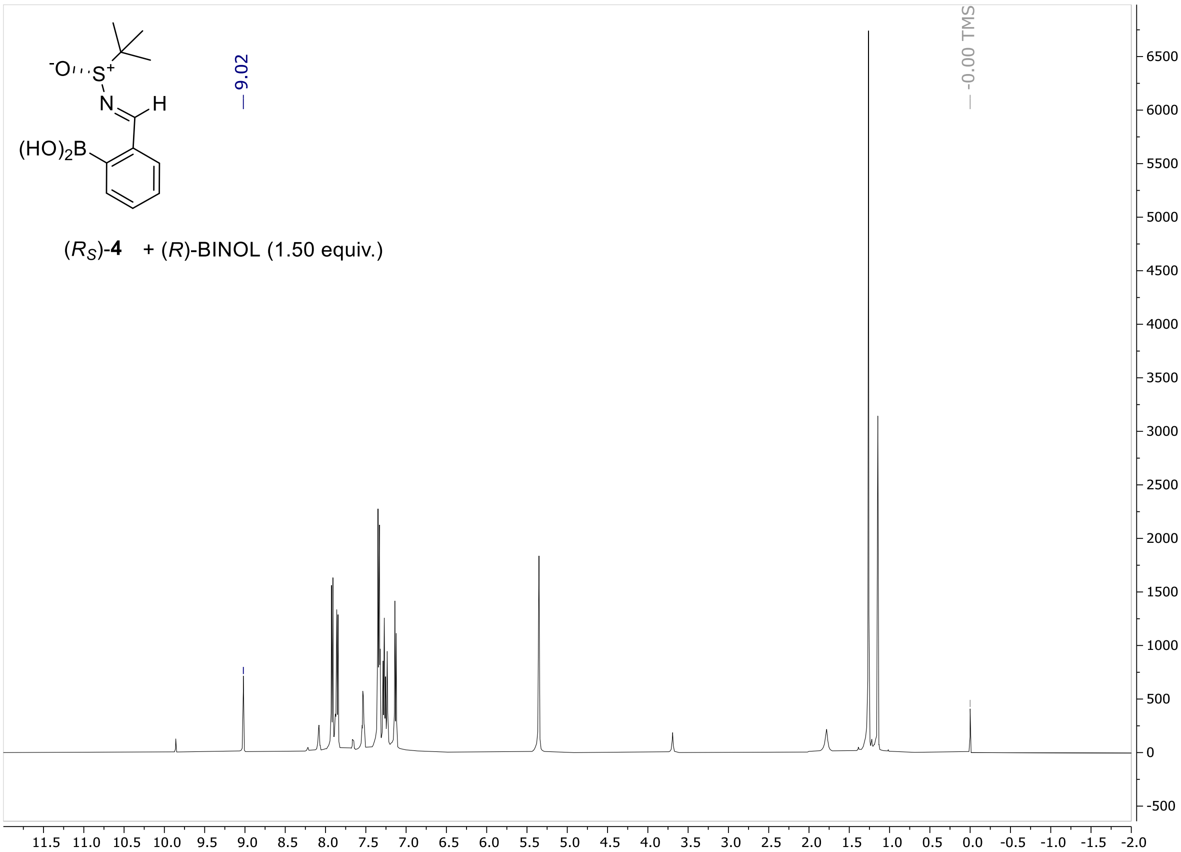

Figure S47: ^{1}H NMR (500 MHz, CDCl$_3$, 100 mM) of (R_S)-**4 +** (R)-BINOL (1.50 equiv.) produced following general procedure 4.

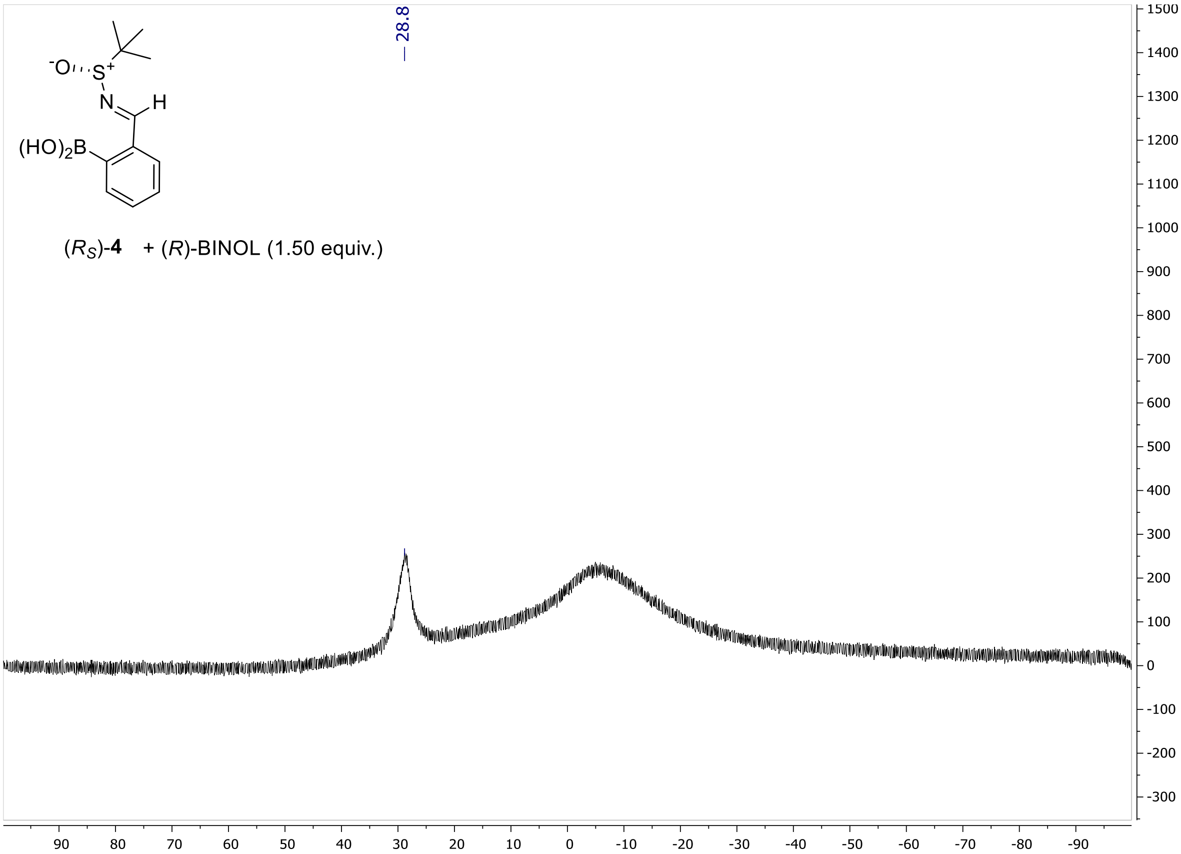

Figure S48: ^{11}B NMR (160 MHz, CDCl$_3$, 100 mM) of (R_S)-**4 +** (R)-BINOL (1.50 equiv.) produced following general procedure 4.

9.3 ^{15}N NMR spectra of IB assemblies

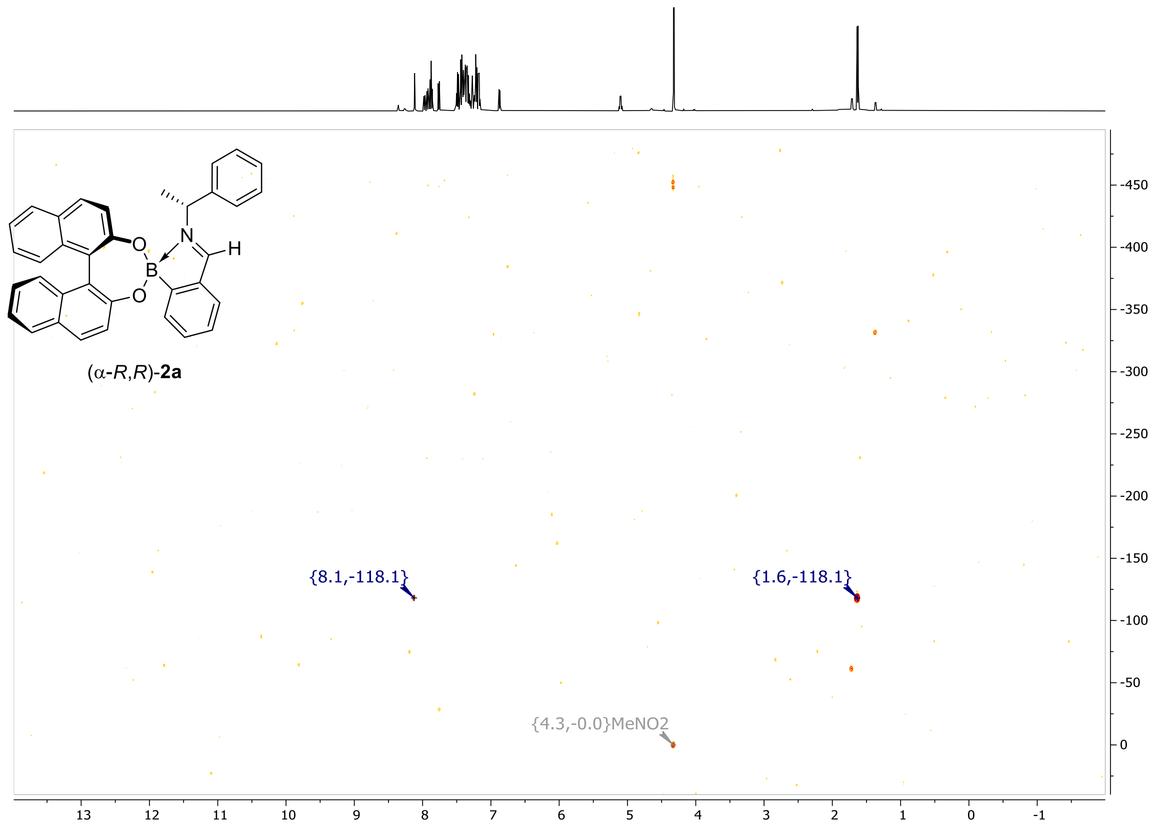

Figure S49: ^{1}H-^{15}N HMBC NMR (500, 51 MHz, $CDCl_3$, 50 mM, ref. 50 mM $MeNO_2$) of (α-*R*,*R*)-**2a** produced following general procedure 2.

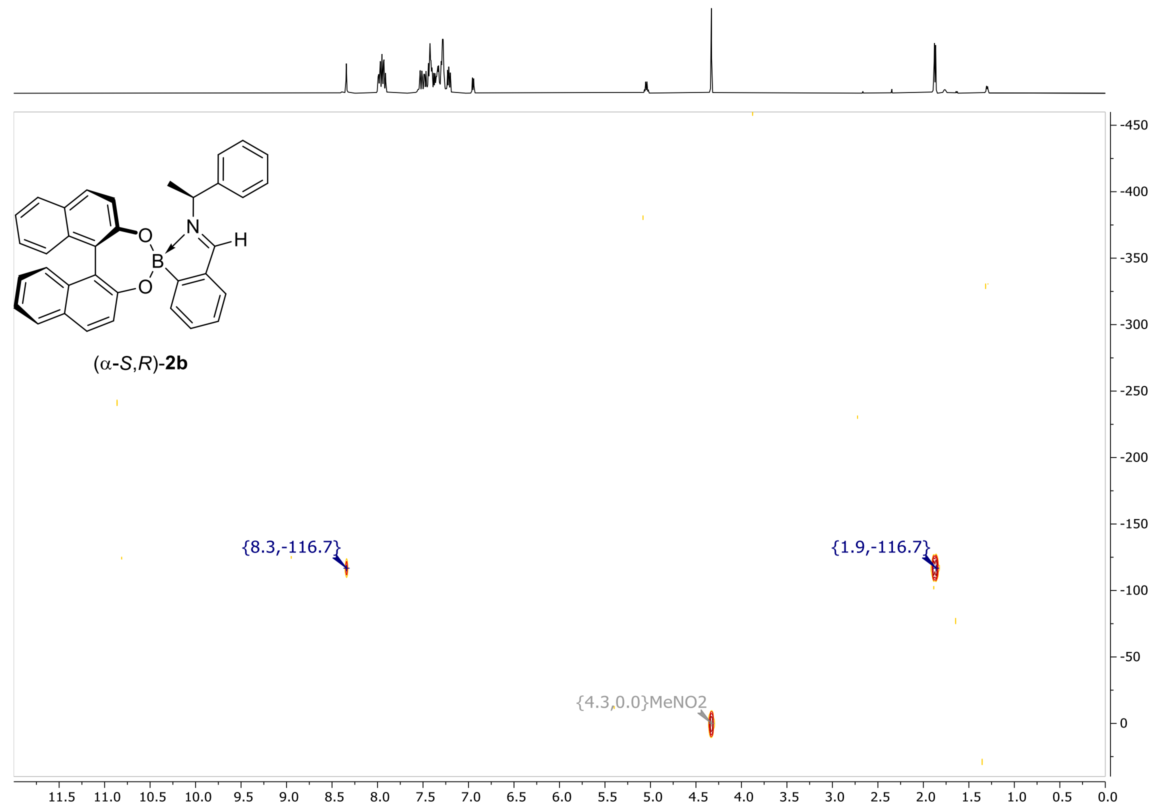

Figure S50: ^{1}H-^{15}N HMBC NMR (500, 51 MHz, $CDCl_3$, 50 mM, ref. 50 mM $MeNO_2$) of (α-*S*,*R*)-**2b** produced following general procedure 2.

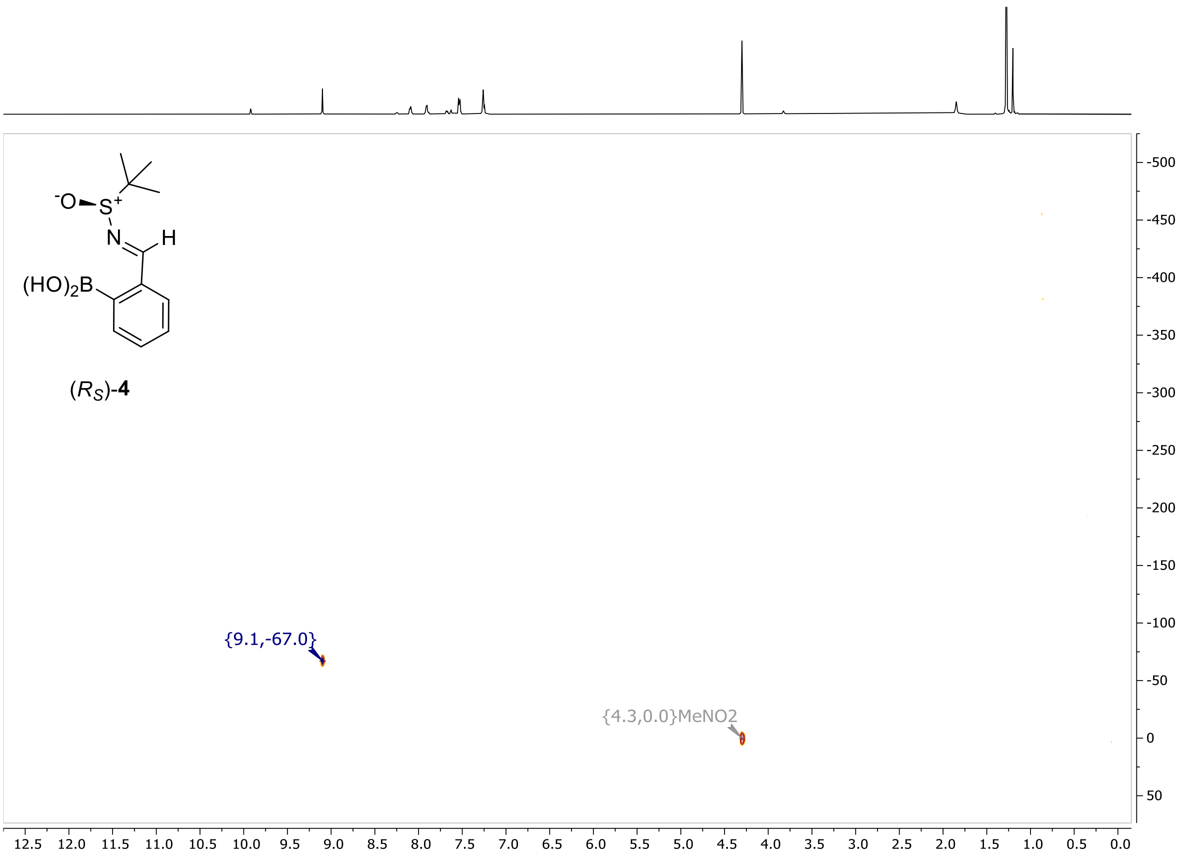

Figure S51: ^{1}H-^{15}N HMBC NMR (500, 51 MHz, $CDCl_3$, 50 mM, ref. 50 mM $MeNO_2$) of (R_S)-**4**.

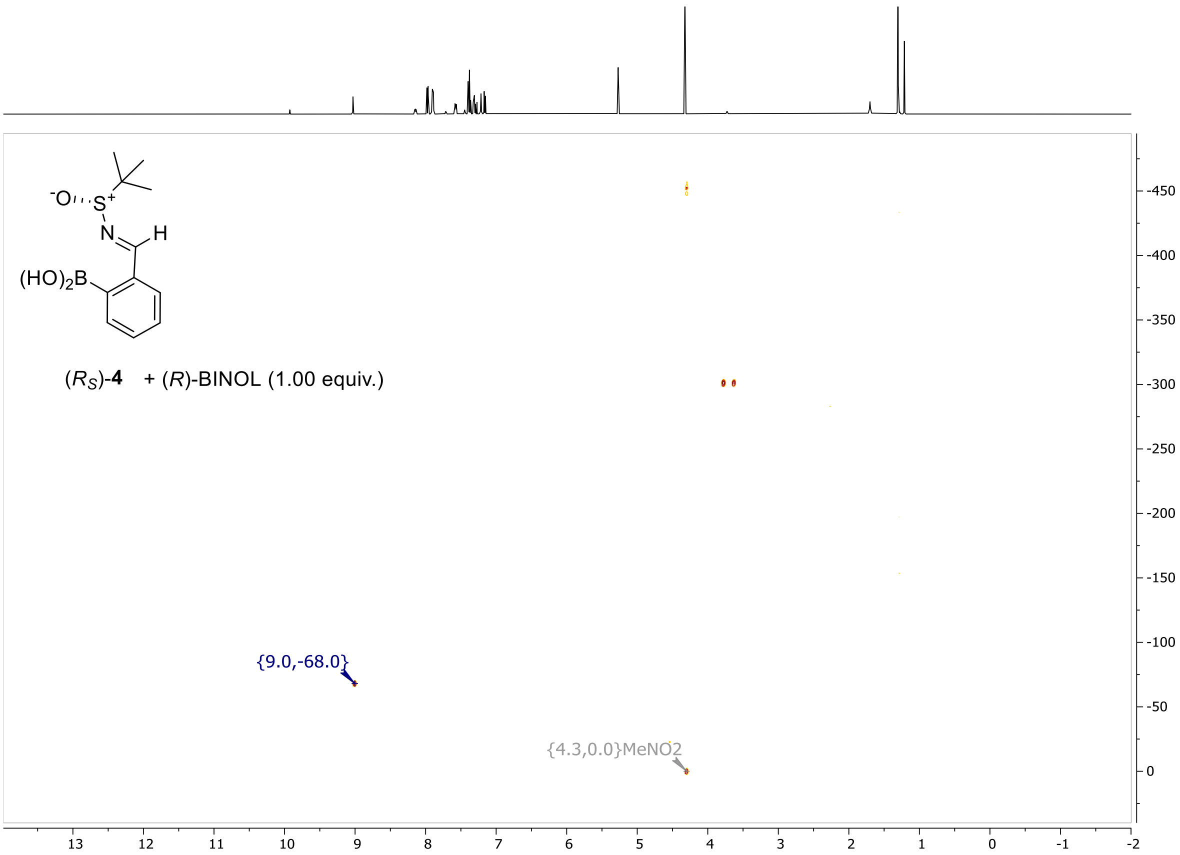

Figure S52: ^{1}H-^{15}N HMBC NMR (500, 51 MHz, $CDCl_3$, 50 mM, ref. 50 mM $MeNO_2$) of (R_S)-**4** + (R)-BINOL (1.00 equiv.) produced following general procedure 4.

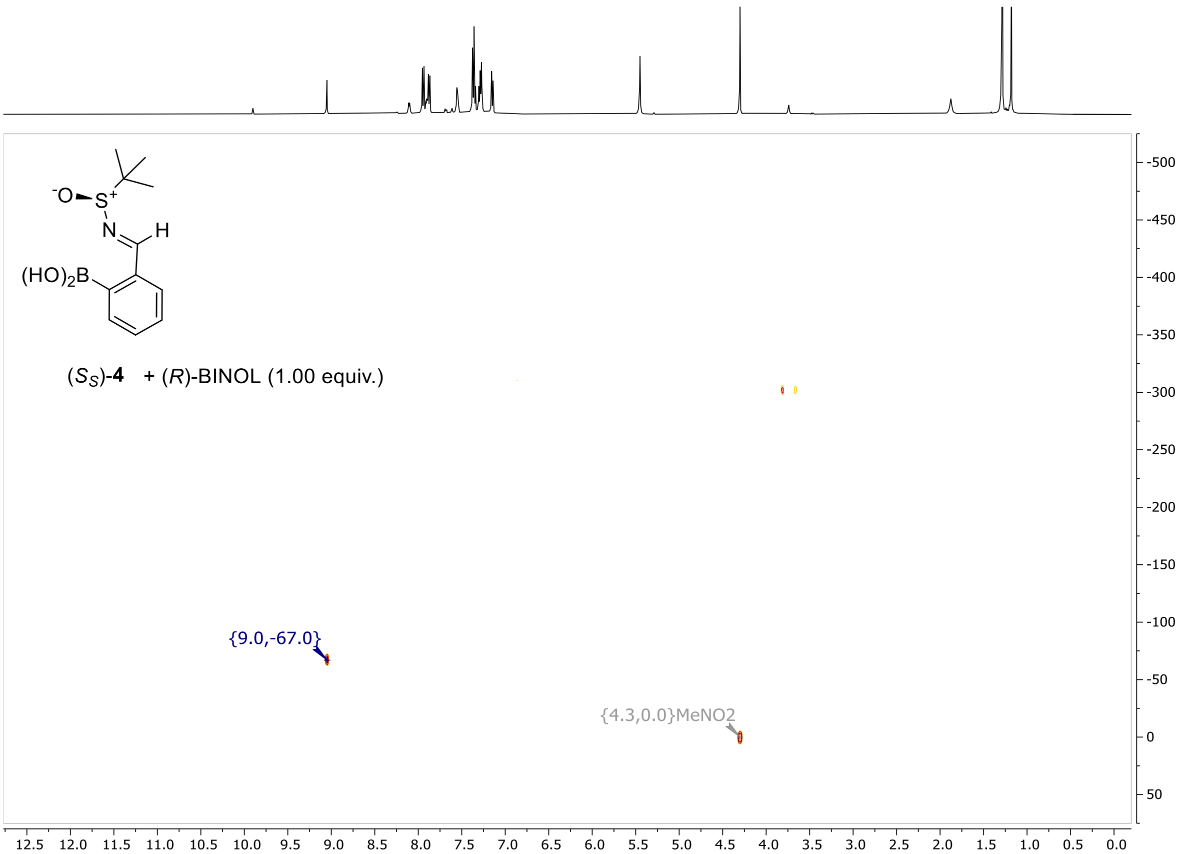

Figure S53: ^{1}H-^{15}N HMBC NMR (500, 51 MHz, $CDCl_3$, 50 mM, ref. 50 mM $MeNO_2$) of (S_S)-**4** + (R)-BINOL (1.00 equiv.) produced following general procedure 4.

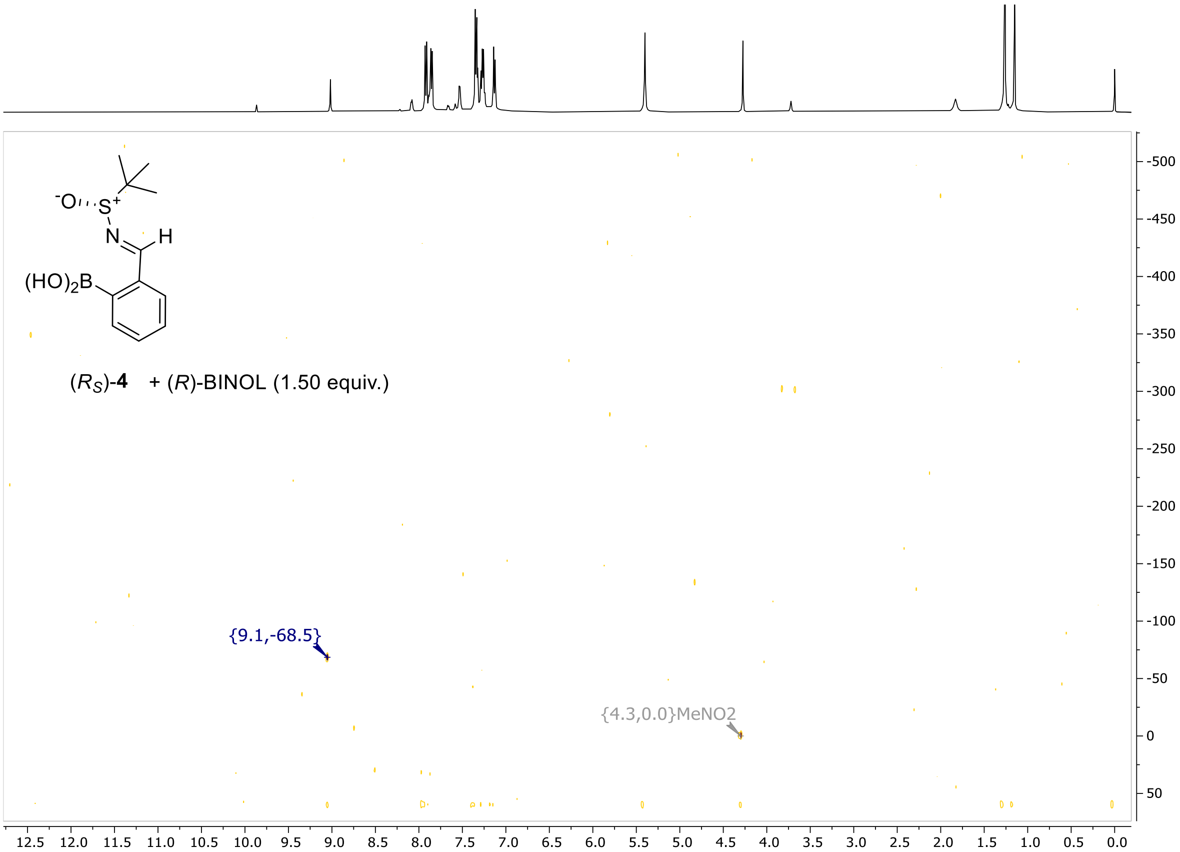

Figure S54: ^{1}H-^{15}N HMBC NMR (500, 51 MHz, $CDCl_3$, 50 mM, ref. 50 mM $MeNO_2$) of (R_S)-**4** + (R)-BINOL (1.50 equiv.) produced following general procedure 4.

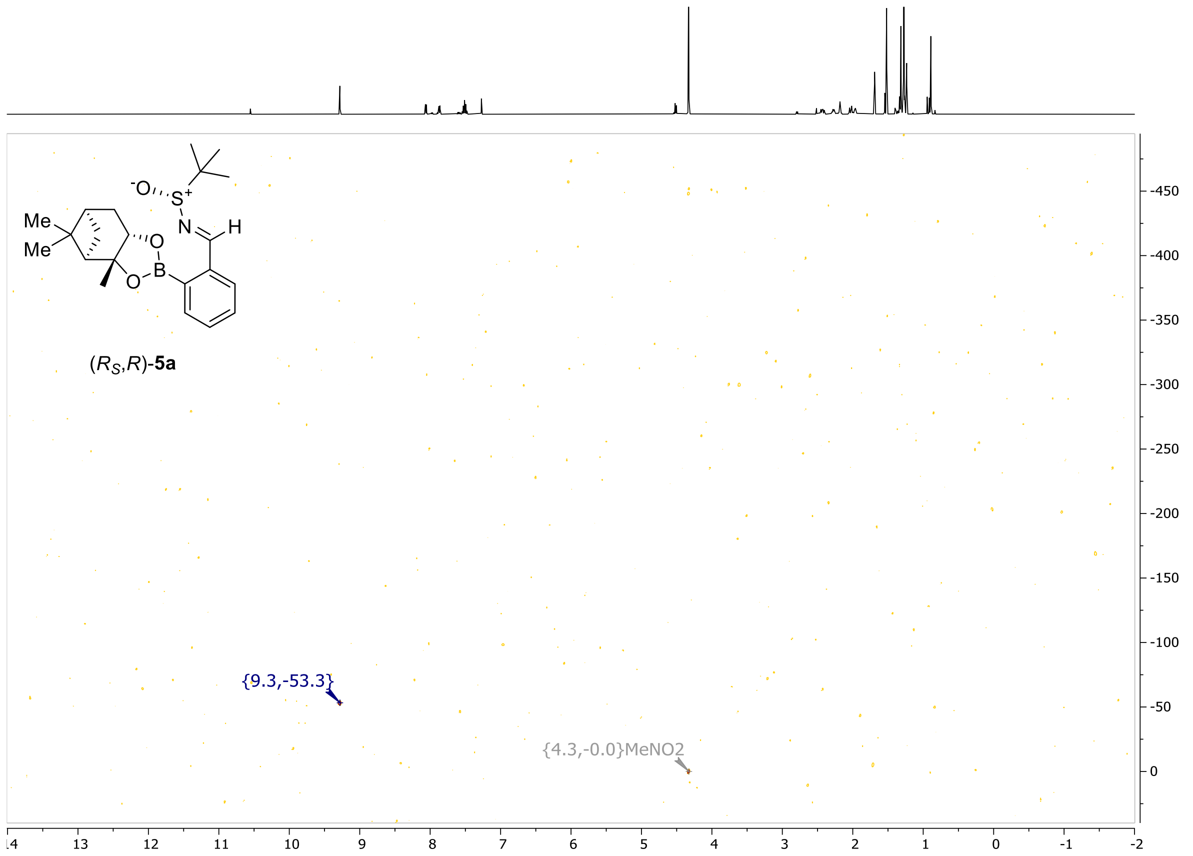

Figure S55: ^{1}H-^{15}N HMBC NMR (500, 51 MHz, $CDCl_3$, 50 mM, ref. 50 mM $MeNO_2$) of (R_S,R)-**5a** produced following general procedure 3.

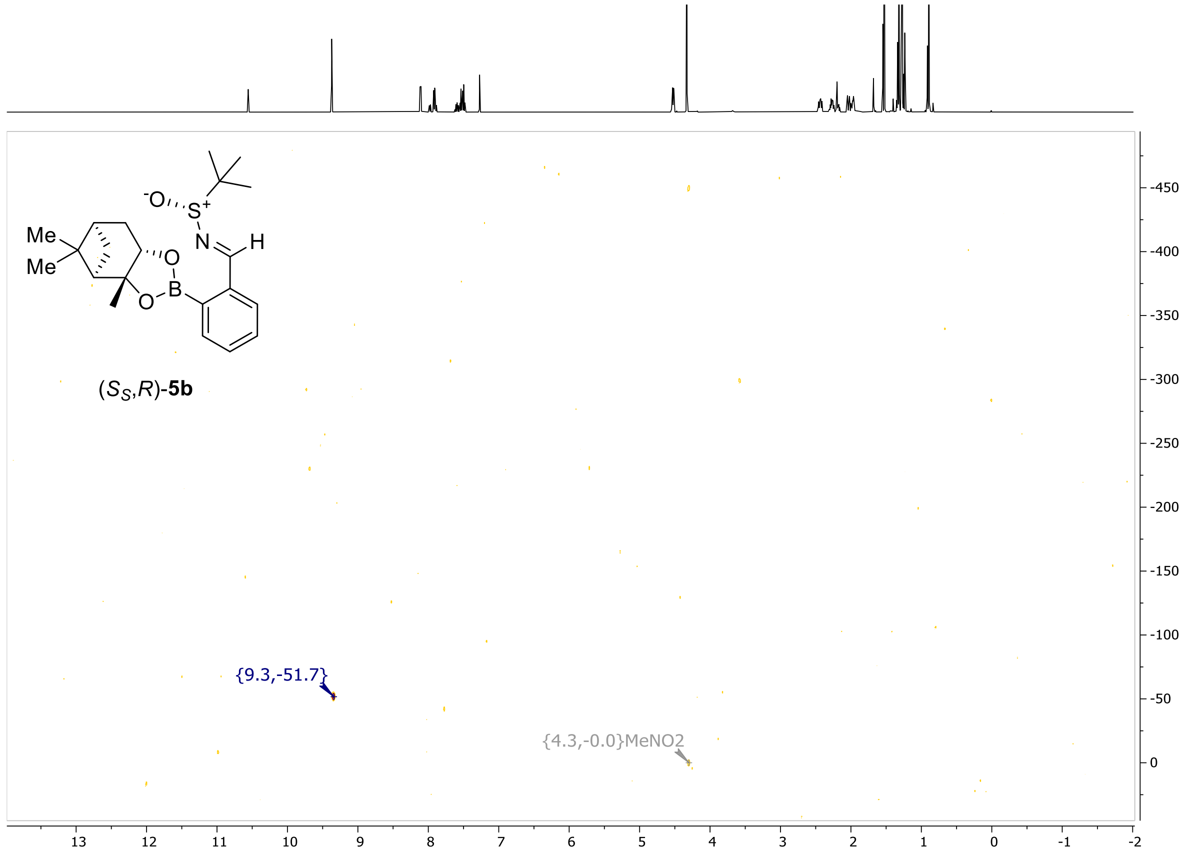

Figure S56: ^{1}H-^{15}N HMBC NMR (500, 51 MHz, $CDCl_3$, 50 mM, ref. 50 mM $MeNO_2$) of (S_S,R)-**5b** produced following general procedure 3.

9.4 1D NOESY NMR spectra of (R_S)-**4** and (*R*)-BINOL

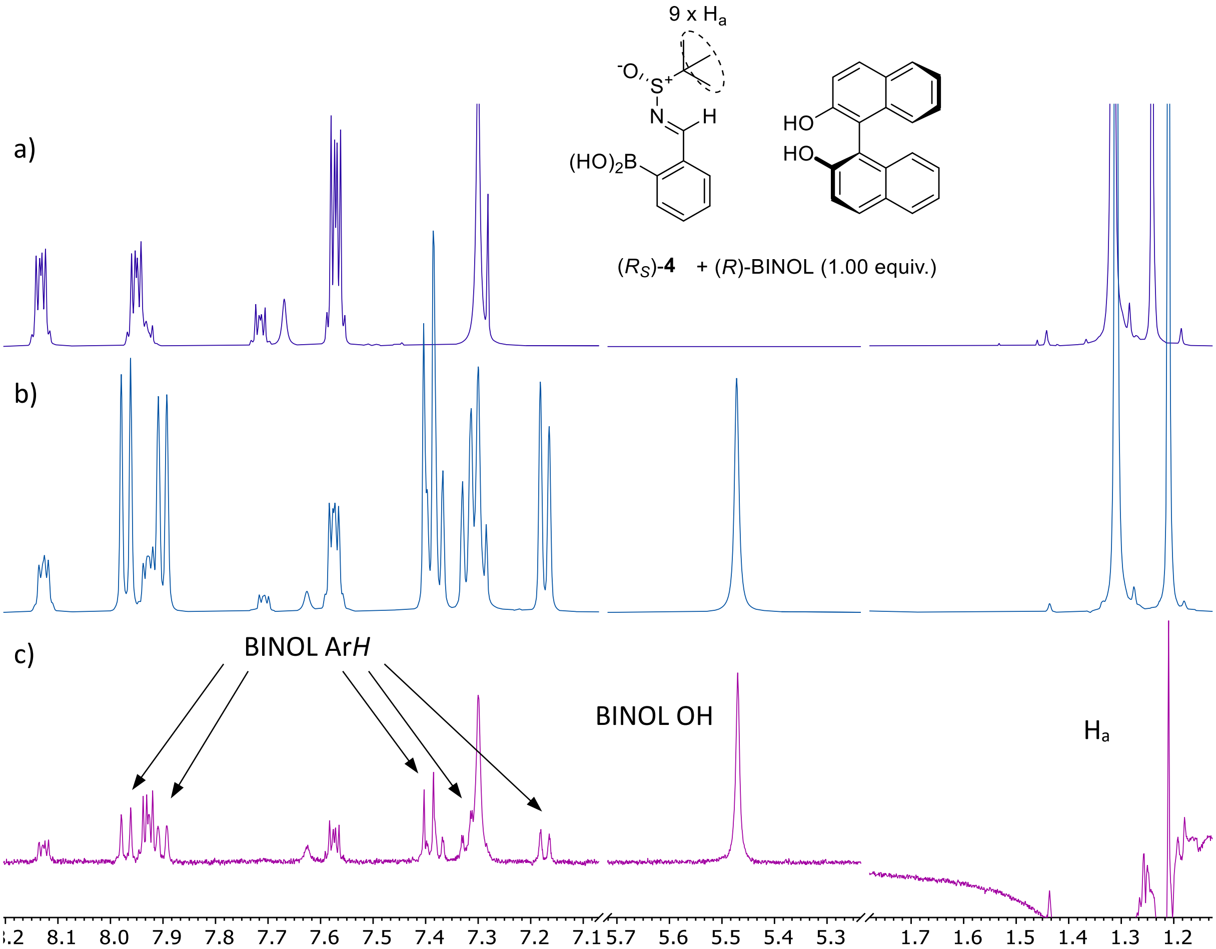

Figure S57: Expanded aromatic region: (a) ^{1}H NMR (500 MHz, $CDCl_3$, 100 mM) of (R_S)-**4**. (b) ^{1}H NMR (500 MHz, $CDCl_3$, 100 mM) of (R_S)-**4** + (*R*)-BINOL (1.00 equiv.). (c) 1D NOE spectrum of sample (b), with selected irradiation of (R_S)-**4** resonances for H_a (1.32 ppm, phased negatively) and NOE interactions in the opposite phase. Intermolecular interactions between Ha and BINOL are highlighted/labelled. b) and c)Acquired on a 500 MHz Bruker Avance III with a prodigy cryoprobe.

10 X-ray crystallography

10.1 Structure of previously-reported IBEs

The structures of IBEs (α-*S*,*S*)-**2a** and (α-*S*,*R*)-**2b** have been previously reported,[19–22] however their crystal structures demonstrating strong N-B bonds are shown again here for comparative purposes.

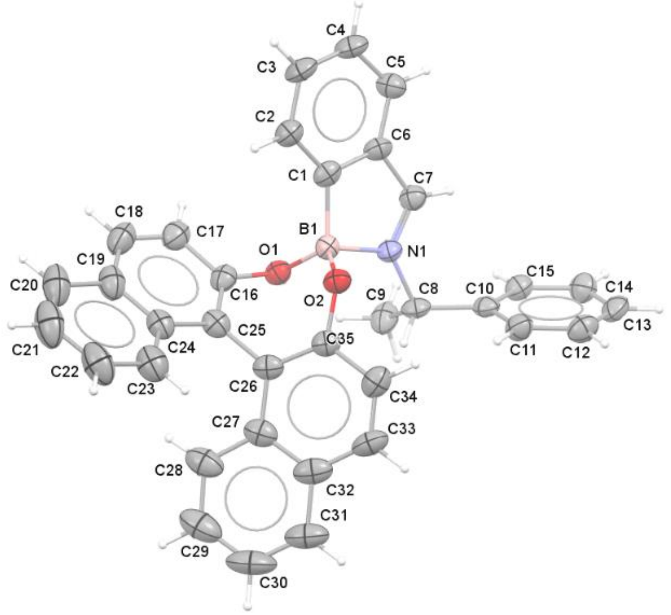

Figure S58: X-ray crystal structure of (α-*S*,*S*)-**2b** showing a strong N→B coordination bond.[21]

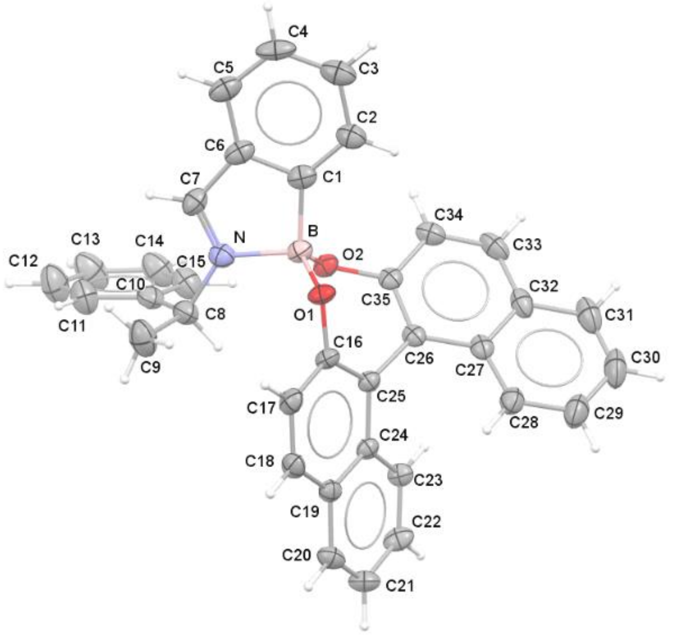

Figure S59: X-ray crystal structure of (α-*S*,*R*)-**2a** showing a strong N→B coordination bond.[22]

*10.2 X-ray crystal structure of SIBAs **4***

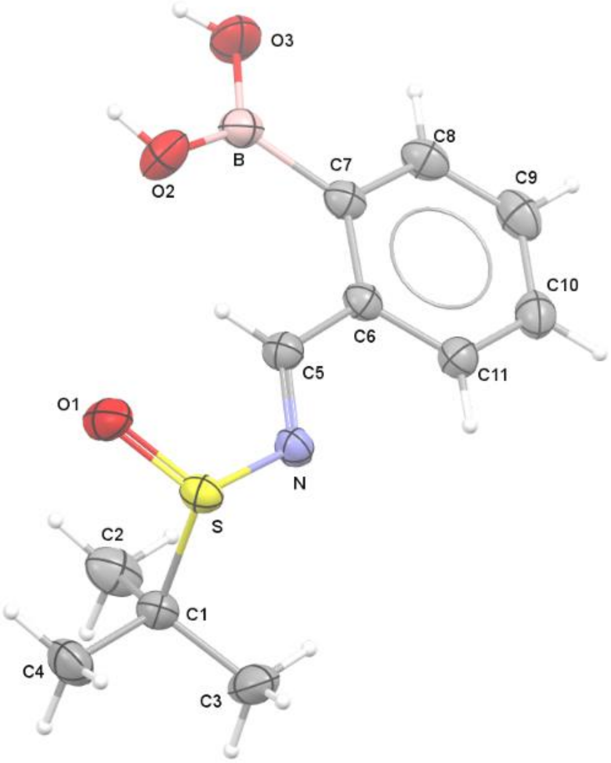

Figure S60: X-ray crystal structure of SIBAs (S_S)-**4**

Crystals suitable for X-ray diffraction were obtained by recrystallisation by slow evaporation of an enantiopure mixture of (S_S)-**4** (*vide supra* for procedure) from 1:1 mixture of $CDCl_3$/*n*-hexane. The crystallographic data in CIF format has been deposited with CCDC: deposition number 2159020.

Table 1. Crystal data and structure refinement for (S_S)-**4**.

Empirical formula	$C_{11}H_{16}BNO_3S$	
Formula weight	253.12	
Temperature	150.00(10) K	
Wavelength	1.54184 Å	
Crystal system	Orthorhombic	
Space group	$P2_12_12_1$	
Unit cell dimensions	a = 9.60006(9) Å	α= 90°.
	b = 11.60553(15) Å	β= 90°.
	c = 11.64615(12) Å	γ = 90°.
Volume	1297.54(2) $Å^3$	
Z	4	
Density (calculated)	1.296 Mg/m^3	

Absorption coefficient	2.187 mm^{-1}
F(000)	536
Crystal size	0.389 x 0.236 x 0.119 mm^3
Theta range for data collection	5.381 to 73.698°.
Index ranges	-11<=h<=11, -14<=k<=11, -14<=l<=14
Reflections collected	24769
Independent reflections	2623 [R(int) = 0.0286]
Completeness to theta = 67.684°	100.0 %
Absorption correction	Gaussian
Max. and min. transmission	1.000 and 0.284
Refinement method	Full-matrix least-squares on F^2
Data / restraints / parameters	2623 / 0 / 165
Goodness-of-fit on F^2	1.066
Final R indices [I>2sigma(I)]	R1 = 0.0230, wR2 = 0.0615
R indices (all data)	R1 = 0.0232, wR2 = 0.0617
Absolute structure parameter	-0.002(5)
Extinction coefficient	n/a
Largest diff. peak and hole	0.226 and -0.230 e.Å^{-3}

Table 2. Atomic coordinates (x 10^4) and equivalent isotropic displacement parameters (Å^{2}x 10^3) for (S_S)-**4**. U(eq) is defined as one third of the trace of the orthogonalized U^{ij} tensor.

	x	y	z	U(eq)
C(1)	4491(2)	2551(2)	3772(2)	28(1)
C(2)	5651(2)	1662(2)	3703(2)	43(1)
C(3)	4459(2)	3181(2)	4918(2)	42(1)
C(4)	3074(2)	2021(2)	3501(2)	40(1)
S	4746(1)	3640(1)	2643(1)	27(1)
O(1)	4864(2)	2991(1)	1542(1)	41(1)
N	6323(2)	4113(1)	3065(1)	27(1)
C(5)	7313(2)	4047(1)	2344(2)	26(1)
C(6)	8712(2)	4489(1)	2616(2)	24(1)
C(7)	9849(2)	4303(1)	1868(2)	26(1)
B	9805(2)	3613(2)	693(2)	30(1)
O(2)	8588(2)	3250(1)	242(1)	38(1)
O(3)	11054(2)	3421(2)	183(2)	53(1)

C(8)	11141(2)	4750(2)	2202(2)	32(1)
C(9)	11311(2)	5358(2)	3211(2)	37(1)
C(10)	10187(2)	5538(2)	3934(2)	34(1)
C(11)	8894(2)	5109(2)	3638(2)	29(1)

Table 3. Bond lengths [Å] for (S_S)-**4**.

C(1)-C(2)	1.520(3)
C(1)-C(3)	1.522(3)
C(1)-C(4)	1.526(3)
C(1)-S	1.8398(18)
C(2)-H(2A)	0.9800
C(2)-H(2B)	0.9800
C(2)-H(2C)	0.9800
C(3)-H(3A)	0.9800
C(3)-H(3B)	0.9800
C(3)-H(3C)	0.9800
C(4)-H(4A)	0.9800
C(4)-H(4B)	0.9800
C(4)-H(4C)	0.9800
S-O(1)	1.4912(14)
S-N	1.6837(15)
N-C(5)	1.271(2)
C(5)-C(6)	1.472(2)
C(5)-H(5)	0.9500
C(6)-C(11)	1.402(3)
C(6)-C(7)	1.413(2)
C(7)-C(8)	1.399(3)
C(7)-B	1.586(3)
B-O(2)	1.349(3)
B-O(3)	1.357(2)
O(2)-H(2)	0.82(3)
O(3)-H(3)	0.79(4)
C(8)-C(9)	1.380(3)
C(8)-H(8)	0.9500
C(9)-C(10)	1.385(3)
C(9)-H(9)	0.9500
C(10)-C(11)	1.380(3)
C(10)-H(10)	0.9500
C(11)-H(11)	0.9500

Table 4. Bond angles [°] for (S_S)-**4**.

C(2)-C(1)-C(3)	112.77(17)
C(2)-C(1)-C(4)	111.63(17)
C(3)-C(1)-C(4)	110.92(16)
C(2)-C(1)-S	109.31(13)
C(3)-C(1)-S	107.45(14)
C(4)-C(1)-S	104.32(13)
C(1)-C(2)-H(2A)	109.5
C(1)-C(2)-H(2B)	109.5
H(2A)-C(2)-H(2B)	109.5
C(1)-C(2)-H(2C)	109.5
H(2A)-C(2)-H(2C)	109.5
H(2B)-C(2)-H(2C)	109.5
C(1)-C(3)-H(3A)	109.5
C(1)-C(3)-H(3B)	109.5
H(3A)-C(3)-H(3B)	109.5
C(1)-C(3)-H(3C)	109.5
H(3A)-C(3)-H(3C)	109.5
H(3B)-C(3)-H(3C)	109.5
C(1)-C(4)-H(4A)	109.5
C(1)-C(4)-H(4B)	109.5
H(4A)-C(4)-H(4B)	109.5
C(1)-C(4)-H(4C)	109.5
H(4A)-C(4)-H(4C)	109.5
H(4B)-C(4)-H(4C)	109.5
O(1)-S-N	110.36(8)
O(1)-S-C(1)	106.15(9)
N-S-C(1)	97.76(8)
C(5)-N-S	117.38(13)
N-C(5)-C(6)	121.27(16)
N-C(5)-H(5)	119.4
C(6)-C(5)-H(5)	119.4
C(11)-C(6)-C(7)	120.39(16)
C(11)-C(6)-C(5)	118.39(15)
C(7)-C(6)-C(5)	121.23(16)
C(8)-C(7)-C(6)	117.15(17)

C(8)-C(7)-B	116.78(16)
C(6)-C(7)-B	126.06(16)
O(2)-B-O(3)	122.92(18)
O(2)-B-C(7)	121.16(16)
O(3)-B-C(7)	115.92(17)
B-O(2)-H(2)	112(2)
B-O(3)-H(3)	109(3)
C(9)-C(8)-C(7)	122.08(18)
C(9)-C(8)-H(8)	119.0
C(7)-C(8)-H(8)	119.0
C(8)-C(9)-C(10)	120.16(18)
C(8)-C(9)-H(9)	119.9
C(10)-C(9)-H(9)	119.9
C(11)-C(10)-C(9)	119.65(17)
C(11)-C(10)-H(10)	120.2
C(9)-C(10)-H(10)	120.2
C(10)-C(11)-C(6)	120.58(17)
C(10)-C(11)-H(11)	119.7
C(6)-C(11)-H(11)	119.7

Table 5. Anisotropic displacement parameters ($Å^2$x 10^3) for (S_S)-**4**. The anisotropic displacement factor exponent takes the form: $-2\pi^2[h^2 a^{*2}U^{11} + ... + 2 h k a^* b^* U^{12}]$

	U^{11}	U^{22}	U^{33}	U^{23}	U^{13}	U^{12}
C(1)	27(1)	29(1)	28(1)	0(1)	3(1)	-2(1)
C(2)	38(1)	35(1)	55(1)	13(1)	7(1)	7(1)
C(3)	39(1)	62(1)	27(1)	-6(1)	7(1)	-9(1)
C(4)	32(1)	39(1)	49(1)	1(1)	3(1)	-12(1)
S	21(1)	31(1)	29(1)	2(1)	0(1)	-1(1)
O(1)	33(1)	64(1)	25(1)	-5(1)	-2(1)	-13(1)
N	23(1)	27(1)	32(1)	-2(1)	2(1)	-3(1)
C(5)	25(1)	28(1)	25(1)	1(1)	0(1)	0(1)
C(6)	25(1)	22(1)	27(1)	3(1)	0(1)	0(1)
C(7)	25(1)	24(1)	29(1)	4(1)	2(1)	2(1)
B	31(1)	28(1)	30(1)	2(1)	6(1)	2(1)
O(2)	33(1)	51(1)	30(1)	-12(1)	-2(1)	9(1)
O(3)	37(1)	65(1)	56(1)	-25(1)	21(1)	-11(1)
C(8)	23(1)	36(1)	38(1)	5(1)	2(1)	-1(1)
C(9)	29(1)	38(1)	45(1)	4(1)	-9(1)	-9(1)
C(10)	41(1)	30(1)	32(1)	-1(1)	-5(1)	-6(1)
C(11)	31(1)	27(1)	29(1)	0(1)	2(1)	-1(1)

Table 6. Hydrogen coordinates (x 10^4) and isotropic displacement parameters ($Å^2$x 10^{3}) for (S_S)-**4**.

	x	y	z	U(eq)
H(2A)	5666	1321	2934	64
H(2B)	5488	1057	4275	64
H(2C)	6547	2036	3857	64
H(3A)	5373	3525	5068	64
H(3B)	4231	2635	5532	64
H(3C)	3751	3789	4893	64
H(4A)	2362	2626	3500	60
H(4B)	2843	1444	4084	60
H(4C)	3106	1654	2744	60
H(5)	7148	3705	1616	31
H(2)	8700(30)	2900(30)	-360(30)	69(10)
H(3)	10940(40)	3050(30)	-380(30)	83(12)
H(8)	11925	4632	1719	39
H(9)	12201	5654	3410	45
H(10)	10304	5953	4630	41
H(11)	8121	5236	4131	35

Table 7. Torsion angles [°] for (S_S)-**4**.

C(2)-C(1)-S-O(1)	53.80(16)
C(3)-C(1)-S-O(1)	176.49(13)
C(4)-C(1)-S-O(1)	-65.70(14)
C(2)-C(1)-S-N	-60.09(15)
C(3)-C(1)-S-N	62.60(14)
C(4)-C(1)-S-N	-179.60(13)
O(1)-S-N-C(5)	11.65(17)
C(1)-S-N-C(5)	122.14(15)
S-N-C(5)-C(6)	177.10(12)
N-C(5)-C(6)-C(11)	-7.4(2)
N-C(5)-C(6)-C(7)	172.95(16)
C(11)-C(6)-C(7)-C(8)	0.7(3)
C(5)-C(6)-C(7)-C(8)	-179.66(15)
C(11)-C(6)-C(7)-B	179.52(16)
C(5)-C(6)-C(7)-B	-0.8(3)
C(8)-C(7)-B-O(2)	-173.26(18)
C(6)-C(7)-B-O(2)	7.9(3)
C(8)-C(7)-B-O(3)	6.6(3)
C(6)-C(7)-B-O(3)	-172.26(18)
C(6)-C(7)-C(8)-C(9)	-0.6(3)
B-C(7)-C(8)-C(9)	-179.52(17)
C(7)-C(8)-C(9)-C(10)	0.4(3)
C(8)-C(9)-C(10)-C(11)	-0.3(3)
C(9)-C(10)-C(11)-C(6)	0.4(3)
C(7)-C(6)-C(11)-C(10)	-0.6(3)
C(5)-C(6)-C(11)-C(10)	179.72(17)

Table 8. Hydrogen bonds for (*S_S*)-**4** [Å and °].

D-H...A	d(D-H)	d(H...A)	d(D...A)	<(DHA)
O(2)-H(2)...O(1)#1	0.82(3)	2.05(3)	2.808(2)	154(3)
O(3)-H(3)...O(1)#1	0.79(4)	2.09(4)	2.834(2)	157(4)

Symmetry transformations used to generate equivalent atoms:

#1 x+1/2,-y+1/2,-z

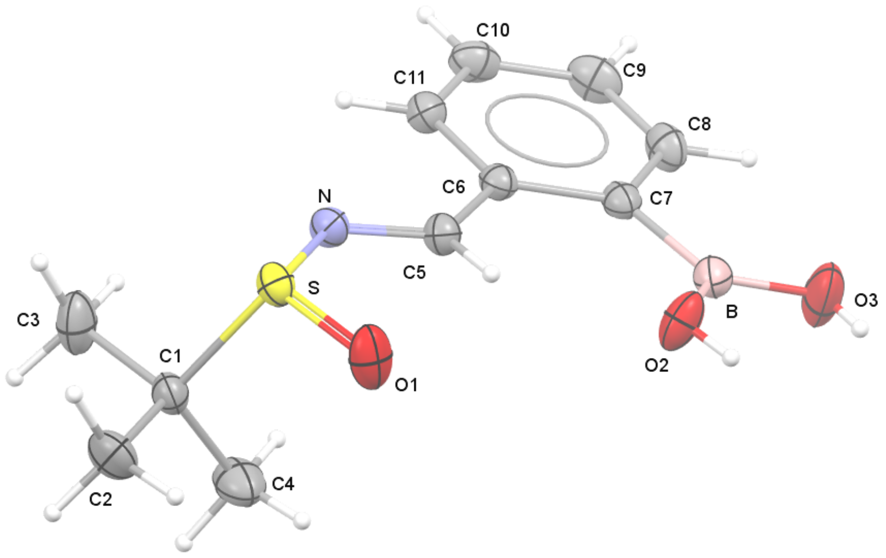

Figure S61: X-ray crystal structure of SIBAs (R_S)-**4**

Crystals suitable for X-ray diffraction were obtained by recrystallisation by slow evaporation of an enantiopure mixture of (R_S)-**4** (*vide supra* for procedure) from 1:1 mixture of $CDCl_3$/*n*-hexane. The crystallographic data in CIF format has been deposited with CCDC: deposition number 2159019.

Table 1. Crystal data and structure refinement for (R_S)-**4**.

Empirical formula	$C_{11}H_{16}BNO_3S$	
Formula weight	253.12	
Temperature	150.01(10) K	
Wavelength	0.71073 Å	
Crystal system	Orthorhombic	
Space group	$P2_12_12_1$	
Unit cell dimensions	a = 9.6026(2) Å	α= 90°.
	b = 11.6066(3) Å	β= 90°.
	c = 11.6791(4) Å	γ = 90°.
Volume	1301.68(6) Å^3	
Z	4	
Density (calculated)	1.292 Mg/m^3	
Absorption coefficient	0.243 mm^{-1}	
F(000)	536	
Crystal size	0.453 x 0.375 x 0.342 mm^3	
Theta range for data collection	3.259 to 30.204°.	
Index ranges	-13<=h<=13, -16<=k<=16, -16<=l<=16	
Reflections collected	22081	
Independent reflections	3612 [R(int) = 0.0321]	

Completeness to theta = 25.242°	99.8 %
Absorption correction	Semi-empirical from equivalents
Max. and min. transmission	1.00000 and 0.93594
Refinement method	Full-matrix least-squares on F^2
Data / restraints / parameters	3612 / 0 / 165
Goodness-of-fit on F^2	1.079
Final R indices [I>2sigma(I)]	R1 = 0.0361, wR2 = 0.0796
R indices (all data)	R1 = 0.0405, wR2 = 0.0812
Absolute structure parameter	-0.01(2)
Extinction coefficient	n/a
Largest diff. peak and hole	0.327 and -0.228 e.$Å^{-3}$

Table 2. Atomic coordinates (x 10^4) and equivalent isotropic displacement parameters (Å^{2}x 10^3) for (R_S)-**4**. U(eq) is defined as one third of the trace of the orthogonalized U^{ij} tensor.

	x	y	z	U(eq)
S	-256(1)	6360(1)	7639(1)	21(1)
O(1)	-136(2)	7014(2)	6540(1)	35(1)
N	1321(2)	5885(1)	8063(2)	22(1)
C(1)	-514(2)	7447(2)	8768(2)	22(1)
C(2)	-1933(2)	7976(2)	8499(2)	34(1)
C(3)	-548(2)	6813(2)	9911(2)	37(1)
C(4)	645(3)	8335(2)	8703(2)	37(1)
C(5)	2313(2)	5955(2)	7345(2)	21(1)
C(6)	3713(2)	5513(2)	7616(2)	19(1)
C(7)	4851(2)	5697(2)	6868(2)	21(1)
C(8)	6141(2)	5247(2)	7203(2)	26(1)
C(9)	6313(2)	4641(2)	8212(2)	31(1)
C(10)	5189(2)	4463(2)	8932(2)	28(1)
C(11)	3897(2)	4894(2)	8639(2)	23(1)
B	4807(3)	6383(2)	5695(2)	25(1)
O(2)	3587(2)	6745(2)	5242(1)	32(1)
O(3)	6058(2)	6578(2)	5189(2)	47(1)

Table 3. Bond lengths [Å] for (R_S)-**4**.

S-O(1)	1.4956(15)
S-N	1.6869(17)
S-C(1)	1.841(2)
N-C(5)	1.272(2)
C(1)-C(4)	1.520(3)
C(1)-C(3)	1.524(3)
C(1)-C(2)	1.527(3)
C(2)-H(2A)	0.9800
C(2)-H(2B)	0.9800
C(2)-H(2C)	0.9800
C(3)-H(3A)	0.9800
C(3)-H(3B)	0.9800
C(3)-H(3C)	0.9800
C(4)-H(4A)	0.9800
C(4)-H(4B)	0.9800
C(4)-H(4C)	0.9800
C(5)-C(6)	1.474(2)
C(5)-H(5)	0.9500
C(6)-C(11)	1.406(3)
C(6)-C(7)	1.415(3)
C(7)-C(8)	1.400(3)
C(7)-B	1.585(3)
C(8)-C(9)	1.382(3)
C(8)-H(8)	0.9500
C(9)-C(10)	1.385(3)
C(9)-H(9)	0.9500
C(10)-C(11)	1.381(3)
C(10)-H(10)	0.9500
C(11)-H(11)	0.9500
B-O(2)	1.352(3)
B-O(3)	1.357(3)
O(2)-H(2)	0.75(3)
O(3)-H(3)	0.71(3)

Table 4. Bond angles [°] for (R_S)-**4**.

O(1)-S-N	110.42(9)
O(1)-S-C(1)	106.10(9)
N-S-C(1)	97.76(9)
C(5)-N-S	117.23(15)
C(4)-C(1)-C(3)	112.75(19)
C(4)-C(1)-C(2)	111.74(19)
C(3)-C(1)-C(2)	110.82(18)
C(4)-C(1)-S	109.29(14)
C(3)-C(1)-S	107.42(15)
C(2)-C(1)-S	104.38(15)
C(1)-C(2)-H(2A)	109.5
C(1)-C(2)-H(2B)	109.5
H(2A)-C(2)-H(2B)	109.5
C(1)-C(2)-H(2C)	109.5
H(2A)-C(2)-H(2C)	109.5
H(2B)-C(2)-H(2C)	109.5
C(1)-C(3)-H(3A)	109.5
C(1)-C(3)-H(3B)	109.5
H(3A)-C(3)-H(3B)	109.5
C(1)-C(3)-H(3C)	109.5
H(3A)-C(3)-H(3C)	109.5
H(3B)-C(3)-H(3C)	109.5
C(1)-C(4)-H(4A)	109.5
C(1)-C(4)-H(4B)	109.5
H(4A)-C(4)-H(4B)	109.5
C(1)-C(4)-H(4C)	109.5
H(4A)-C(4)-H(4C)	109.5
H(4B)-C(4)-H(4C)	109.5
N-C(5)-C(6)	121.27(19)
N-C(5)-H(5)	119.4
C(6)-C(5)-H(5)	119.4
C(11)-C(6)-C(7)	120.33(17)
C(11)-C(6)-C(5)	118.35(17)
C(7)-C(6)-C(5)	121.32(18)
C(8)-C(7)-C(6)	117.04(19)
C(8)-C(7)-B	116.90(18)

C(6)-C(7)-B	126.05(17)
C(9)-C(8)-C(7)	122.3(2)
C(9)-C(8)-H(8)	118.9
C(7)-C(8)-H(8)	118.9
C(8)-C(9)-C(10)	120.1(2)
C(8)-C(9)-H(9)	120.0
C(10)-C(9)-H(9)	120.0
C(11)-C(10)-C(9)	119.73(19)
C(11)-C(10)-H(10)	120.1
C(9)-C(10)-H(10)	120.1
C(10)-C(11)-C(6)	120.57(19)
C(10)-C(11)-H(11)	119.7
C(6)-C(11)-H(11)	119.7
O(2)-B-O(3)	123.0(2)
O(2)-B-C(7)	121.14(18)
O(3)-B-C(7)	115.86(19)
B-O(2)-H(2)	112(3)
B-O(3)-H(3)	110(3)

Table 5. Anisotropic displacement parameters ($Å^2 x 10^3$) for (R_S)-**4**. The anisotropic displacement factor exponent takes the form: $-2\pi^2[h^2 a^{*2}U^{11} + ... + 2 h k a^* b^* U^{12}]$

	U^{11}	U^{22}	U^{33}	U^{23}	U^{13}	U^{12}
S	17(1)	25(1)	22(1)	-2(1)	0(1)	1(1)
O(1)	29(1)	56(1)	19(1)	6(1)	-2(1)	12(1)
N	18(1)	22(1)	26(1)	3(1)	1(1)	3(1)
C(1)	22(1)	24(1)	21(1)	-2(1)	4(1)	3(1)
C(2)	28(1)	33(1)	42(1)	-1(1)	3(1)	11(1)
C(3)	34(1)	54(2)	22(1)	6(1)	6(1)	7(1)
C(4)	34(1)	30(1)	47(1)	-12(1)	6(1)	-7(1)
C(5)	20(1)	22(1)	20(1)	0(1)	0(1)	0(1)
C(6)	20(1)	17(1)	21(1)	-2(1)	1(1)	0(1)
C(7)	19(1)	18(1)	24(1)	-4(1)	2(1)	-2(1)
C(8)	18(1)	30(1)	31(1)	-3(1)	2(1)	1(1)
C(9)	24(1)	32(1)	37(1)	-3(1)	-8(1)	7(1)
C(10)	35(1)	24(1)	27(1)	2(1)	-4(1)	6(1)
C(11)	26(1)	22(1)	22(1)	0(1)	2(1)	0(1)
B	27(1)	23(1)	23(1)	-2(1)	6(1)	-2(1)
O(2)	28(1)	46(1)	23(1)	13(1)	-2(1)	-9(1)
O(3)	34(1)	57(1)	50(1)	25(1)	21(1)	12(1)

Table 6. Hydrogen coordinates (x 10^4) and isotropic displacement parameters ($Å^2$x 10^3) for (R_S)-**4**.

	x	y	z	U(eq)
H(2A)	-2165	8551	9083	52
H(2B)	-2643	7370	8495	52
H(2C)	-1901	8347	7746	52
H(3A)	-770	7359	10525	55
H(3B)	365	6465	10058	55
H(3C)	-1259	6209	9885	55
H(4A)	480	8940	9272	56
H(4B)	663	8677	7936	56
H(4C)	1541	7962	8858	56
H(5)	2147	6300	6620	25
H(8)	6925	5363	6720	32
H(9)	7204	4346	8410	37
H(10)	5306	4047	9626	34
H(11)	3125	4770	9133	28
H(2)	3690(30)	7080(30)	4700(30)	51(10)
H(3)	5960(40)	6880(30)	4660(30)	57(11)

Table 7. Torsion angles [°] for (R_S)-**4**.

O(1)-S-N-C(5)	-11.58(19)
C(1)-S-N-C(5)	-122.03(16)
O(1)-S-C(1)-C(4)	-53.81(18)
N-S-C(1)-C(4)	60.13(17)
O(1)-S-C(1)-C(3)	-176.44(14)
N-S-C(1)-C(3)	-62.50(15)
O(1)-S-C(1)-C(2)	65.85(16)
N-S-C(1)-C(2)	179.79(14)
S-N-C(5)-C(6)	-177.15(13)
N-C(5)-C(6)-C(11)	7.0(3)
N-C(5)-C(6)-C(7)	-173.33(18)
C(11)-C(6)-C(7)-C(8)	-0.5(3)
C(5)-C(6)-C(7)-C(8)	179.77(17)
C(11)-C(6)-C(7)-B	-179.51(18)
C(5)-C(6)-C(7)-B	0.8(3)
C(6)-C(7)-C(8)-C(9)	0.4(3)
B-C(7)-C(8)-C(9)	179.50(19)
C(7)-C(8)-C(9)-C(10)	-0.2(3)
C(8)-C(9)-C(10)-C(11)	0.0(3)
C(9)-C(10)-C(11)-C(6)	-0.1(3)
C(7)-C(6)-C(11)-C(10)	0.4(3)
C(5)-C(6)-C(11)-C(10)	-179.90(19)
C(8)-C(7)-B-O(2)	173.0(2)
C(6)-C(7)-B-O(2)	-8.0(3)
C(8)-C(7)-B-O(3)	-7.0(3)
C(6)-C(7)-B-O(3)	171.9(2)

Table 8. Hydrogen bonds for (R_S)-**4** [Å and °].

D-H...A	d(D-H)	d(H...A)	d(D...A)	<(DHA)
O(2)-H(2)...O(1)#1	0.75(3)	2.11(3)	2.813(2)	155(3)
O(3)-H(3)...O(1)#1	0.71(3)	2.18(3)	2.840(3)	155(4)

Symmetry transformations used to generate equivalent atoms:

#1 x+1/2,-y+3/2,-z+1

11 References

1 R. R. Groleau, R. S. L. Chapman, H. Ley-Smith, L. Liu, T. D. James and S. D. Bull, *J. Org. Chem.*, 2020, **85**, 1208–1215.

2 K. Lawrence, S. E. Flower, G. Kociok-Kohn, C. G. Frost and T. D. James, *Anal. Methods*, 2012, **4**, 2215–2217.

3 A. L. Guzman and T. R. Hoye, *J. Org. Chem.*, 2022, **87**, 905–909.

4 R. Kawęcki, B. Bednarek and J. Sitkowski, *J. Chem. Soc. Perkin Trans. 2*, 2001, **2001**, 1400–1403.

5 S. L. Staun, D. C. Sergentu, G. Wu, J. Autschbach and T. W. Hayton, *Chem. Sci.*, 2019, **10**, 6431–6436.

6 S. Thormeier, B. Carboni and D. E. Kaufmann, *J. Organomet. Chem.*, 2002, **657**, 136–145.

7 T. R. Wu and J. M. Chong, *J. Am. Chem. Soc.*, 2006, **128**, 9646–9647.

8 T. R. Wu and J. M. Chong, *Org. Lett.*, 2006, **8**, 15–18.

9 J. M. Chong, L. Shen and N. J. Taylor, *J. Am. Chem. Soc.*, 2000, **122**, 1822–1823.

10 M. Hatano, T. Sakamoto, T. Mizuno, Y. Goto and K. Ishihara, *J. Am. Chem. Soc.*, 2018, **140**, 16253–16263.

11 M. Hatano, T. Mizuno, A. Izumiseki, R. Usami, T. Asai, M. Akakura and K. Ishihara, *Angew. Chemie Int. Ed.*, 2011, **50**, 12189–12192.

12 M. Jiang, H. Yang and H. Fu, *Org. Lett.*, 2016, **18**, 5248–5251.

13 L. Yu and J. S. Lindsey, *Tetrahedron*, 2001, **57**, 9285–9298.

14 S. Morales, F. G. Guijarro, J. L. G. Ruano and M. B. Cid, *J. Am. Chem. Soc.*, 2014, **136**, 1082–1089.

15 D. Srimani and A. Sarkar, *Tetrahedron Lett.*, 2008, **49**, 6304–6307.

16 Q. Chen, C. Chen, F. Guo and W. Xia, *Chem. Commun.*, 2013, **49**, 6433–6435.

17 J. Vazquez-Chavez, S. Luna-Morales, D. A. Cruz-Aguilar, H. Díaz-Salazar, W. E. V. Narváez, R. S. Silva-Gutiérrez, S. Hernández-Ortega, T. Rocha-Rinza and M. Hernández-Rodríguez, *Org. Biomol. Chem.*, 2019, **17**, 10045–10051.

18 Y. Pérez-Fuertes, A. M. Kelly, J. S. Fossey, M. E. Powell, S. D. Bull and T. D. James, *Nat. Protoc.*, 2008, **3**, 210–214.

19 R. R. Groleau, T. D. James and S. D. Bull, *Coord. Chem. Rev.*, 2021, **428**, 213599.

20 Y. Pérez-Fuertes, PhD Thesis, University of Bath, 2005.

21 S. Arimori, Y. Pérez-Fuertes, S. Lozano Yeste, A. M. Kelly, M. E. Powell, R. R. Groleau, G. Kociok-Köhn, S. D. Bull and T. D. James, *CCDC 2159731 CSD Commun.*

22 S. Arimori, Y. Pérez-Fuertes, S. Lozano Yeste, A. M. Kelly, M. E. Powell, R. R. Groleau, G. Kociok-Köhn, S. D. Bull and T. D. James, *CCDC 2159730 CSD Commun.*
